# DDX56 inhibits PRV replication through regulation of IFN-β signaling pathway by targeting cGAS

**DOI:** 10.3389/fmicb.2022.932842

**Published:** 2022-08-10

**Authors:** Jingying Xie, Xiangrong Li, Shunyu Yang, Zhenfang Yan, Lei Chen, Yanmei Yang, Dianyu Li, Xiangbo Zhang, Ruofei Feng

**Affiliations:** ^1^Key Laboratory of Biotechnology and Bioengineering of State Ethnic Affairs Commission, Biomedical Research Center, Northwest Minzu University, Lanzhou, China; ^2^College of Life Science and Engineering, Northwest Minzu University, Lanzhou, China; ^3^Gansu Tech Innovation Center of Animal Cell, Biomedical Research Center, Northwest Minzu University, Lanzhou, China

**Keywords:** pseudorabies virus, DDX56 protein, innate immune response, IFN-β, cGAS

## Abstract

Pseudorabies virus (PRV) is an agent of Aujeszky's disease, and causes great economic losses to pig farming. Re-outburst of pseudorabies implies that new control measures are urgently needed. We show here that DDX56 possesses the ability to inhibit PRV replication *in vitro*, which may be an important factor for PRV infection. Overexpression of DDX56 inhibited PRV genomic DNA transcription and lower titers of PRV infection in PK15 cells, whereas down-regulation of the DDX56 expression had a promotion role on virus replication. Further study demonstrated that DDX56 exerted its proliferation-inhibitory effects of PRV through up-regulating cGAS-STING-induced IFN-β expression. Moreover, we found that DDX56 could promote cGAS expression and direct interaction also existed between DDX56 and cGAS. Based on this, DDX56-regulated IFN-β pathway may be targeted at cGAS. To verify this, down-regulated cGAS expression in DDX56 over-expression cells was performed. Results indicated that knockdown of cGAS expression could abrogate the inhibition role of DDX56 on PRV proliferation and weaken the effect of DDX56 on IFN-β expression. In addition, DDX56 played a promotion role in IRF3 phosphorylation and nucleus translocation. Altogether, our results highlight DDX56's antiviral role in PRV infection, and our findings contribute to a better understanding of host factors controlling PRV replication.

## Introduction

Pseudorabies virus (PRV), a member of the alpha herpesvirinae subfamily, is responsible for Aujeszky's disease. With a genome of ~140 kb, it encodes more than 70 proteins (Pomeranz et al., 2005). PRV can infect a diverse range of domesticated and wild hosts. It was reported recently that PRV could pose a risk to humans, as indicated by some cases with clinical signs. Pigs are the natural host and reservoir for PRV (Fonseca et al., 2012; Ai et al., 2018). Until now, PRV has been studied in regard to its pathogenesis, but little is known about the host factors affecting viral replication. Therefore, researchers should investigate the host factors that regulate viral replication, and understanding host-virus interactions may lead to the development of more effective antiviral therapies.

The DEAD-box (Asp-Glu-Ala-Asp) proteins are ATP-dependent RNA helicases that contribute to RNA metabolism (Zirwes et al., 2000). These helicases also take part in host immune defense. For example, DDX1, DDX21, and DHX36 form a complex with adaptor protein TRIF in the cytosol to control IFN responses (Zhang et al., 2011a). Also, DDX60, DDX3, and DHX9 are also involved in type I IFN induction and RNA sensing (Oshiumi et al., 2010; Miyashita et al., 2011; Zhang et al., 2011b). There may be many other helicases that possess unidentified antiviral activity. Another RNA helicase from the DEAD-box family, DDX56, is an evolutionary conserved antiviral factor that controls alphavirus infection (Taschuk et al., 2020). By directly interacting with viral proteins or host proteins, the DDX56 protein can affect some viruses' replication. In this respect, it is one of several cellular factors that could affect the replication of certain viruses, for instance, the west Nile virus (WNV) (Xu and Hobman, 2012; Reid and Hobman, 2017), foot and mouth disease virus (FMDV) (Fu et al., 2019), influenza A virus (IAV) (Pirinçal and Turan, 2021), and encephalomyocarditis virus (EMCV) (Xu et al., 2022). Nevertheless, the mechanism by which porcine DDX56 controls PRV infection and its underlying mechanisms have yet to be fully elucidated.

Porcine DDX56 was studied here in relation to PRV infection and its underlying mechanisms. We observed that DDX56 exerts an inhibitory effect on PRV proliferation. To exert its antiviral effects, DDX56 increases cGAS-STING-induced IFN-β production. Deeper research found that DDX56 could promote cGAS expression and interact with cGAS under physiological conditions. Knockdown of cGAS expression could abrogate the inhibition role of DDX56 on PRV proliferation and weaken the effect of DDX56 on IFN-β expression. In addition, DDX56 plays a promotion role in IRF3 phosphorylation and nucleus translocation. Altogether, we gain a better understanding of host control of PRV proliferation with these findings and shed light on understanding the interaction between the host and PRV infection.

## Materials and methods

### Cells and viruses

Porcine kidney (PK15) and baby hamster kidney (BHK-21) cells were used in this study. They were maintained in Dulbecco's modified Eagle's medium (DMEM) supplemented with 10% newborn bovine serum (NBS) and grown at a 37°C/5% CO_2_ incubator. The PRV Bartha-K61 is an attenuated vaccine strain and stored in our laboratory. Bartha-K61 was propagated in BHK-21 cells, and the supernatants of infected cells were clarified and stored at −80°C.

### Antibodies and chemicals

The commercial antibodies used in this study were purchased from the indicated manufacturers. An Anti-FLAG tag rabbit polyclonal antibody (D110005), an Anti-cGAS rabbit polyclonal antibody (D163570), HRP (horseradish peroxidase)-conjugated Goat Anti-Rabbit IgG (D110058), and HRP-conjugated Goat Anti-Mouse IgG (D110087) were used and bought from Sangon Biotech (Shanghai, China). Polyclonal antibody of MAVS (14341-1-AP), HA tag, and IRF3 (11312-1-AP) were purchased from Proteintech (Wuhan, China). Antibody of STING (13647S), TBK1 (3013S), Myc-Tag (2276S), and Phospho-IRF-3 (Ser386) (37829S) were bought from Cell Signaling Technology. Monoclonal Antibody of GAPDH (AF5009) and β-actin (AA128) were obtained from Beyotime Biotechnology (Shanghai, China). Anti-Histone H3 Polyclonal Antibody (K106623P) was purchased from Solarbio (Beijing, China).

Porcine DDX56, MAVS, and cGAS siRNAs were synthesized from RiboBio Co., Ltd (Guangzhou, China). Plasmids encoding DDX56 (Myc tag), and important adaptor proteins in the cGAS-STING pathway, including cGAS (HA tag), STING (HA tag), TBK1 (FLAG tag) and the active form of IRF3 (IRF3/5D) (FLAG tag) were constructed in-house. TransStart®Top Green qPCR SuperMix (+Dye II) was purchased from Transgen (Beijing, China). Premix Ex Taq™ (Probe qPCR) was bought from Takara Biomedical Technology (Beijing, China). Lipofectamine 3000 was purchased from Invitrogen. ISD (tlrl-isdc) and poly (dA:dT) (tlrl-patc) were purchased from InvivoGen.

### Western blotting

Myc-DDX56 expression plasmids were generated by amplifying the DDX56 gene from PK15 cells and cloned into the pCMV-Myc vector. Subsequently, PK15 cells were transfected with the expression plasmid using Lipofectamine® 3000 reagent according to the manufacturer's instructions. Cells were collected and whole-cell extracts were prepared from cells with RIPA lysis buffer (Solarbio, Beijing, China). Cell extracts were subjected to SDS-PAGE and the separated proteins were transferred to the PVDF membranes (Millipore). Skimmed milk diluted in PBS/Tween® 20 was used to block the non-specific antibody binding sites, followed by the specific primary and HRP (horseradish peroxidase)-conjugated secondary antibodies. Detection of the proteins was carried out using an ECL reagent (BioRad, United States) and GAPDH or β-actin was used as loading controls.

### Viral infection

PK15 cells transfection was performed using Lipofectamine 3000 (Invitrogen, United States) according to the manufacturer's instruction. Then, the cells were infected with PRV at 100 TCID_50_. The viral inoculum was removed 2 h later, and the infected cells were cultured in DMEM containing 3% NBS. Cell-free culture supernatants and lysates were harvested at 24-h post-infection to evaluate the effect of DDX56 on PRV replication.

### RNA extraction and RT-qPCR

The IFN-β mRNA transcription level was determined using relative qPCR. Total RNA was extracted and performed as previously described (Xie et al., 2020). All primers used in this study are available upon request.

### Detection and quantitation of the PRV genome

The viral genomic copies of PRV were achieved by quantitative RT-PCR. Viral DNA was isolated using Viral Genomic DNA Extraction Kit (TIANGEN, Beijing, China) and performed as previously described (Xie et al., 2020).

### RNAi assay

siRNA targeting DDX56 was transfected into PK15 cells to verify the effect of DDX56 knockdown. Lipofectamine® 3000 was used to transfect PK-15 cells with siNC or siDDX56 for 24 h, followed by inoculation with 100 TCID_50_ PRV. Cell supernatants were tested for viral titers in BHK-21 cells using the TCID_50_ assay.

### Co-immunoprecipitation assay

Cells were collected with NP40 supplemented with a proteasome inhibitor and incubated with the indicated antibodies for 10 h at 4°C. Each lysate was then treated with 10 μl of Protein G agarose slurry (Beyotime, China). After incubation at 4°C for 4 h, the lysates were centrifuged at 2,500 rpm for 5 min. After collecting the beads, they were washed in ice-cold PBS five times. The precipitate was mixed with SDS buffer and boiled for 5 min at 95°C. After centrifuging at 6,000 rpm for 1 min, the supernatant was analyzed.

### Virus titration

BHK-21 cells were used in these experiments. The related operations were as followed: five replicates of BHK-21 cells were infected with tenfold serial dilutions of PRV, and fresh DMEM was added after 2 h at 37°C. The PRV titers were determined after 48–72 h at 37°C using the Reed-Muench method.

### Enzyme-linked immunosorbent assay

IFN-β secretion expression levels in the cell supernatants were detected using a swine IFN-β enzyme-linked immunosorbent assay (ELISA) kit (Jianglaibio, Shanghai, China) according to the manufacturer's instructions.

### Nuclear and cytoplasmic extraction

PK15 cells were transfected with Myc tagged DDX56 plasmids for 24 h, then the cells were infected with PRV or mock uninfected for another 24 h. Cells were washed with PBS and lysed with 400 μl of extracting solution A with protease inhibitors and phosphatase inhibitors (BestBio, China). The homogenate was centrifuged at 4°C 1,000 g for 5 min. Next, the supernatant was saved as cytoplasm and placed on ice for 30 min. The pellet was re-suspended with 200 μL extracting solution B, which included protease inhibitor and phosphatase inhibitors. 5 μL solution C was also added. Placed on ice for 30 min, and then kept it as the nuclei. The precipitates were analyzed by western blotting using specific antibodies.

### Statistical analysis

ANOVA was used to compare measurements. Student's *t*-test was used to determine statistical significance. Values were expressed in graph bars as the mean ± SD of at least three independent experiments unless otherwise noted. Asterisks denoted statistically significant differences (^***^ *p* < 0.001, ^**^ *p* < 0.01 and ^*^ *p* < 0.05).

## Results

### Overexpression of DDX56 inhibits PRV replication in PK15 cells

**Figure 1:**
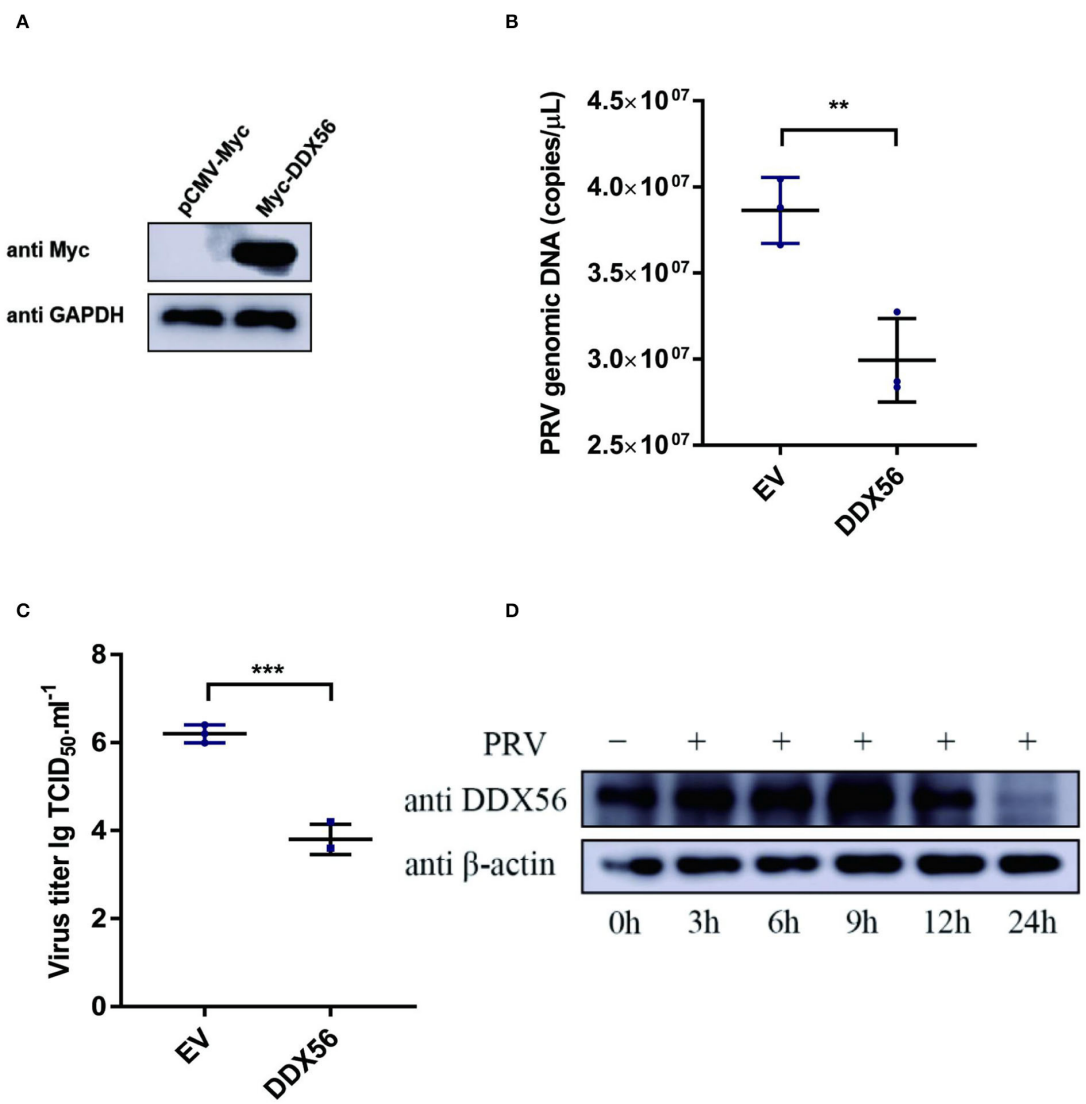
Overexpression of DDX56 inhibits PRV replication in PK15 cells. **(A)** Empty vector (EV, 1 μg) or the pCMV-Myc-DDX56 plasmid (1 μg) was transfected into PK15 cells for 24 h before measuring DDX56 (65 kDa) expression by immunoblotting. GAPDH (37 kDa) served as a loading control. **(B,C)** Empty vector (EV, 1 μg) or the pCMV-Myc-DDX56 plasmid (1 μg) was transfected into PK15 cells for 24 h and then infected with 100 TCID_50_ PRV for 24 h before measuring PRV genomic DNA copy numbers and titers. Viral copy number was determined by Real-Time quantity PCR **(B)** and viral titer was detected by TCID_50_ assay (Reed–Muench method) **(C)**. Data were listed as mean ± SD of three independent experiments and measured in technical duplicate. ***p < 0.001, **p < 0.01. **(D)** PK15 cells were mock-infected or infected with 100 TCID_50_ PRV for 3 h, 6 h, 9 h, 12 h, and 24 h, respectively. Cells were collected at the indicated time points. Western blotting was performed for DDX56 (65 kDa) detection. β-actin (42 kDa) served as loading control.

To test whether DDX56 affects PRV replication in PK15 cells, the plasmid expression Myc-DDX56 was constructed first and transfected into PK15 cells for study. Myc-tagged DDX56 was successfully expressed in PK15 cells (Figure 1A). Cells overexpressing DDX56 were infected with PRV. As a result, PRV DNA copies were lower in DDX56 overexpression cells compared to the control group (Figure 1B). To confirm this result, viral titers were also detected. As Figure 1C shows, there was a notably decreased virus titer in the DDX56-overexpressed cells. Then we tested DDX56 protein expression upon PRV infection. As shown in Figure 1D, DDX56 expression was significantly induced in the PK15 cells at 9 h post-infection. The expression level of DDX56 decreased with the prolongation of infection time (within 9 h). As shown in Figures 1B,C, it had been proved that overexpression of DDX56 could inhibit virus proliferation, but the expression level of DDX56 decreased significantly at 12 h, especially at 24 h after infection. These results suggest that PRV may have a mechanism to inhibit the expression of DDX56 to achieve its proliferation. Taken together, these results indicate that PRV infection could promote DDX56 expression in the early stage and overexpression of DDX56 serves an inhibitory role in PRV multiplication.

### Knockdown of DDX56 promotes PRV proliferation in PK15 cells

**Figure 2:**
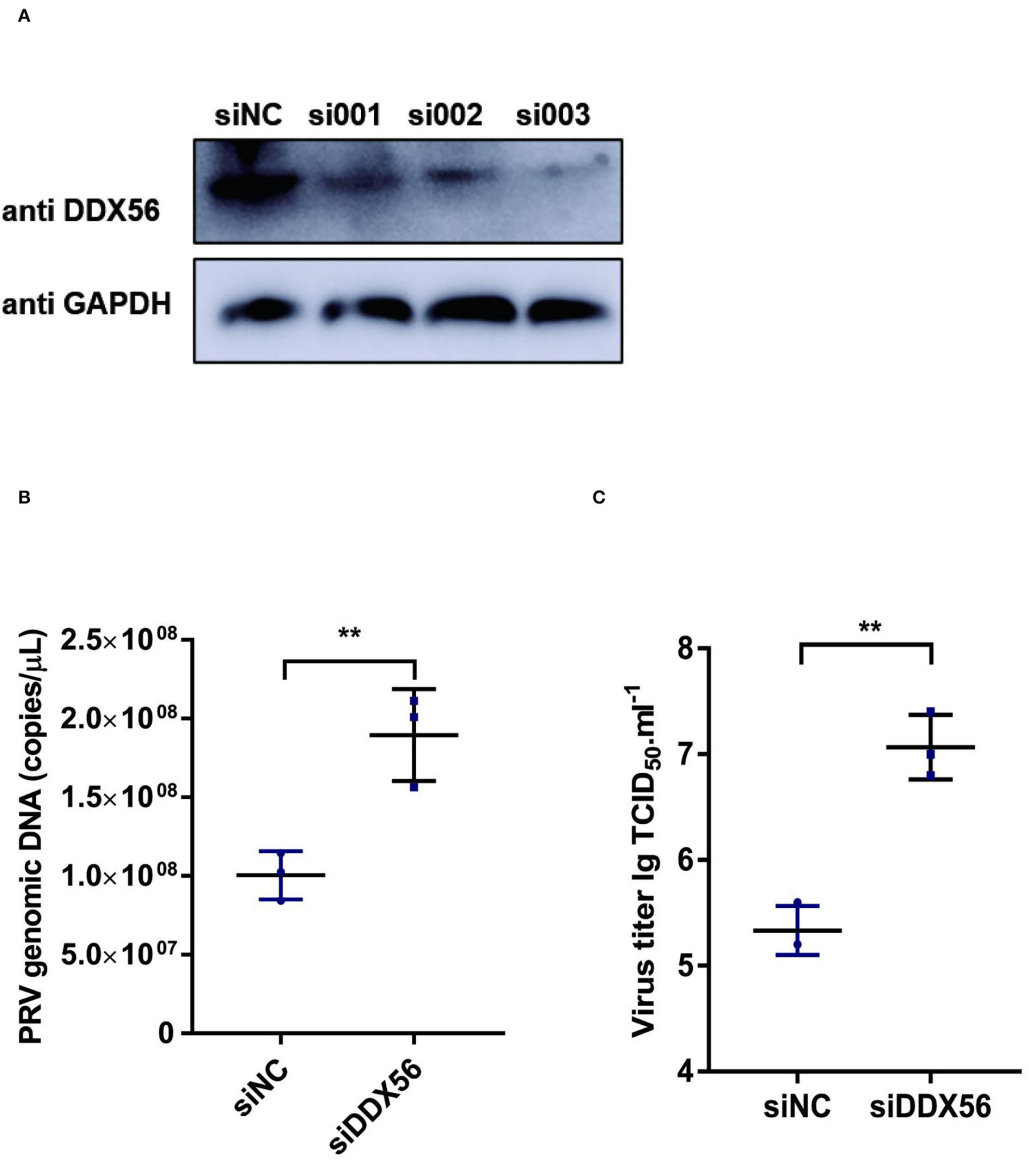
Knockdown of DDX56 promotes PRV proliferation in PK15 cells. **(A)** siRNA targeting DDX56 was transfected into PK15 cells for 24 h. Cells were lysed with RIPA and DDX56 (65 kDa) expression was determined by western blotting using an antibody specific for DDX56. GAPDH (37 kDa) served as loading control. **(B,C)** PK15 cells were transfected with siRNA targeting DDX56 (siRNA Mixture, siDDX56) for 24 h then 100 TCID_50_ PRV was inoculated into these cells for another 24 h before measuring viral copy numbers and titers. Viral copy number was determined by Real-Time quantity PCR **(B)** and viral titer was detected by TCID_50_ assay (Reed–Muench method) **(C)**. Data were presented as mean ± SD of three independent experiments and measured in technical duplicate. ** *p* < 0.01.

To further verify the effect of DDX56 on PRV replication, an RNAi assay was performed in PK15 cells. Results showed that DDX56 expression was decreased significantly in the siRNA transfection group as shown in Figure 2A. Then we analyzed the effect of DDX56 knockdown on PRV infection. As shown in Figure 2B, the viral genomic DNA copies were increased in the siDDX56 group compared to the control siNC group. Besides, the virus titers of the DDX56 knockdown PK15 cells were also higher than that of the siNC control cells (Figure 2C). Together, these data demonstrate that DDX56 knockdown facilitates PRV replication in PK15 cells.

### DDX56 promotes IFN-β expression

**Figure 3:**
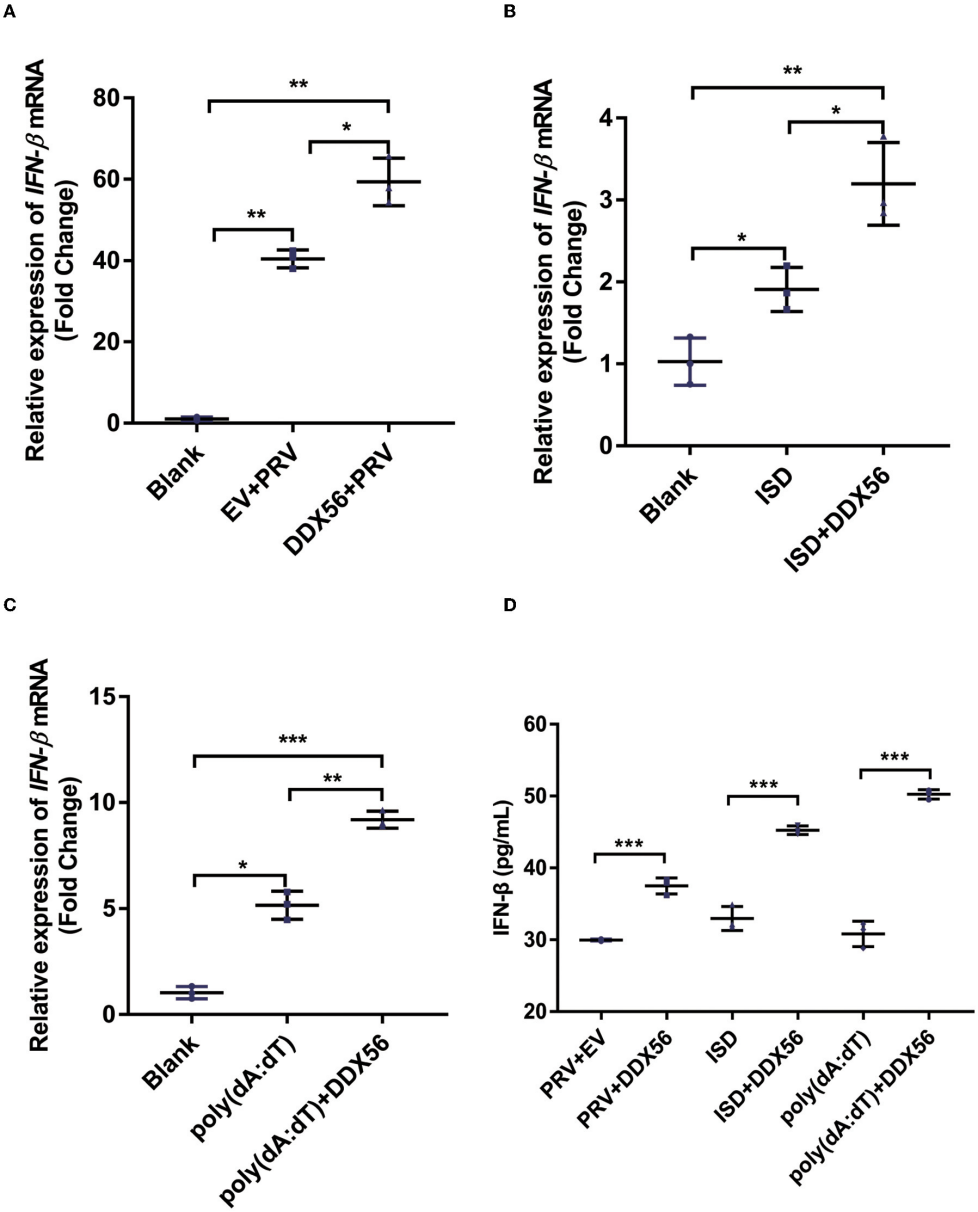
DDX56 promotes IFN-β expression. **(A)** Empty vector (EV, 1 μg) or pCMV-Myc-DDX56 (1 μg) plasmid was transfected into PK15 cells for 24 h, then followed by infection with 100 TCID_50_ PRV for another 24 h. Cells were collected for RNA extraction. RT-qPCR was used for detecting IFN-β mRNA. Data were presented as means ± SD of three independent experiments, measured in technical duplicates. ** *p* < 0.01, * *p* < 0.05. **(B)** Empty vector (EV, 1 μg) or pCMV-Myc-DDX56[[Inline Image]] (1 μg) plasmid was transfected into PK15 cells for 24 h, then stimulated with 1 μg ISD for another 12 h. Cells were collected for RNA extraction. RT-qPCR was used for IFN-β mRNA detection. Data were presented as means ± SD of three independent experiments, measured in technical duplicates. ** *p* < 0.01, * *p* < 0.05. **(C)** Empty vector (EV, 1 μg) or pCMV-Myc-DDX56 (1 μg) plasmid was transfected for 24 h, then stimulated with 1 μg poly(dA:dT) for another 12 h. Cells were collected for RNA extraction. RT-qPCR was used for IFN-β mRNA detection. **(D)** IFN-β concentration in cell culture supernatant was detected by ELISA. Data were presented as mean ± SD of three independent experiments, measured in technical duplicates. *** *p* < 0.001; ** *p* < 0.01, * *p* < 0.05.

The underlying mechanism by which DDX56 interferes with PRV proliferation was investigated by asking if DDX56 could enhance the cellular innate immune response to PRV infection as type I IFN is one of the most important molecules and serves as a potent antiviral effector on viral infection (Chen et al., 2018). Thus, we examined IFN-β expression in DDX56 overexpression PK15 cells during PRV infection as well as in control cells. We found that IFN-β mRNA transcription levels increased in DDX56 overexpression PK15 cells (Figure 3A).

Immunostimulatory DNA (ISD) is a double-stranded DNA 60-mer oligonucleotide derived from the HSV-1 genome and has a high capacity to induce IFN-β production (Unterholzner et al., 2010). ISD serves as an IFN-β activator in this study. We then investigated the effect of DDX56 on ISD-triggered IFN-β transcription. As shown in Figure 3B, DDX56 significantly increased the IFN-β expression when co-transfected with ISD.

Poly(dA:dT) is a poly(deoxyadenylic-deoxythymidylic) acid sodium salt. It is a repetitive synthetic double-stranded DNA sequence of poly(dA-dT):poly(dT-dA) and a synthetic analog of B-DNA. It can be sensed by cytosolic DNA sensors cGAS, and triggers the production of type I interferons (Unterholzner, 2013; Wu et al., 2013). Moreover, DDX56 also significantly increased IFN-β expression when co-transfected with poly (dA:dT) (Figure 3C), supporting the idea that DDX56 is responsible for up-regulating the IFN-β expression. Similar results were obtained by ELISA detection (Figure 3D). Together, these findings suggest that DDX56 helps increase the IFN-β production.

### DDX56 promotes endogenous cGAS expression

**Figure 4:**
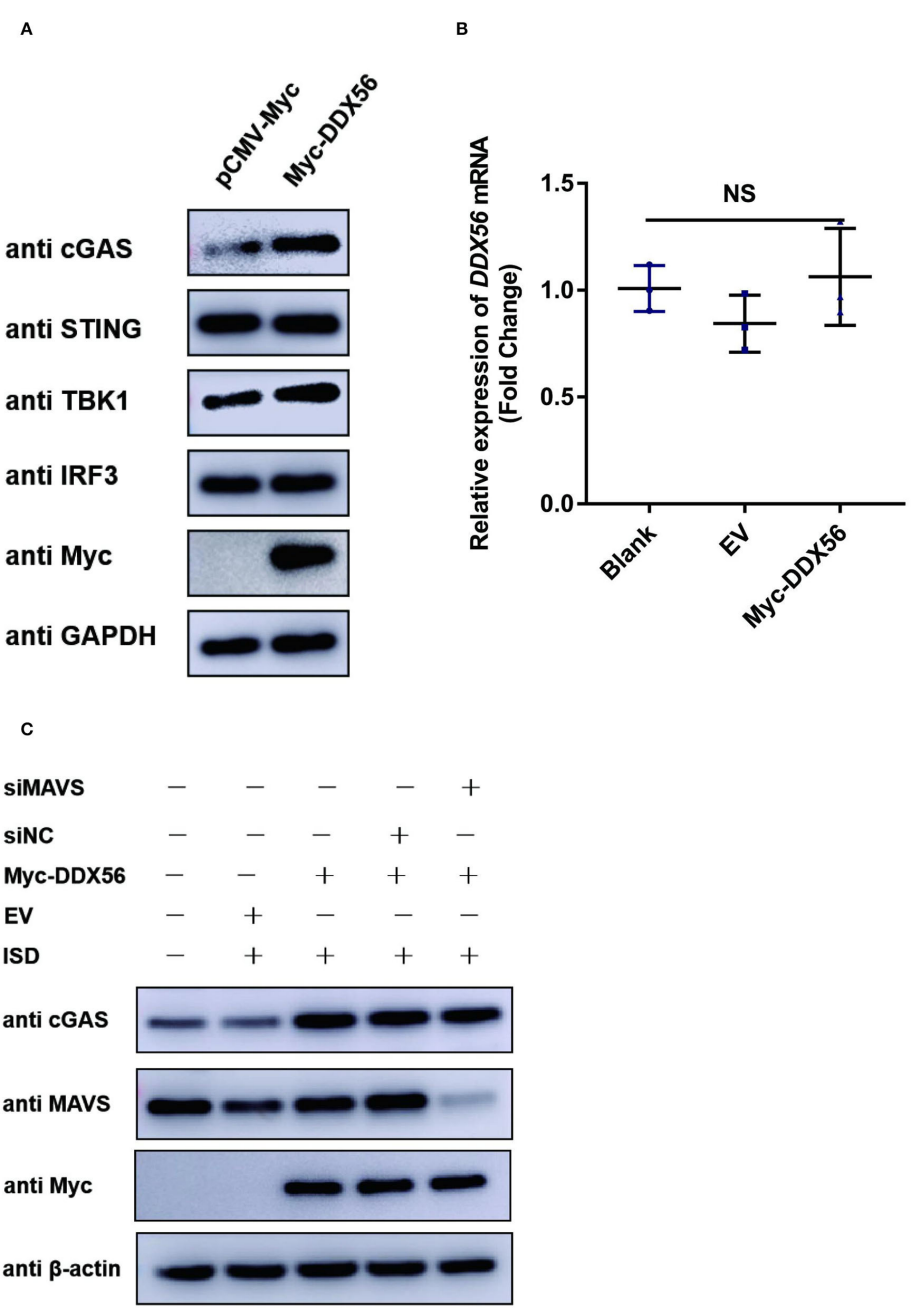
DDX56 promotes endogenous cGAS expression. **(A)** Empty vector (EV, 1 μg) or pCMV-Myc-DDX56 (1 μg) plasmid was transfected into PK15 cells for 24 h, and cells were collected for western blot analysis using specific antibodies to detect the expression of cGAS(58 kDa), STING(42 kDa), TBK1(84 kDa) and IRF3(55 kDa). GAPDH (37 kDa) was used as a loading control. **(B)** PK15 cells were transfected with empty vector (EV, 1 μg) or pCMV-Myc-DDX56 (1 μg) plasmid for 24 h, then cells were collected for cellular RNA extraction. RT-qPCR was performed for IFN-β mRNA detection. Data were presented as mean ± SD of three independent experiments, measured in technical duplicates. NS stands for no significance. **(C)** PK15 cells were transfected with siRNA targeting MAVS (siRNA Mixture, siMAVS) for 24 h, then an empty vector (EV, 1 μg) or pCMV-Myc-DDX56 (1 μg) plasmid was transfected into above cells for another 24 h. Before being collected, cells were stimulated with 1 μg ISD for 12 h. Cellular proteins were extracted and subjected to western blotting for MAVS (56 kDa), cGAS (58 kDa), and Myc tagged DDX56 (65 kDa) detection. β-actin (42 kDa) served as loading control.

PRV is a typical DNA virus and the cGAS-STING pathway is the major sensor for recognizing its viral DNA during virus infection. As shown in Figure 3A, DDX56 could promote PRV-triggered IFN-β activation. In this part, we wanted to know if DDX56 affected molecules associated with the cGAS-STING signaling pathway, including cGAS, STING, TBK1, and IRF3(5D). As exhibited in Figure 4A, the expression level of cGAS was apparently increased in DDX56 overexpression PK15 cells. There was almost no difference in the STING and IRF3 expression between pCMV-Myc and Myc-DDX56 transfection group. Since cGAS is an interferon-stimulated gene, and DDX56 overexpression may stimulate IFN production, we wondered if the upregulation of cGAS is an effect resulting from increased IFN expression. We first examined the effect of DDX56 overexpression alone on IFN-β mRNA transcription, and RT-qPCR detection found that DDX56 overexpression alone did not affect IFN-β expression (Figure 4B). For DNA virus, Toll-like receptor 9 (TLR9), cyclic GMP-AMP (cGAMP) synthase (cGAS), DAI (DLM-1/ZBP1), absent in melanoma 2 (AIM2), and IFN gamma-inducible protein 16 (IFI16) serve as the main PRRs that recognize viral DNA (Brennan and Bowie, 2010; Xia et al., 2016). PRRs then recruit a series of signal transduction molecules, such as myeloid differentiation primary response gene 88 (MyD88), mitochondrial antiviral-signaling protein (MAVS), and intracellular stimulator of IFN genes (STING). These proteins then transfer the different signals to the downstream molecules in different signaling pathways, which eventually lead to activation and translocation of several transcription factors, including NF-κB, interferon regulatory factor 3 (IRF3), and IRF7 into the nucleus to induce the expression of IFN-I and proinflammatory cytokines (Koyama et al., 2008; Hu and Shu, 2020; Wan et al., 2020). As MAVS is a key linker molecule of type I IFN signaling pathway, we performed DDX56 overexpression in the context of RNAi MAVS expression, and then examined the expression of cGAS at the same time. Interestingly, we found that there was still a significant increase in the expression of cGAS in the presence of knockdown MAVS expression (Figure 4C). Altogether, we can clearly conclude that DDX56 overexpression can promote the expression of cGAS and that this process is independent of the increased expression of IFN-β.

### cGAS might be the potential target of DDX56 protein

**Figure 5:**
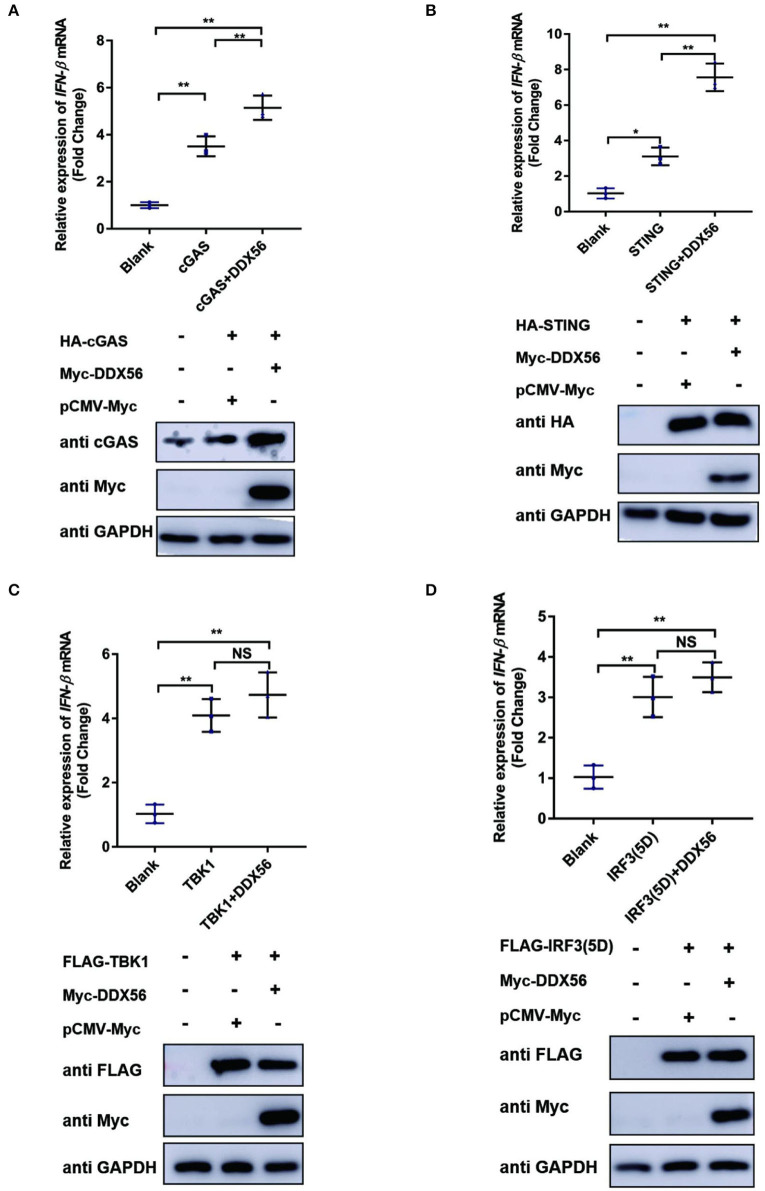
cGAS might be the potential target of DDX56 protein. In PK15 cells, the empty vector (EV, 1 μg) or pCMV-Myc-DDX56 plasmids (1 μg) was co-transfected with indicated plasmids encoding cGAS (250 ng) **(A)**, STING (250 ng) **(B)**, TBK1 (250 ng) **(C)** or IRF3 (5D) (250 ng) **(D)** for 30 h. Then the RNA was extracted and IFN-β levels were determined by RT-qPCR. Western blotting was used to assess the expression of several adaptor molecules as well as the DDX56 protein. GAPDH was used as a loading control. Data were presented as mean ± SD of three independent experiments, measured in technical duplicates. ** *p* < 0.01 and * *p* < 0.05.

To further formalize the effect of DDX56 on the expression of cGAS, we then co-transfected DDX56 and molecules belonging to the cGAS-STING signaling pathway. As shown in Figure 5, DDX56 could promote cGAS expression compared to the control group (Figure 5A). There was no effect on the expression of exogenous STING, TBK1, and IRF3 (Figures 5B,D). These results were consistent with the detection of endogenous correlation factors (Figure 4). Taken together, these results indicate that DDX56 is responsible for the increase of cGAS expression.

Virus infection is blocked by the innate immune response system in which the IFN signaling pathway plays an essential role. To examine the function of DDX56 on cGAS-STING-triggered IFN-β transcription, an RT-qPCR assay was performed and the results are exhibited in Figure 5. DDX56 could promote cGAS and STING-triggered IFN-β activation (Figures 5A,B). However, DDX56 had no effect on IFN-β activation induced by TBK1 and IRF3 (5D) (Figures 5C,D). Combined with western blotting detection results, we speculate that cGAS might be a DDX56 function target to enhance the IFN-β signaling pathway.

### DDX56 interacts directly with cGAS in PK15 cells

**Figure 6:**
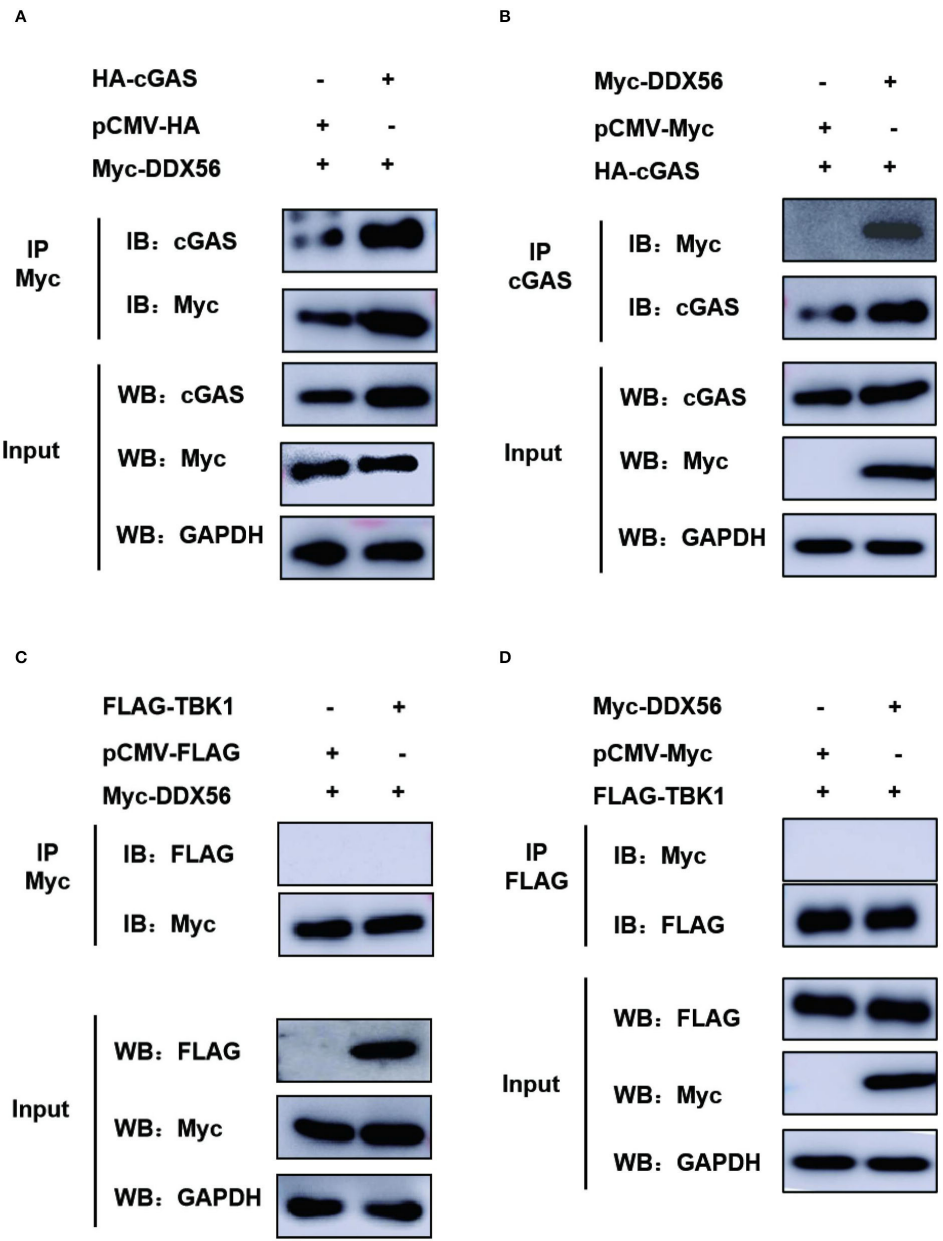
There is a direct interaction between DDX56 and cGAS. **(A,B)** The interaction between DDX56 and cGAS was confirmed by a co-immunoprecipitation assay. In PK15 cells, empty vector (EV, 1 μg) or pCMV-Myc-DDX56 (1 μg) plasmids and the indicated plasmids expressing cGAS (250 ng) were co-transfected for 30 h. Using a lysis buffer supplemented with phosphatase inhibitor cocktail, cells were collected and protein G agarose was incubated with anti-Myc or anti-cGAS antibody. Whole-cell lysates (input) and immunoprecipitation (IP) complexes were analyzed using specific antibodies by western blotting. **(C,D)** The interaction between DDX56 and TBK1 was confirmed by a co-immunoprecipitation assay. In PK15 cells, empty vector (EV, 1 μg) or pCMV-Myc-DDX56 (1 μg) plasmids and the indicated plasmids expressing TBK1 (250 ng) were co-transfected for 30 h. Using a lysis buffer supplemented with phosphatase inhibitor cocktail, cells were collected and protein G agarose was incubated with anti-Myc or anti-FLAG antibody. Whole-cell lysates (input) and immunoprecipitation (IP) complexes were analyzed using specific antibodies by western blotting.

cGAS plays an extremely important role in type I IFN production in response to PRV infection (Wang et al., 2018). As DDX56 protein showed a significant promotion effect on cGAS expression and its triggered IFN-β activation (Figure 5A), suggesting that it could target cGAS to enhance the host innate immune response. An anti-Myc antibody was used for the co-immunoprecipitation (Co-IP) assay to determine if DDX56 binds to cGAS after co-transfection of HA-cGAS and Myc-DDX56 plasmids. As shown in Figure 6A, it was found that DDX56 co-precipitated with cGAS, suggesting a direct interaction between the two proteins. Co-IP was performed to confirm the interaction between DDX56 and endogenous cGAS by transfecting PK15 cells with Myc-vector or Myc-DDX56-expressing plasmids along with HA-cGAS. An anti-cGAS antibody was used to detect and visualize the cGAS expression. As shown in Figure 6B, DDX56 protein was immunoprecipitated with endogenous cGAS.

We then also proved if DDX56 interacted with TBK1 under physiological conditions. PK15 cells were transfected with Myc-DDX56 and FLAG-TBK1. Then Co-IP experiments were performed using Myc tag antibody or FLAG tag antibody. As shown in Figures 6C,D, there were no bands detected in FLAG tag antibody detection or Myc tag antibody after Co-IP. These results demonstrate that DDX56 interacts directly with cGAS and DDX56 targets cGAS to promote host immune response.

### cGAS is essential for DDX56 to promote IFN-β pathway activation

**Figure 7:**
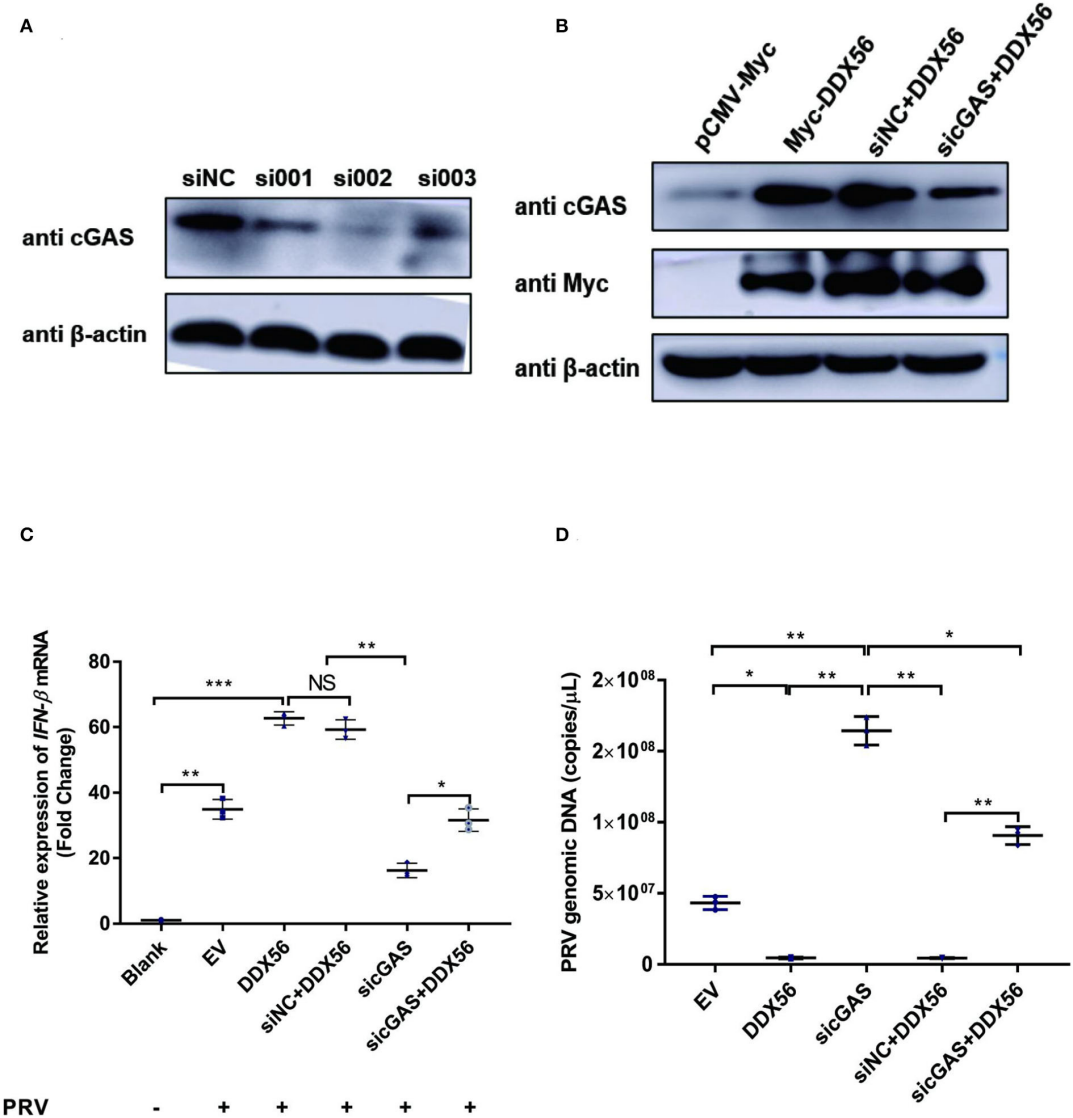
cGAS is essential for DDX56 to promote IFN-β pathway activation. **(A)** siRNA targeting cGAS was transfected into PK15 cells for 24 h. Then cells were lysed with RIPA and cGAS expression was determined by western blotting using an antibody specific for cGAS (58 kDa). β-actin (42 kDa) served as a loading control. **(B)** siRNA targeting cGAS was transfected into PK15 cells for 24 h. Then cells were transfected with empty vector (EV, 1 μg) or pCMV-Myc-DDX56 (1 μg) plasmids. 24 h later, cells were collected and lysated with RIPA. Endogenous cGAS (58 kDa) and Myc tagged DDX56 (65 kDa) protein expression were detected by western blotting. β-actin (42 kDa)served as a loading control. **(C)** siRNA targeting cGAS was transfected into PK15 cells for 24 h. Then cells were transfected with empty vector (EV, 1 μg) or pCMV-Myc-DDX56 (1 μg) plasmids for another 24 h. PRV infection was performed at 100 TCID_50_. Cells were collected for RNA extraction 24 h post-infection. RT-qPCR was performed for IFN-β mRNA detection. Data were presented as mean ± SD of three independent experiments, measured in technical duplicates. *** *p* < 0.001, ** *p* < 0.01 and * *p* < 0.05. **(D)** siRNA targeting cGAS was transfected into PK15 cells for 24 h. Then cells were transfected with empty vector (EV, 1 μg) or pCMV-Myc-DDX56 (1 μg) plasmids for another 24 h. PRV infection was performed at 100 TCID_50_. Cell culture supernatant was collected and viral genomic DNA was extracted. Real-Time quantity PCR was used to determine viral copy number. Data were presented as mean ± SD of three independent experiments, measured in technical duplicate. ** *p* < 0.01 and * *p* < 0.05.

As DDX56 could promote cGAS expression and there was also a direct interaction between DDX56 and cGAS, we wanted to know if cGAS was a key adaptor in DDX56-inhibited PRV infection. siRNAs targeting cGAS were designed and synthesized. Then siRNAs were transfected into PK15 cells to evaluate their role in knockdown cGAS expression. As shown in Figure 7A, siRNA targeting cGAS could effectively decrease cGAS protein expression. Then siRNAs mixture was used for the following experiments. DDX56-overexpressed PK15 cells were transfected with sicGAS and siNC served as the negative control. The expression of Myc tagged DDX56 and cGAS was proved by western blotting (Figure 7B). Then the cells were infected with PRV. IFN-β and viral genomic copies number were detected. Knockdown cGAS expression efficiently decreased DDX56-enhanced IFN-β mRNA transcription (Figure 7C). In Figure 7D, DDX56 overexpression could inhibit PRV replication. But when RNAi of cGAS expression was performed using sicGAS in the DDX56 overexpression group, we found PRV copies number was higher than that in the siNC transfection group. These data demonstrate that cGAS is the target molecule of DDX56 that promotes-β expression and inhibits PRV replication.

**Figure 8:**
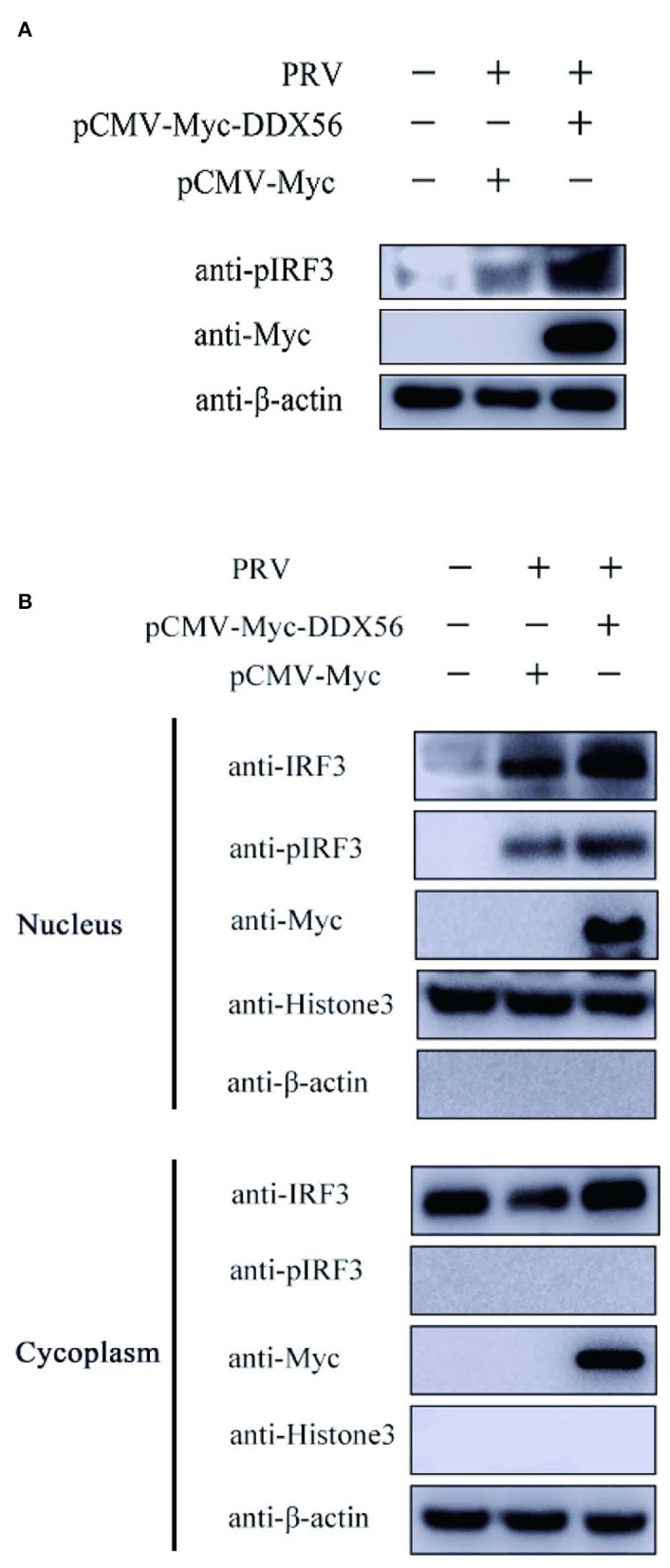
DDX56 promotes IRF3 phosphorylation and nuclear translocation induced by PRV. **(A)** PK15 cells were transfected with empty vector (EV, 1 μg) or pCMV-Myc-DDX56 (1 μg) plasmid for 24 h, then cells were infected with 100 TCID_50_ PRV for 12 h. Cells were collected for IRF3 phosphorylation (55 kDa) detection. β-actin (42 kDa) served as a loading control. **(B)** PK15 cells were transfected with empty vector (EV, 1 μg) or pCMV-Myc-DDX56 (1 μg) plasmid for 24 h. Cells were then stimulated with 100 TCID_50_ PRV for 12 h before collection. Nuclear and Cytoplasmic Extraction was used to separate DDX56 proteins, or IRF3 protein in the cytoplasm and nucleus, then DDX56 (65 kDa), IRF3 (55 kDa), and IRF3 phosphorylation level (55 kDa) were confirmed by immune blotting using specific antibodies. β-actin (42 kDa) was used as a loading control in cytoplasma and Histone 3 (17 kDa) was used as a loading control in the nucleus.

In our previous study of DDX56 on EMCV replication, we found that DDX56 had a different effect on EMCV proliferation (Xu et al., 2022). To make clear how the same protein exerts contradictory effects on different classes of viruses, we tested the effects of DDX56 on IRF3 phosphorylation during PRV infection. Results are shown as Figure 8A. DDX56 did not affect IRF3, but it promoted PRV-induced IRF3 phosphorylation. IRF3 nuclear translocation is a precondition for IFN production. Hence, we checked the effect of DDX56 expression on IRF3 nuclear translocation. PK15 cells were transfected with Myc-DDX56 for 24 h before treating with PRV for a further 24 h. As expected, DDX56 expression promoted IRF3 translocation into the nucleus (Figure 8B). Together, these findings demonstrate that DDX56 facilitates IFN-β production through interacting with cGAS and advances the phosphorylation and nuclear translocation of IRF3.

## Discussion

In the swine industry, PRV is a destructive pathogen causing enormous losses. Understanding what factors control PRV proliferation is essential to better understanding host-virus interactions. The inhibitory role of DDX56 in PRV infection may provide potential targets for antiviral therapies. Here, we demonstrate that DDX56 overexpression significantly reduced the replication of PRV infection, whereas DDX56 knockdown increased viral growth. These findings suggest that DDX56 is a potentially useful target to effectively control PRV infection.

Pathogen-associated molecular patterns (PAMPs) are recognized by host cellular recognition receptors (PRRs) and trigger IFNs and pro-inflammatory cytokines during virus infection. All these responses are beneficial to viral replication inhibition, infected cells clearance, and the adaptive immune response orchestration, even to eliminate infected pathogens (Kawai and Akira, 2006; Carpenter et al., 2014; Beachboard and Horner, 2016; Chen et al., 2017). A cytosolic DNA sensor, Cyclic GMP-AMP (cGAMP) synthase (cGAS) activates the type I IFN response in response to pathogen DNA. cGAS catalyzes the synthesis of cGAMP from pathogen DNA, eliciting an IFN response, then activates the endoplasmic reticulum (ER)-anchored stimulator of interferon genes (STING). The STING translocates from the ER to the Golgi apparatus, where it recruits and phosphorylates TANK-binding kinase 1 (TBK1) and IκB kinase (IKK). These events activate IRF3 and NF-κB, which in turn activate type I IFN production (Fitzgerald et al., 2003; Sharma et al., 2003; Sun et al., 2013; Xia et al., 2016).

It has been shown that DDX56 can regulate the natural immune response signaling pathway, especially the IFN-β pathway (Fu et al., 2019; Xu et al., 2022). We first detected the role of DDX56 on PRV, ISD, and poly(dA:dT)-induced IFN-β activation. Results showed that DDX56 significantly promoted PRV, ISD, and poly(dA:dT)-triggered IFN-β transcription. Our study examined how DDX56 affected cGAS-STING-TBK1, since this axis is crucial for host defense against DNA viruses (Kato et al., 2017). Then we tested the effect of DDX56 on cGAS-STING signaling pathway-associated factors that triggered IFN-β expression. Results indicated that DDX56 indeed promoted IFN-β expression, especially cGAS and STING-induced IFN-β expression. Interestingly, DDX56 also increased cGAS expression and interacted with cGAS. In the ZCCHC3 protein study, scientists found that ZCCHC3 could interact with cGAS and promote the binding of cGAS to DNA (Lian et al., 2018). In this study, DDX56 presented an anti-PRV activity. Further studies are necessary to determine whether DDX56 binds to PRV DNA or promotes the activation of cGAS. Furthermore, if DDX56 deletion impairs innate response *in vivo* during PRV infection still needs to be further investigated in the future.

In our previous study of DDX56 on EMCV replication, we found that DDX56 could directly interact with viral protein to antagonize virus-triggered IFN-β production and promote EMCV proliferation (Xu et al., 2022). In this study, we found that DDX56 had an inhibitory role on PRV infection. Whether viral proteins are involved in this process or whether DDX56 interacts with viral proteins is unknown and needs further investigation at a later stage.

To validate if cGAS is the key factor for DDX56 regulating-IFN-β pathway, siRNAs targeting cGAS were designed and synthesized. Knockdown of cGAS expression by RNAi, followed by overexpression of DDX56 in these cells, showed that IFN-β transcription was decreased during PRV infection and viral copies number was increased compared to DDX56 overexpression group. These data revealed that DDX56 targets cGAS to exert its antiviral effect. We all know that cGAS is an important DNA sensor during PRV virus infection (Wang et al., 2018), and maybe DDX56 is a co-sensor of cGAS for PRV recognition.

**Figure 9:**
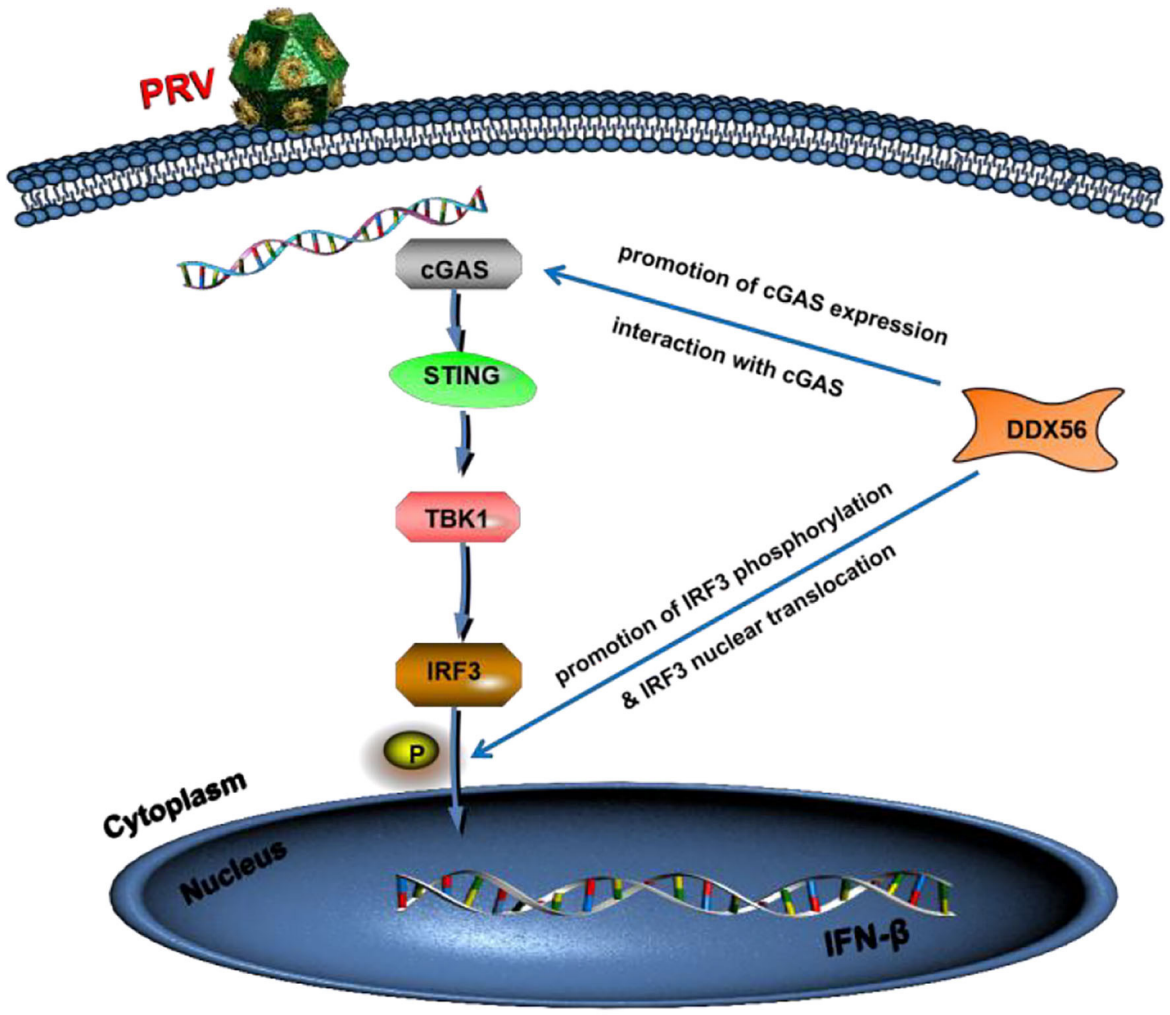
Model of DDX56 promotes IFN-β pathway activation to inhibit PRV infection. Cellular protein DDX56 promotes cGAS expression and interacts with cGAS. Further study shows DDX56 does not affect IRF3, but it promotes PRV-induced IRF3 phosphorylation. As expected, DDX56 expression promoted IRF3 translocation into the nucleus. Together, these findings demonstrate that DDX56 plays a positively regulation role in IFN-β production through interacting with cGAS and promoted the phosphorylation and nuclear translocation of IRF3.

To summarize, we showed that porcine DDX56 expression inhibited PRV proliferation in PK15 cells (Figure 9). Overexpression of DDX56 markablely inhibited PRV replication and the decreased DDX56 expression had a promotion role on PRV infection. Mechanistically, DDX56 upregulated IFN-β expression and then resulted in suppressing viral proliferation. Further mechanism exploration revealed that DDX56 could induce cGAS expression and there was a direct interaction between DDX56 and cGAS. These data demonstrated that cellular protein DDX56 targets cGAS to exert its antiviral effect. In addition, DDX56 also promoted IRF3 phosphorylation and its nuclear translocation. Further research is needed to determine if DDX56's ATPase or helicase activities are responsible for its inhibition of PRV replication. Together, these results illustrate the critical role of DDX56 during PRV infection and these results contribute to understanding the host-virus interaction that occurs during PRV infection.

## Data availability statement

The original contributions presented in the study are included in the article/Supplementary Material, further inquiries can be directed to the corresponding author/s.

## Author contributions

JX, XL, and RF contributed to the conception and design of the study. JX, XL, SY, and ZY did the experiments. LC, XZ, YY, and DL performed the statistical analysis. JX and XL wrote the first draft of the manuscript. SY, ZY, and LC wrote sections of the manuscript. All authors contributed to manuscript revision, read, and approved the submitted version.

## Funding

This work was supported by Open Funds of the Biomedical Research Center from Northwest Minzu University (EB202101), the Fundamental Research Funds for the Central Universities (31920220068), and the Young Doctor Fund Project of Gansu Province Education Department (2021QB-064).

## Conflict of interest

The authors declare that the research was conducted in the absence of any commercial or financial relationships that could be construed as a potential conflict of interest.

## Publisher's note

All claims expressed in this article are solely those of the authors and do not necessarily represent those of their affiliated organizations, or those of the publisher, the editors and the reviewers. Any product that may be evaluated in this article, or claim that may be made by its manufacturer, is not guaranteed or endorsed by the publisher.
