# Supplementary material for: DDX56 inhibits PRV replication through regulation of IFN-β signaling pathway by targeting cGAS
Source: Front Microbiol. 2022 Aug 10;13:932842. doi: 10.3389/fmicb.2022.932842 (PMC9450509; doi:10.3389/fmicb.2022.932842)
Supplement: Supplementary file 1 [file Data_Sheet_1.doc]

**Figure 1A**

**
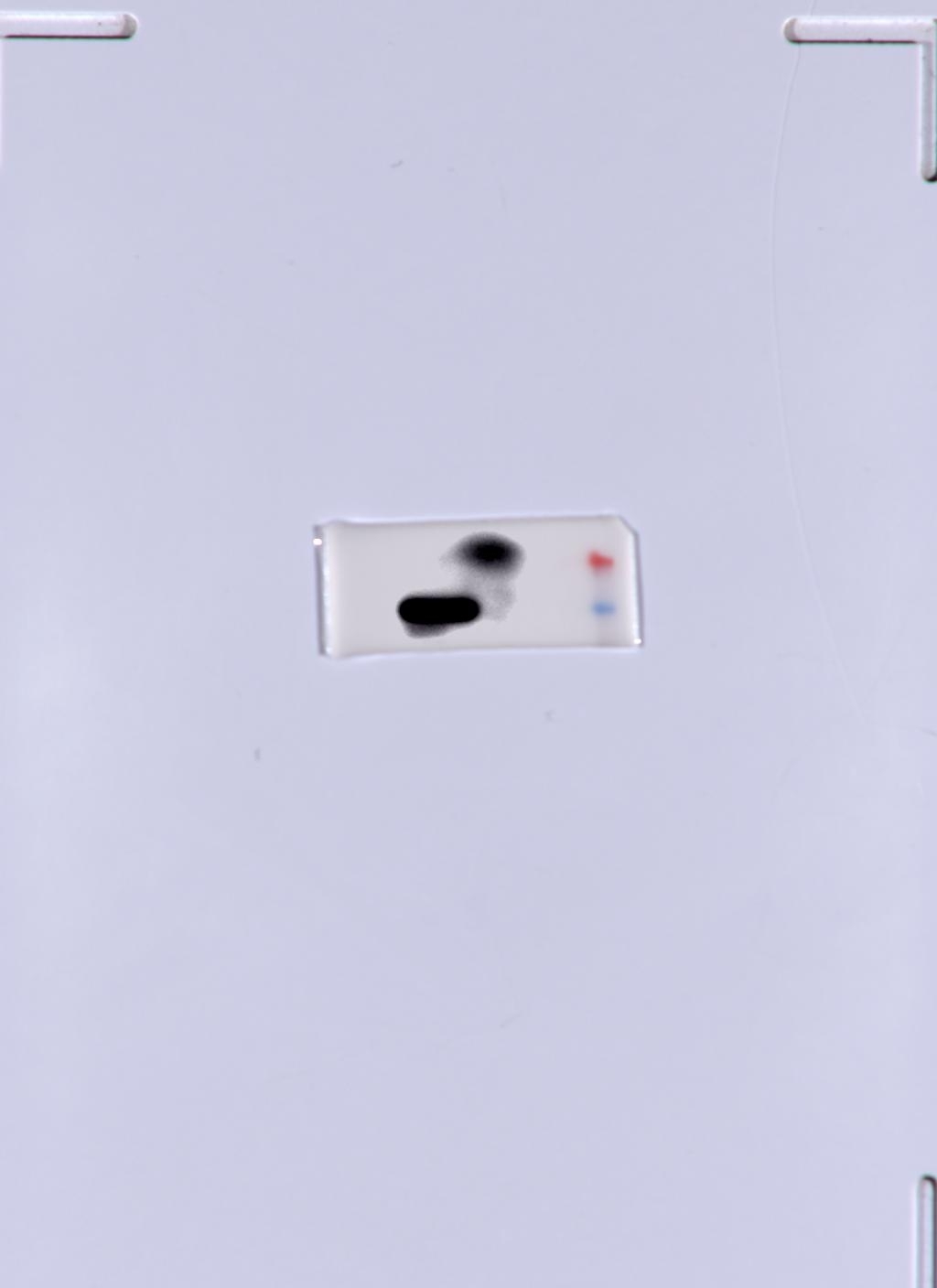

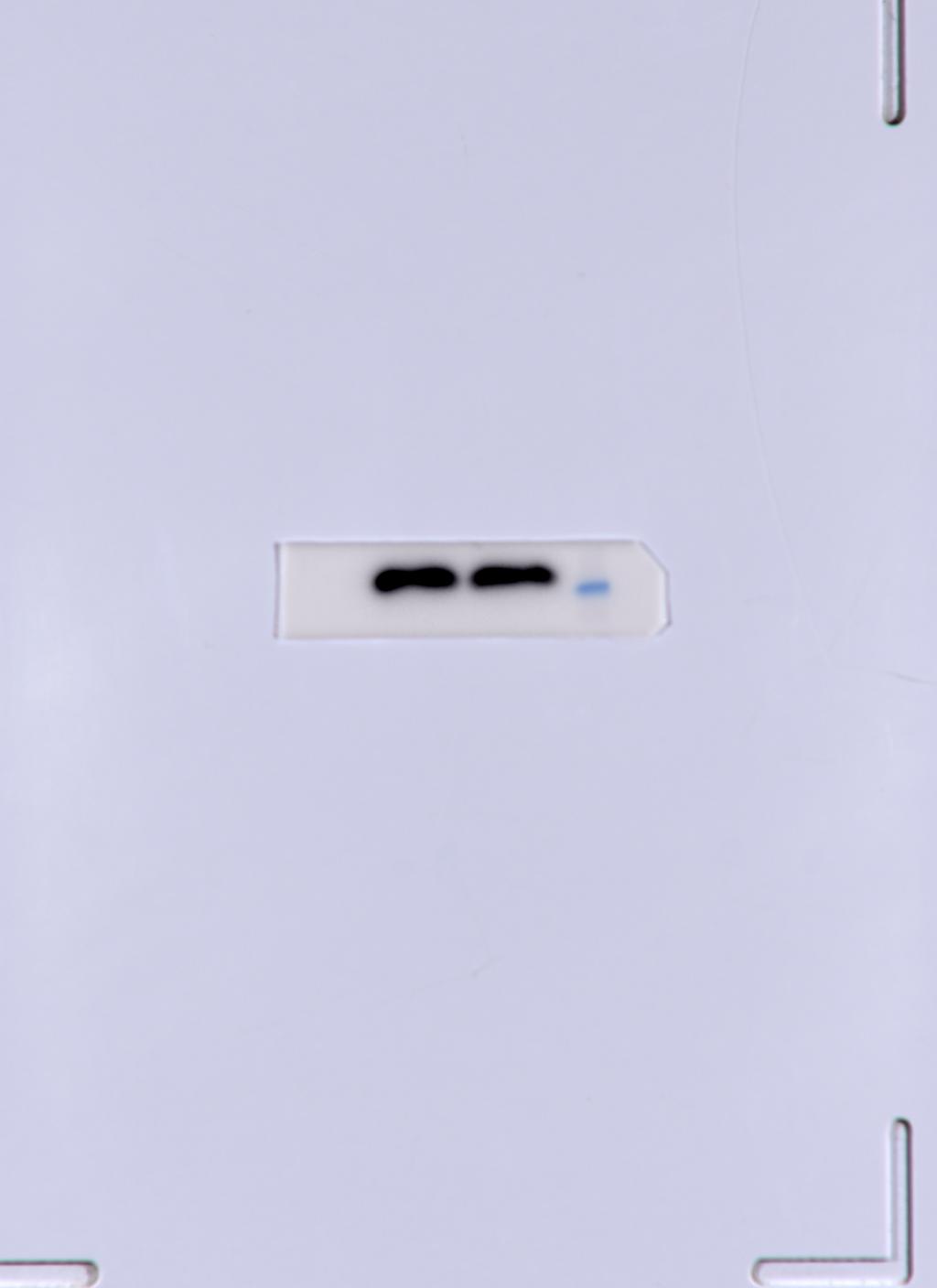
**

**anti Myc GAPDH**

**1B**

| EV | DDX56 |
| --- | --- |
| 4.0457589e+007 | 2.837919e+007 |
| 3.8815037e+007 | 2.8707806e+007 |
| 3.6643757e+007 | 3.2734069e+007 |

**1C**

| EV | DDX56 |
| --- | --- |
| 6.4 | 3.6 |
| 6.2 | 4.2 |
| 6 | 3.6 |

**Figure 2A**

**
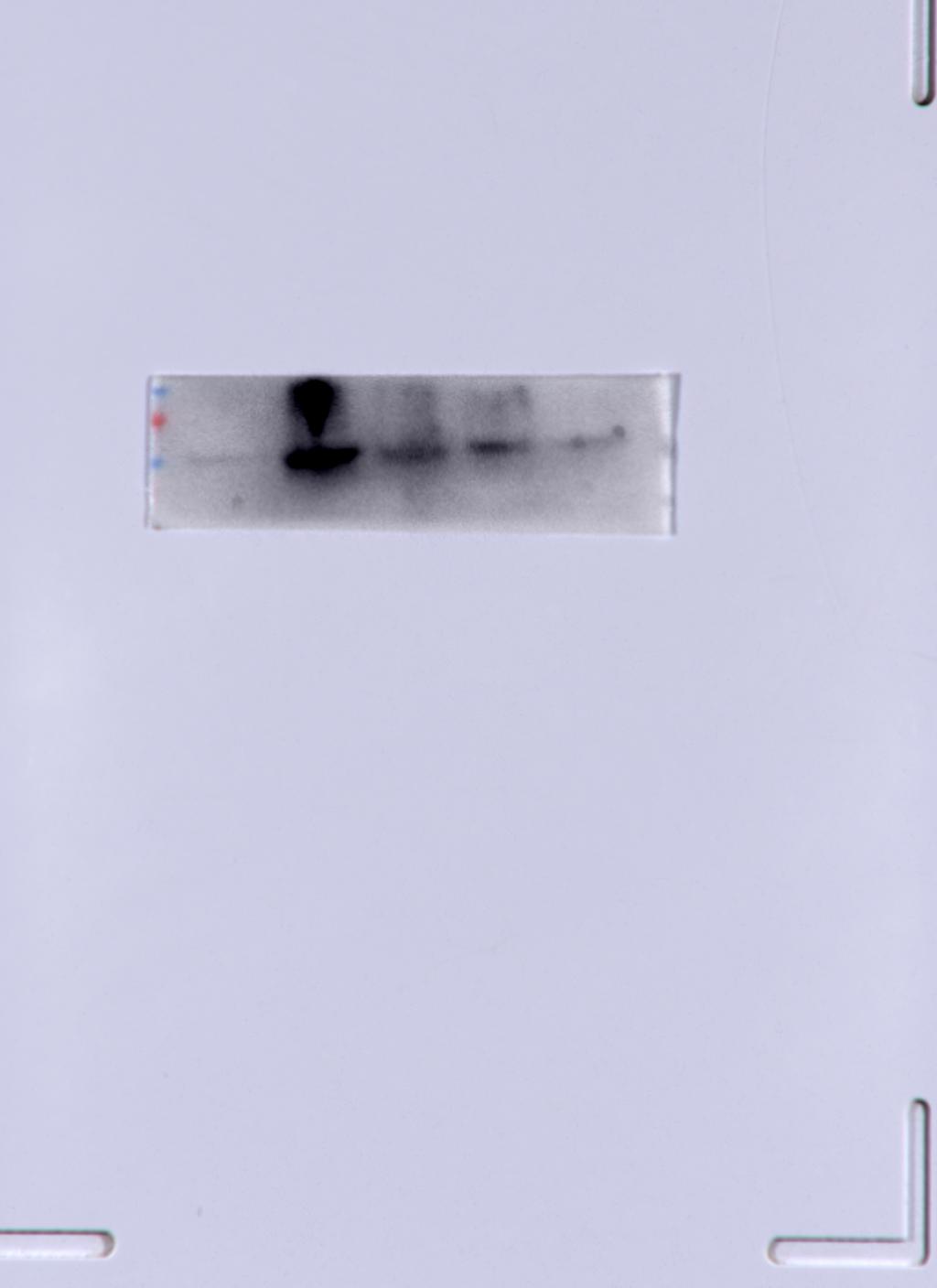

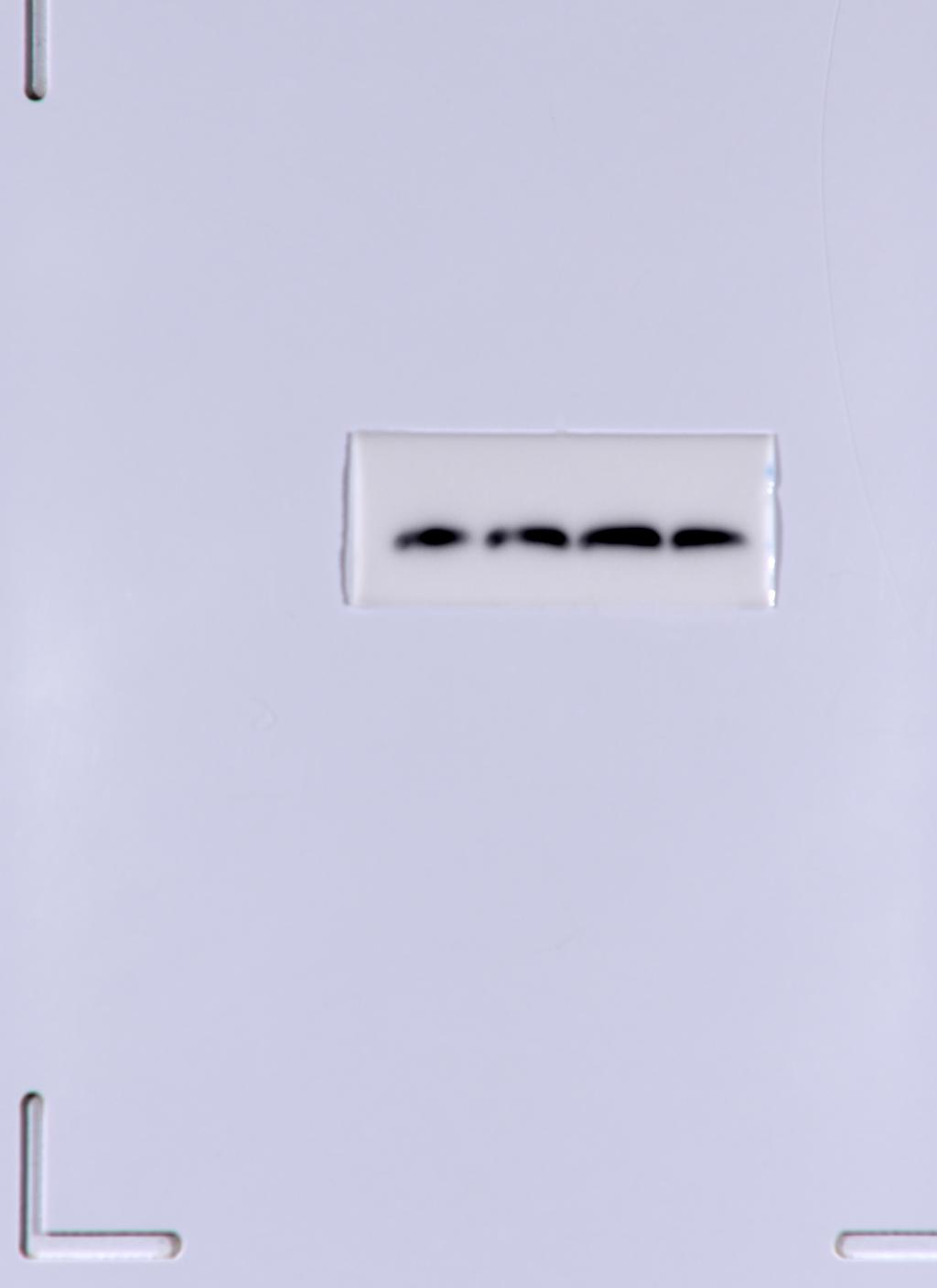
**

**anti DDX56 GAPDH**

**2B**

| siNC | siDDX56 |
| --- | --- |
| 8.419765576e+007 | 1.562787756e+008 |
| 1.14419488e+008 | 2.009555477e+008 |
| 1.026360666e+008 | 2.113002447e+008 |

**2C**

| siNC | siDDX56 |
| --- | --- |
| 5.6 | 7.4 |
| 5.2 | 6.8 |
| 5.2 | 7 |

**Figure 3**

**3A**

| Blank | EV+PRV | DDX56+PRV |
| --- | --- | --- |
| 1 | 38.055 | 57.9042 |
| 0.667201 | 40.81074 | 54.364 |
| 1.498798 | 42.41073 | 65.78526 |

**3B**

| Blank | ISD | ISD+DDX56 |
| --- | --- | --- |
| 1 | 1.66396 | 2.969419 |
| 0.752489 | 1.86571 | 2.845919 |
| 1.328923 | 2.198258 | 3.776729 |

**3C**

| Blank | poly(A:T) | poly(A:T)+DDX56 |
| --- | --- | --- |
| 1 | 5.787488 | 9.647941 |
| 0.752489 | 4.471727 | 8.894951 |
| 1.328923 | 5.210945 | 9.03555 |

**Figure 4**

**
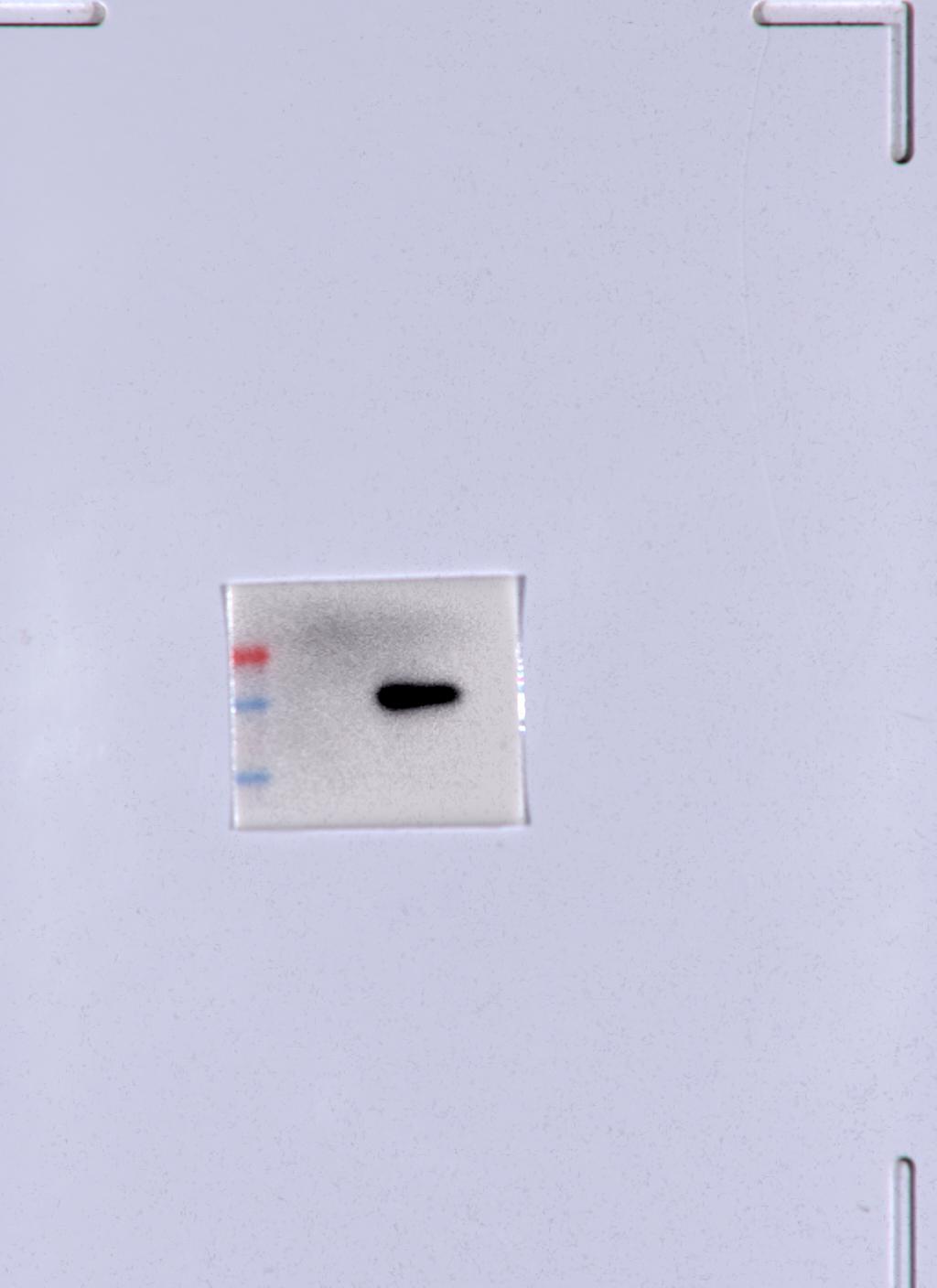

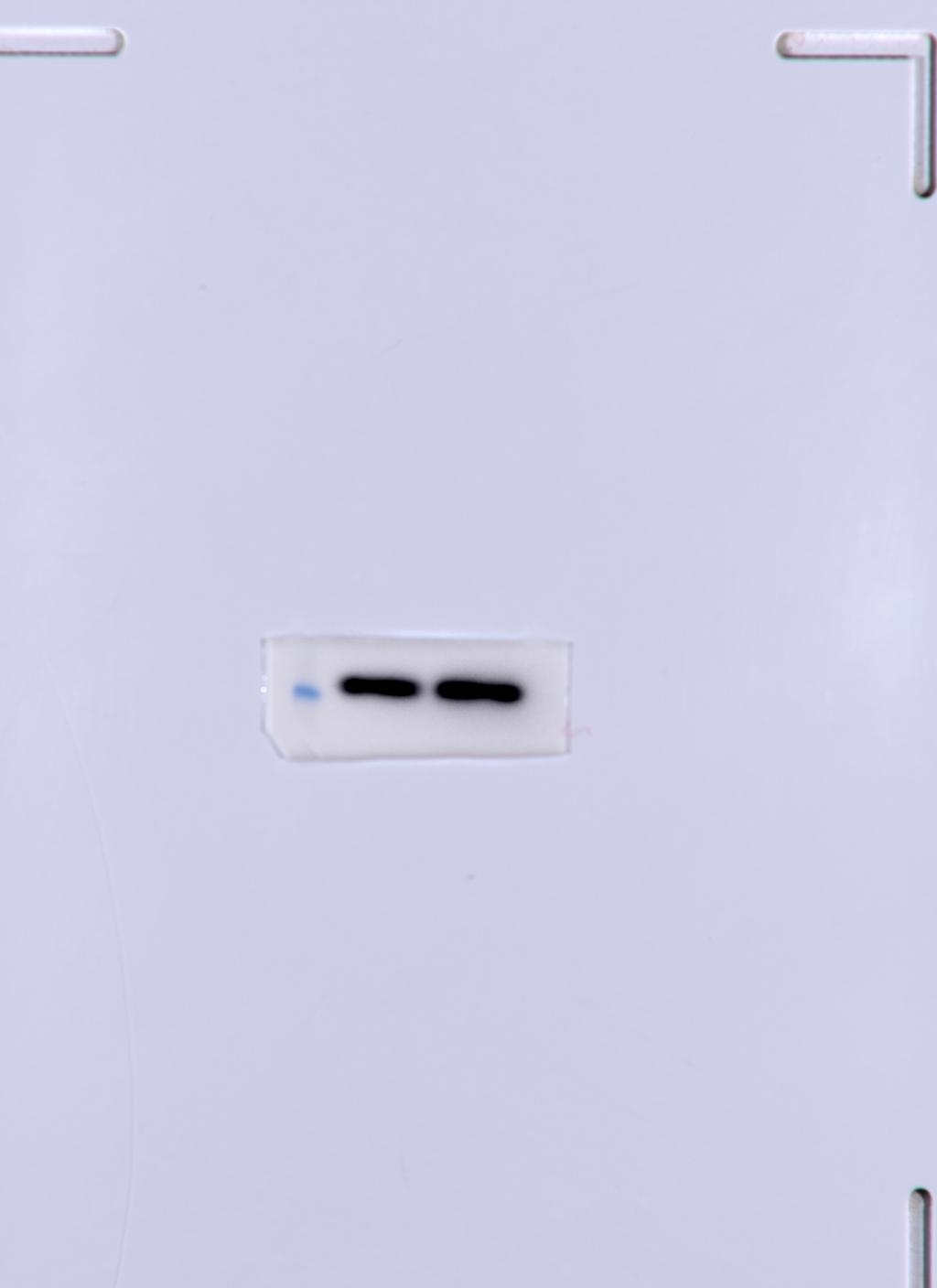

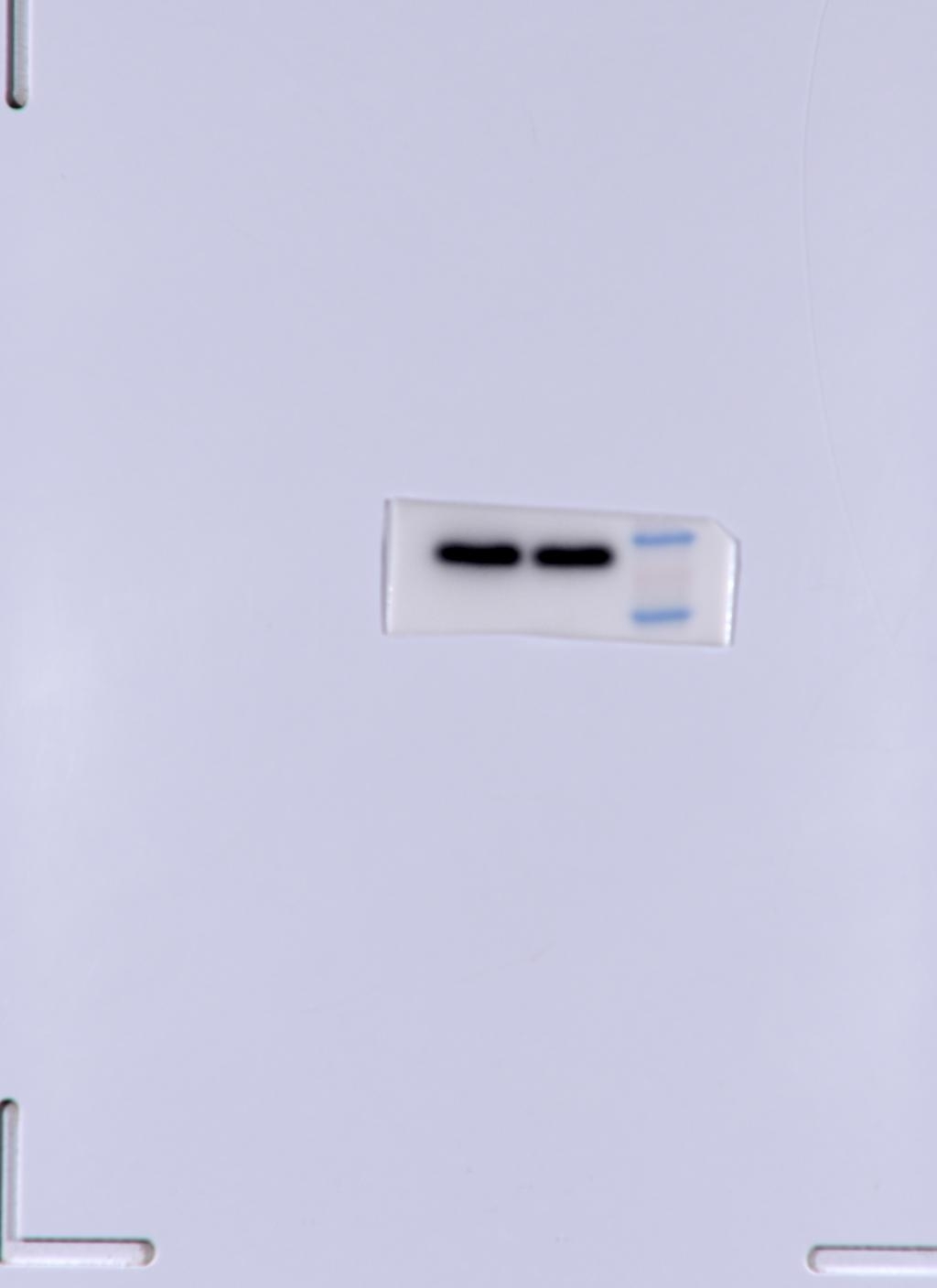
**

**anti Myc GAPDH IRF3**

**
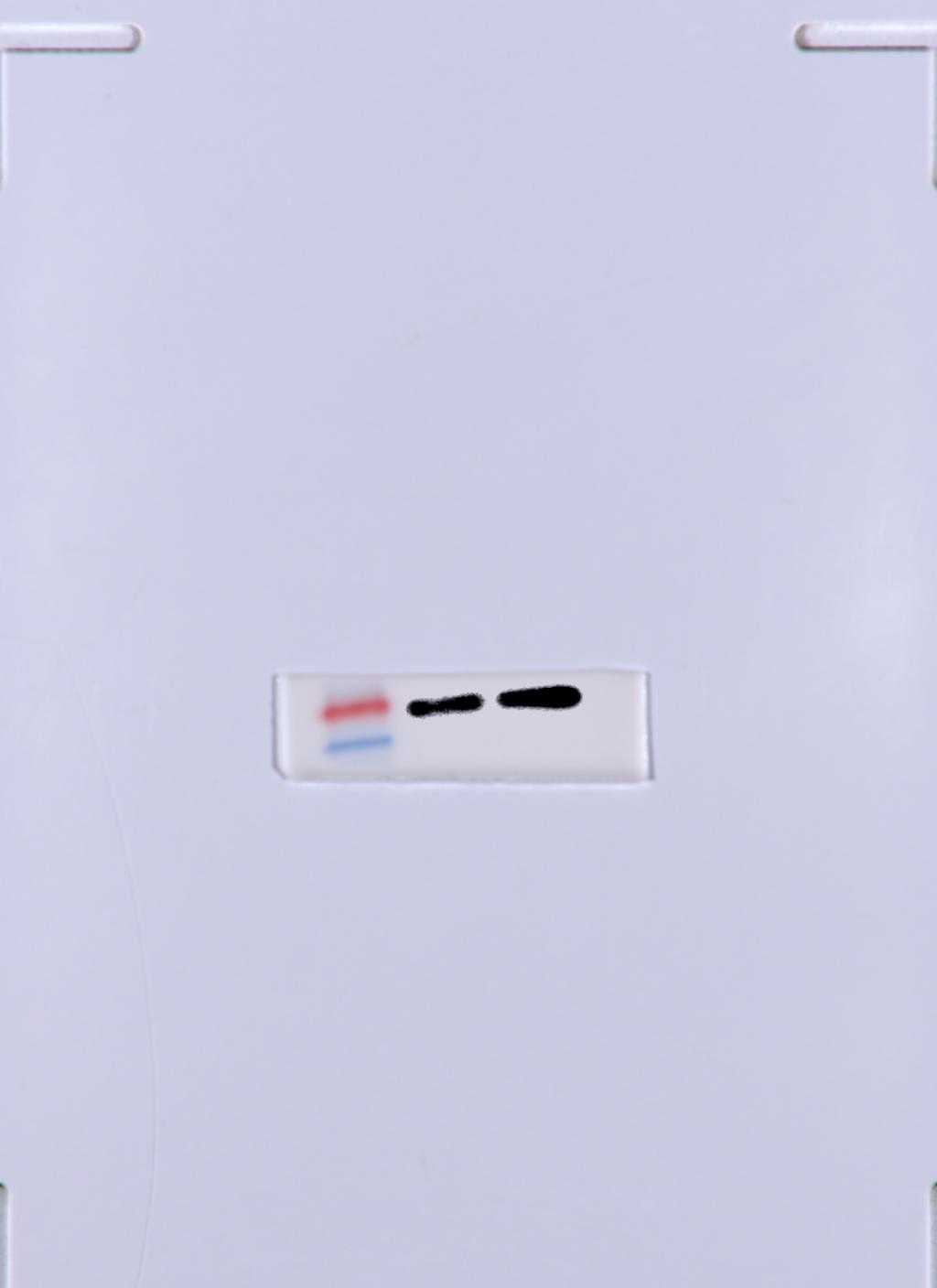

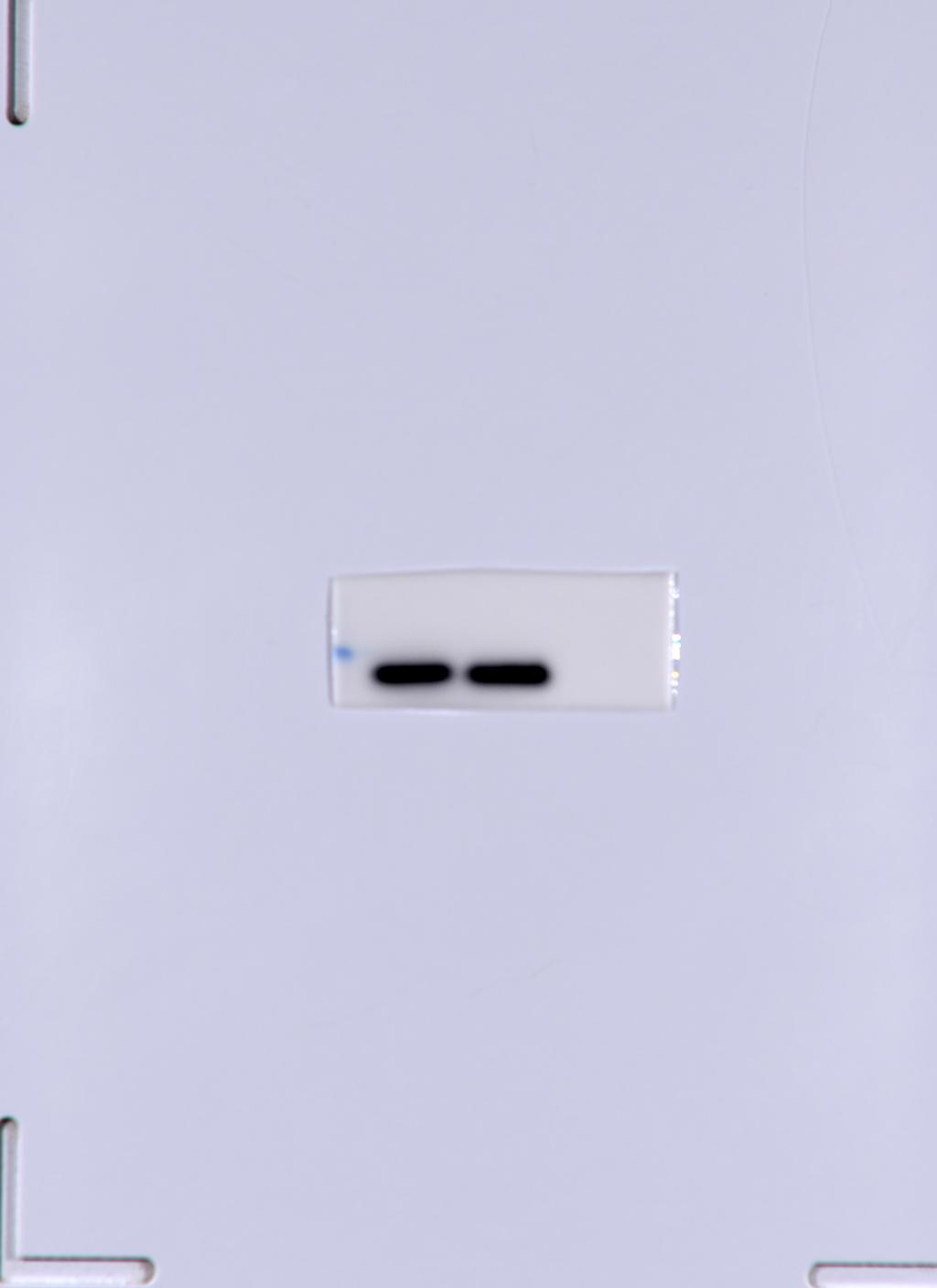

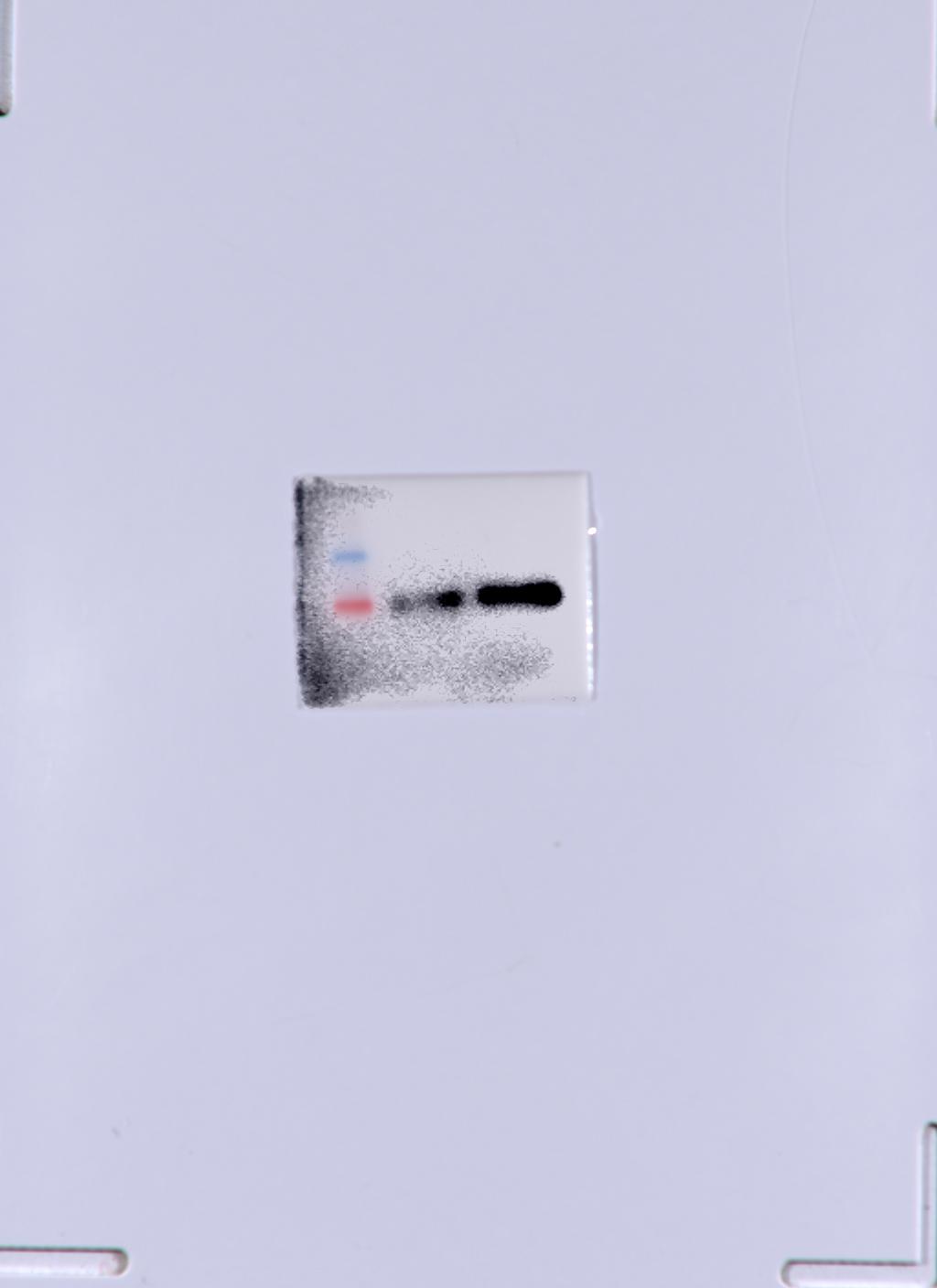
**

**TBK1 STING cGAS**

**Figure 5**

**A&B**

**
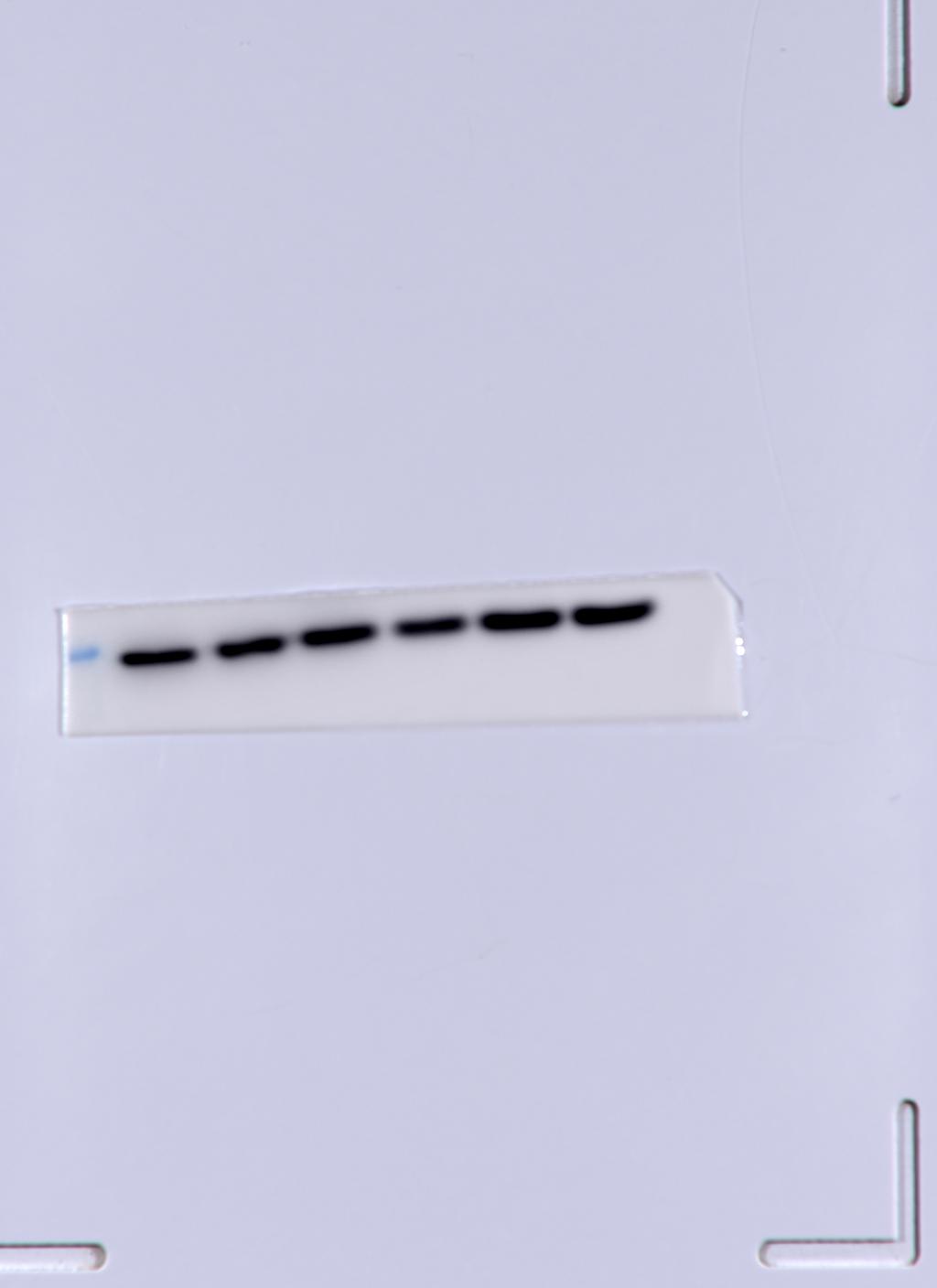

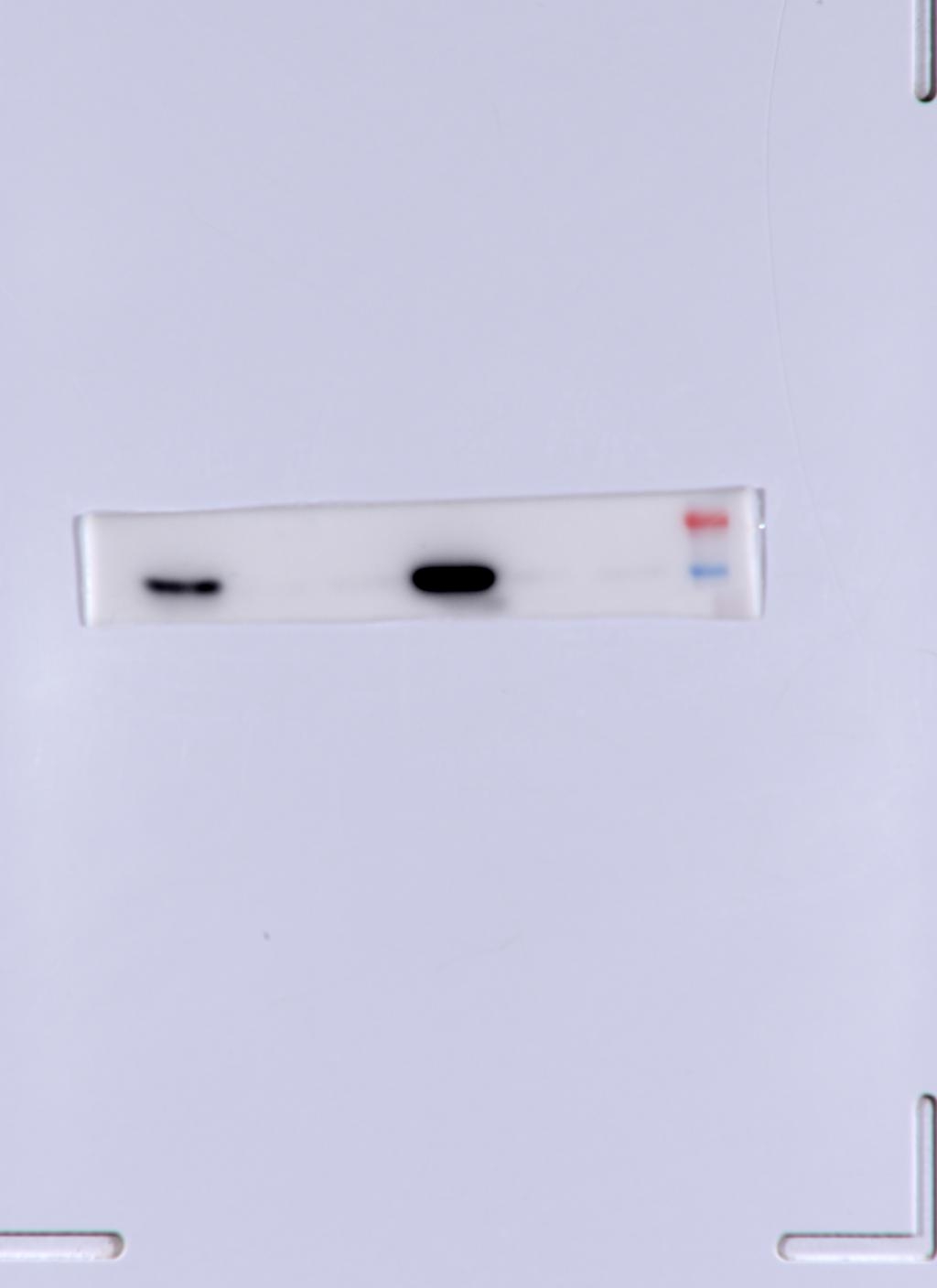

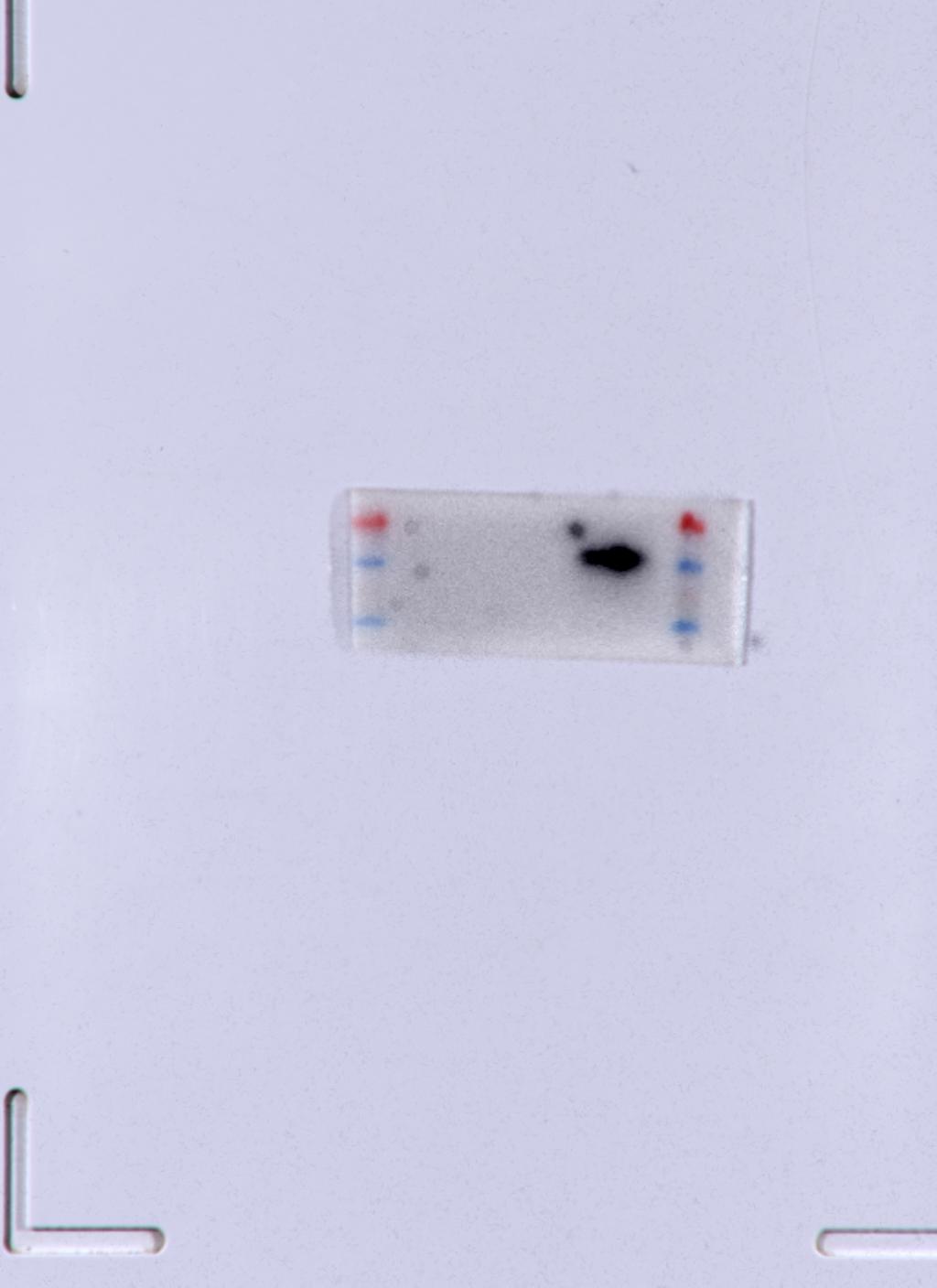

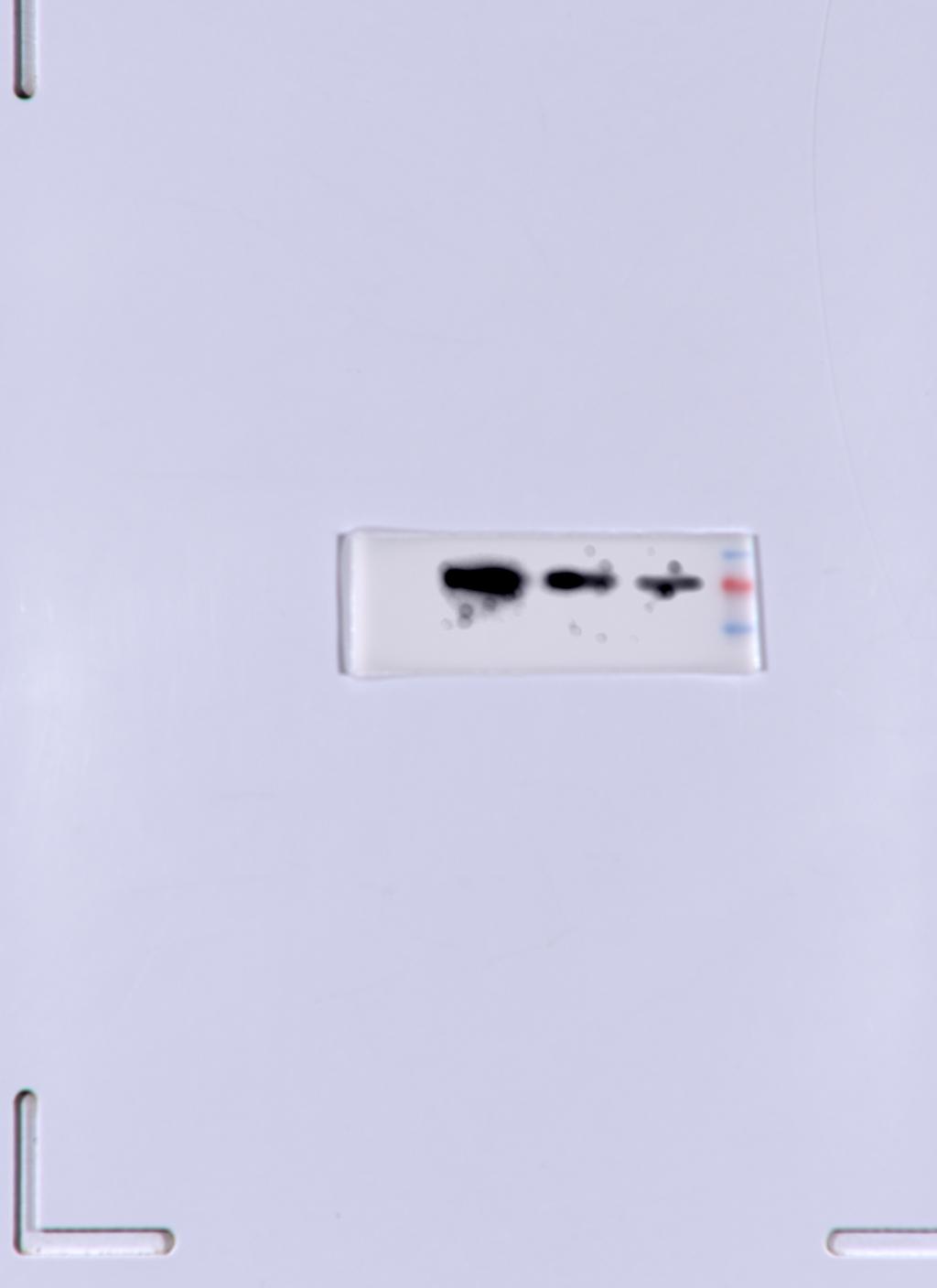
**

**GAPDH （A+B） Myc （A） Myc （B） cGAS**

**
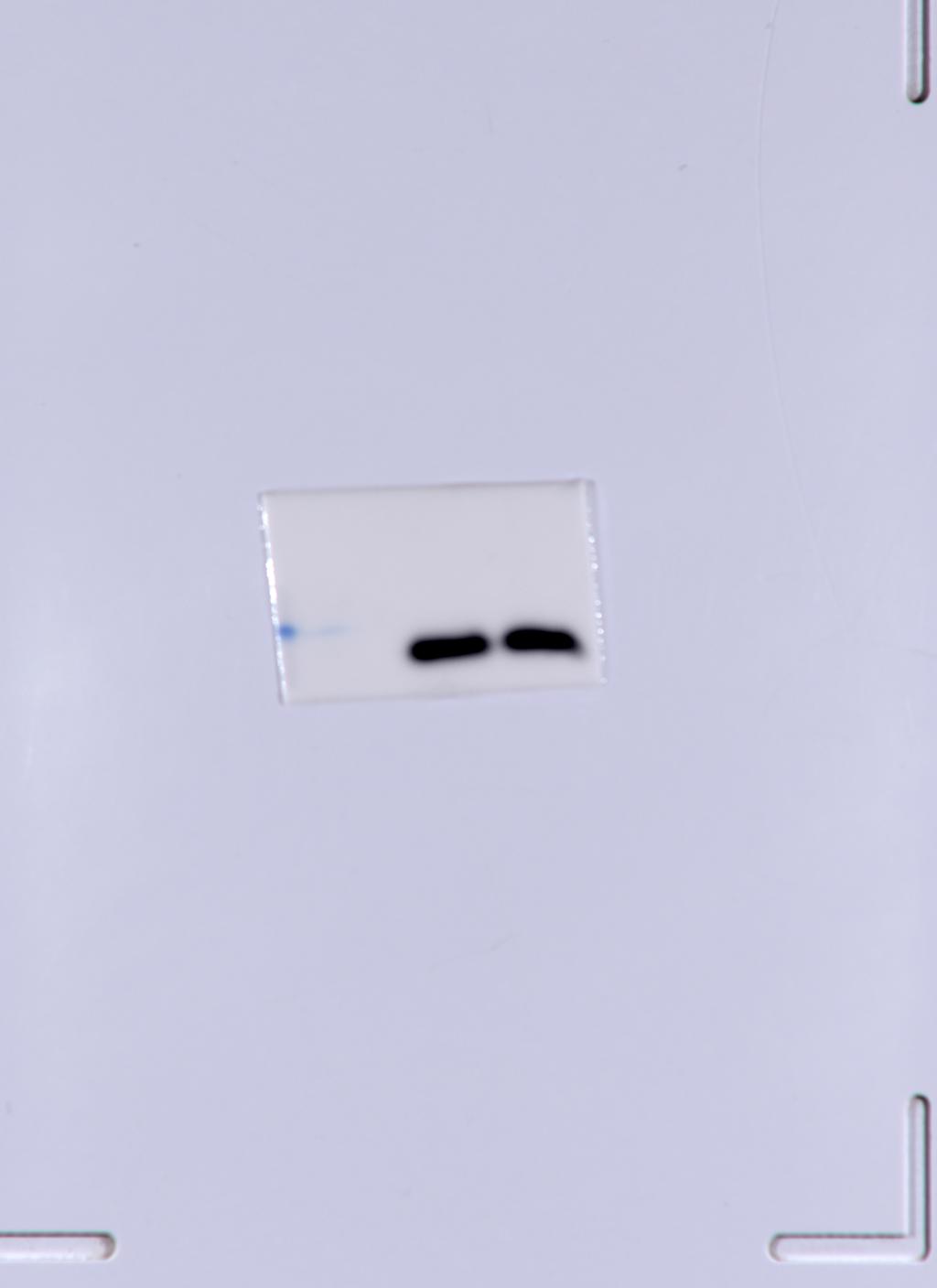
**

**HA-STING**

**C&D**

**
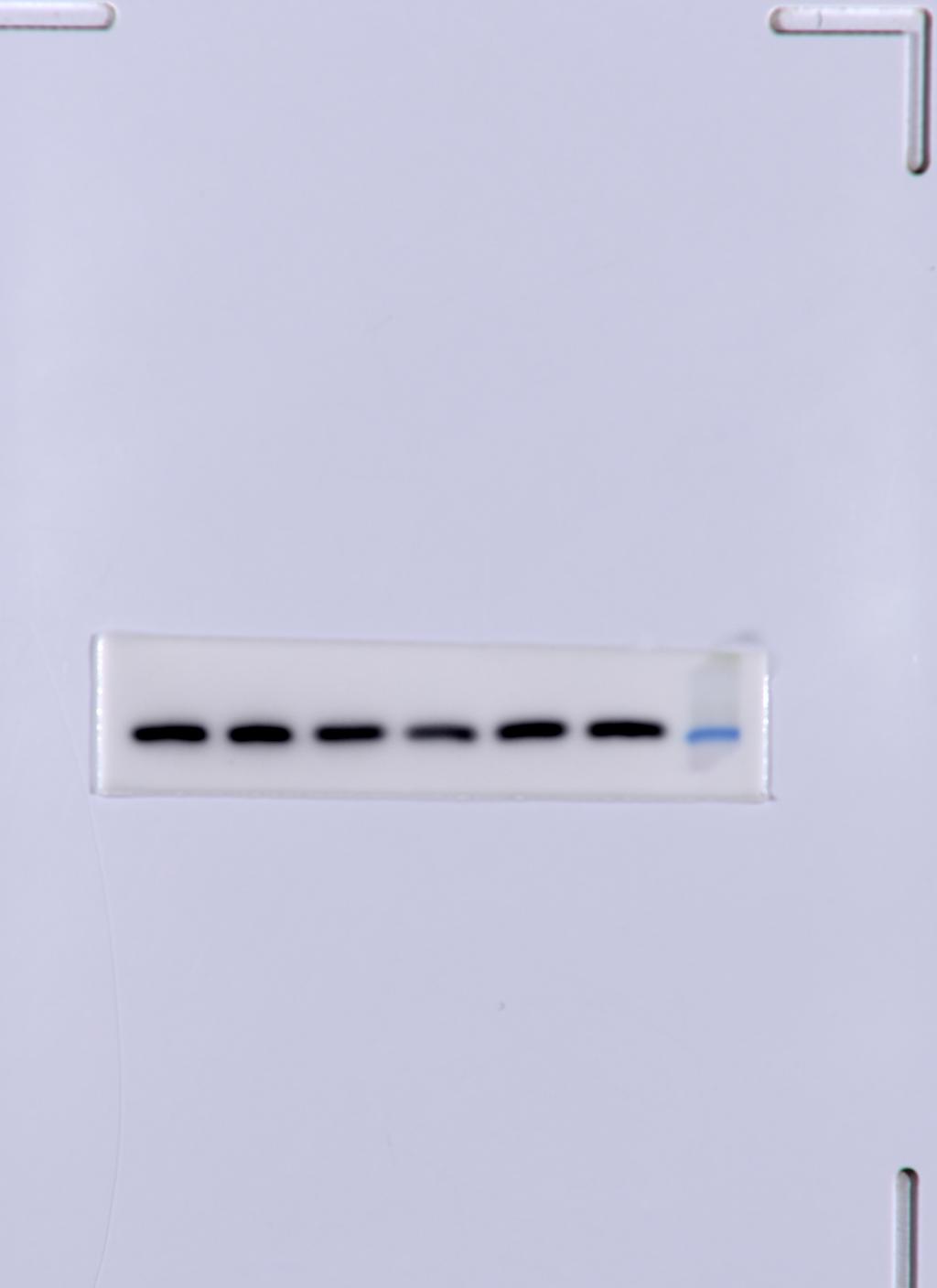

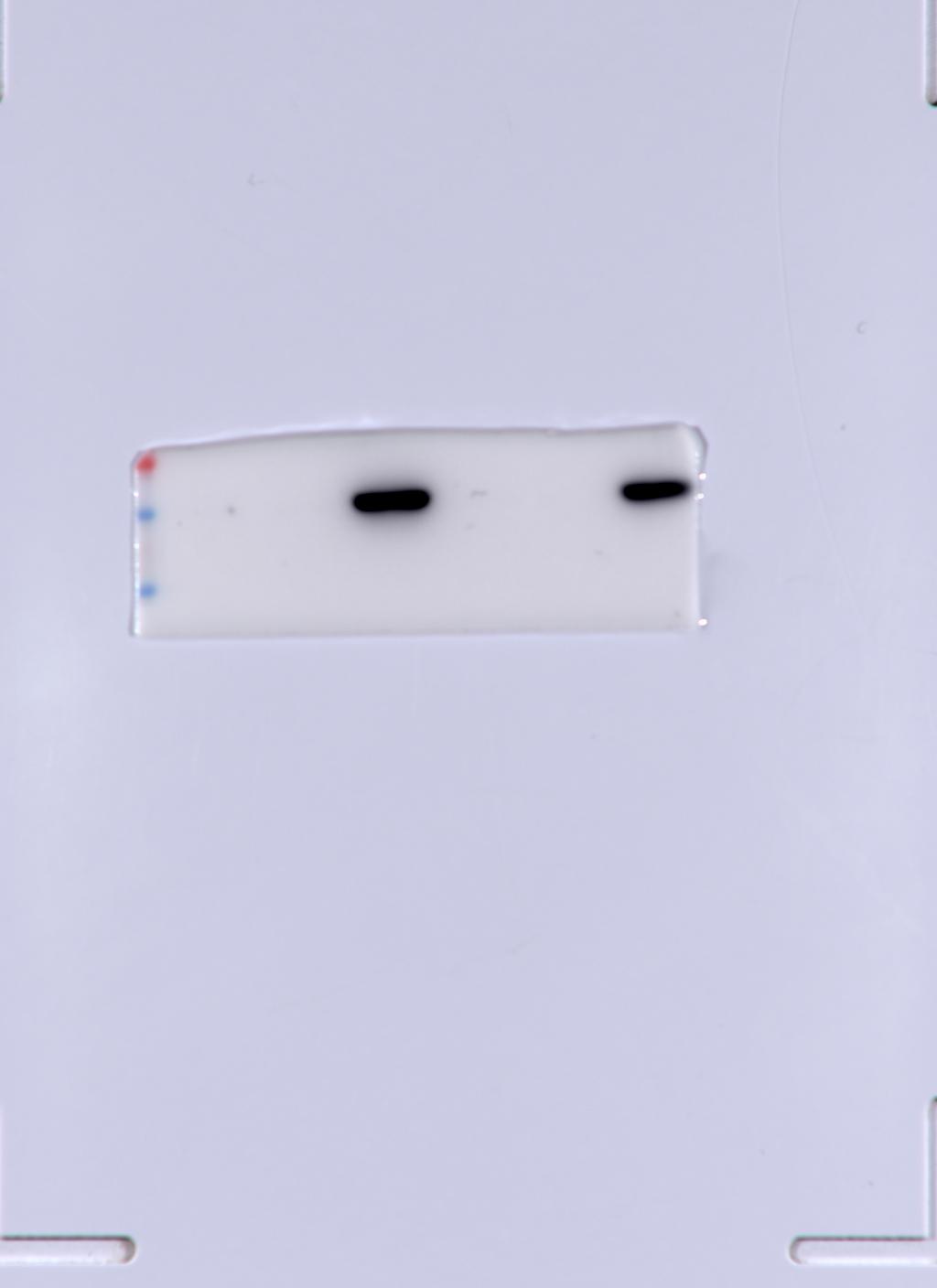

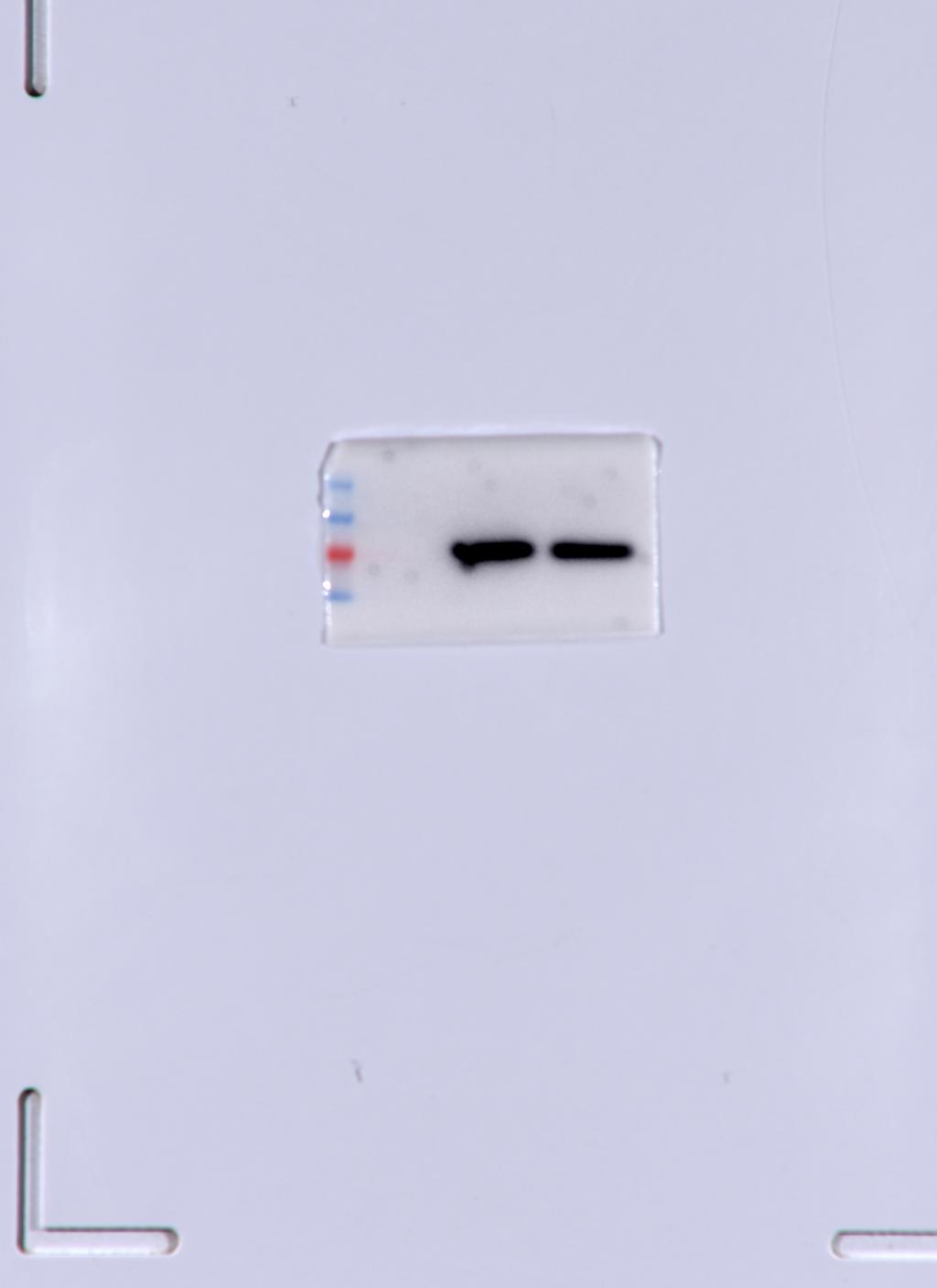

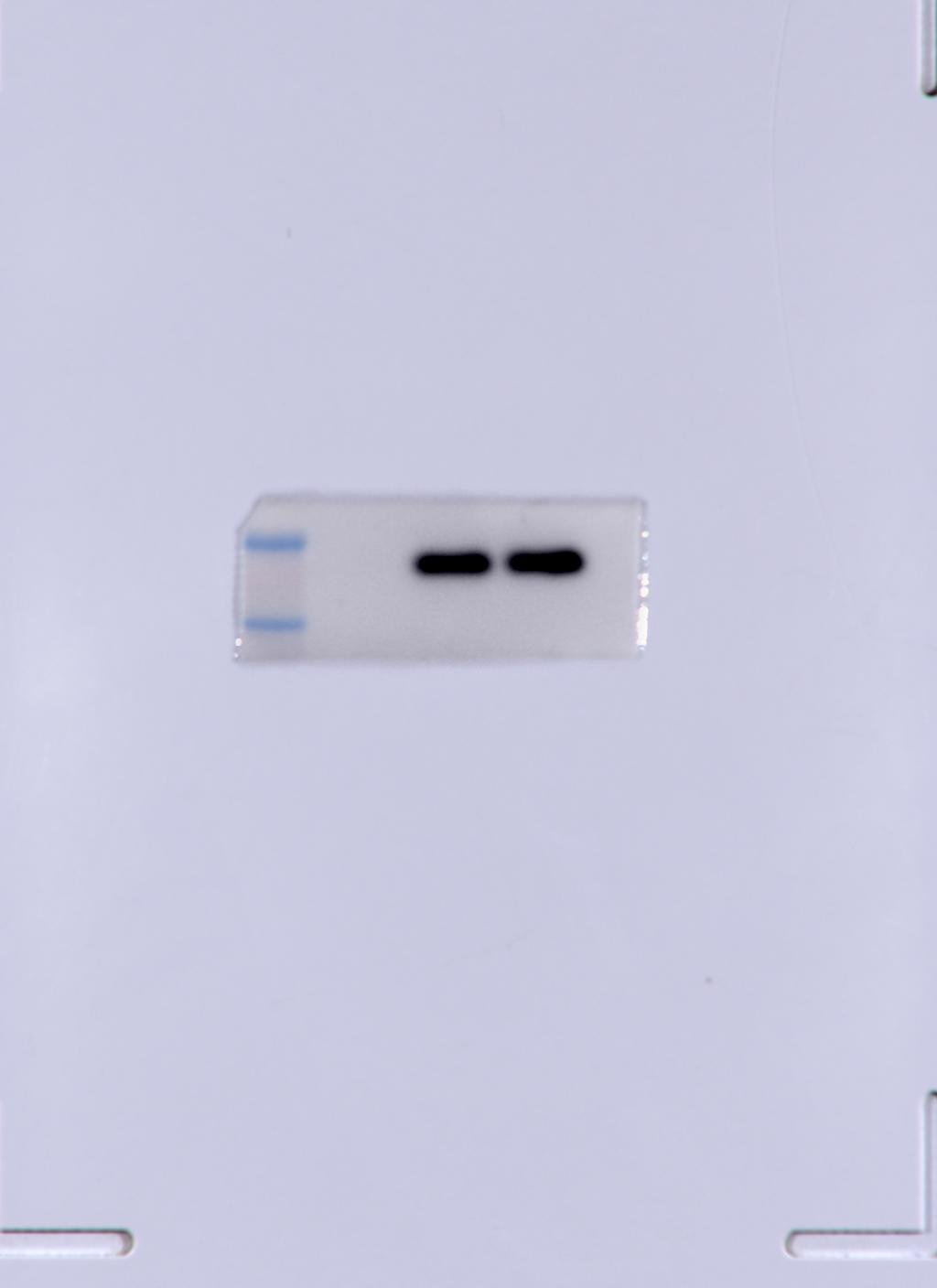
**

**GAPDH （C+D） Myc（C+D） FLAG-TBK1 FLAG- IRF3**

**5A**

| Blank | cGAS | cGAS+DDX56 |
| --- | --- | --- |
| 1 | 3.302527 | 4.788485 |
| 0.882518 | 3.220603 | 4.911586 |
| 1.133121 | 3.995996 | 5.73814 |

**5B**

| Blank | STING | STING+DDX56 |
| --- | --- | --- |
| 1 | 2.688355 | 7.233673 |
| 0.752489 | 2.978952 | 7.007511 |
| 1.328923 | 3.652062 | 8.449509 |

**5C**

| Blank | TBK1 | TBK1+DDX56 |
| --- | --- | --- |
| 1 | 4.069762 | 4.047109 |
| 0.752489 | 3.591639 | 4.696507 |
| 1.328923 | 4.611532 | 5.450109 |

**5D**

| Blank | IRF3(5D) | IRF3(5D)+DDX56 |
| --- | --- | --- |
| 1 | 2.980609 | 3.476549 |
| 0.752489 | 2.525434 | 3.873624 |
| 1.328923 | 3.517822 | 3.134874 |

**Figure 6**

**A**

**
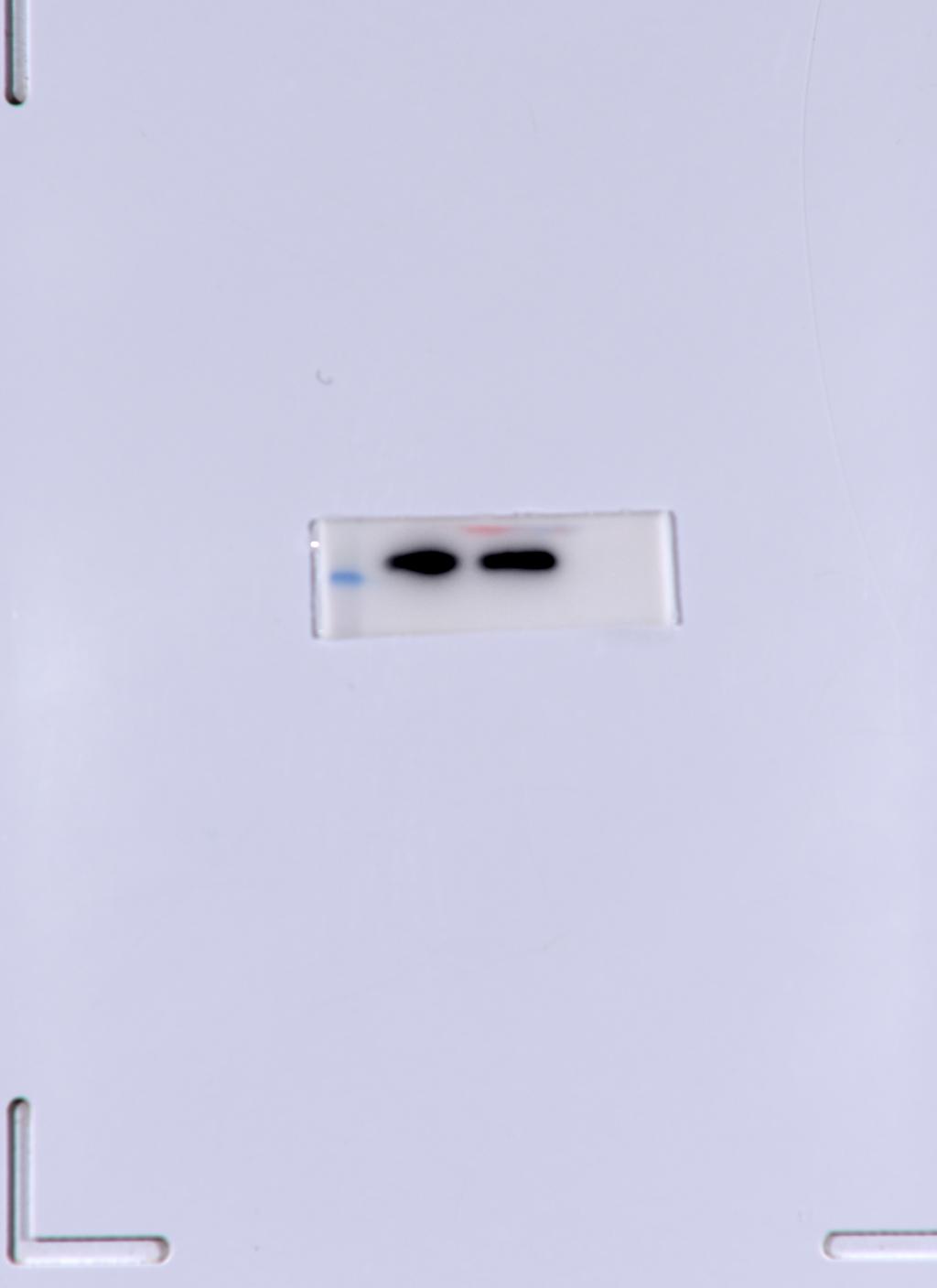

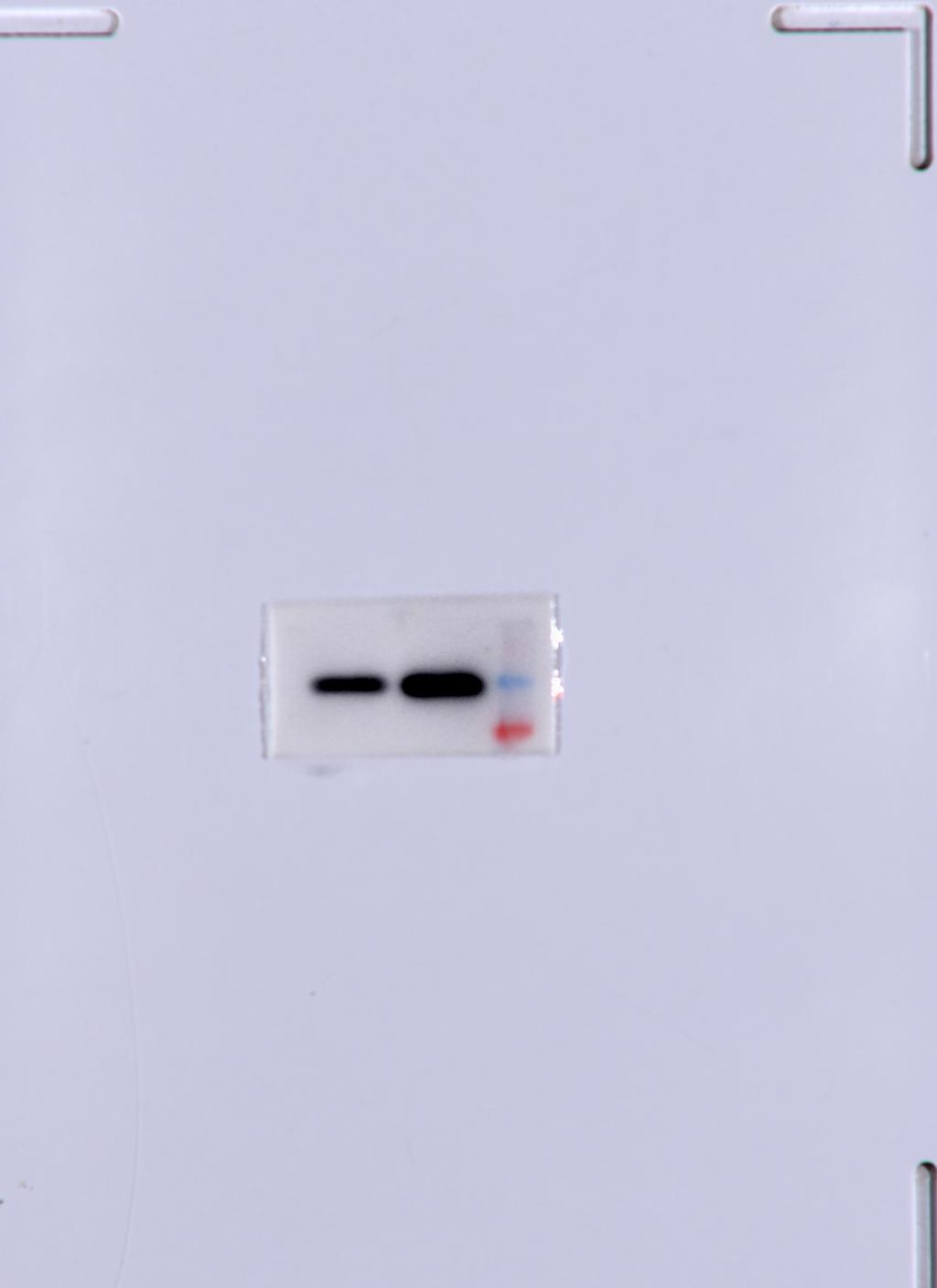

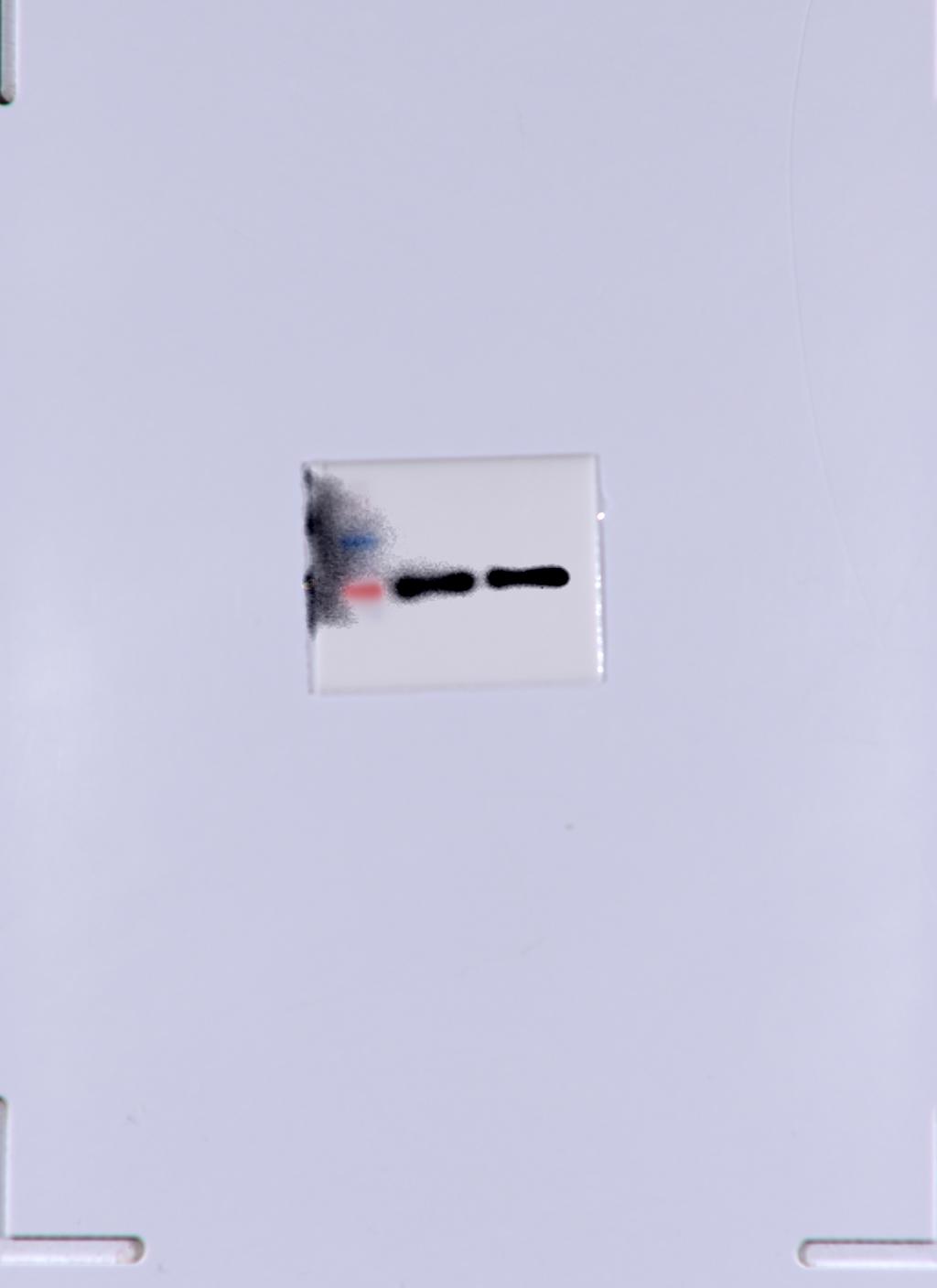
**

**Input-GAPDH Input-cGAS Input-Myc**

**
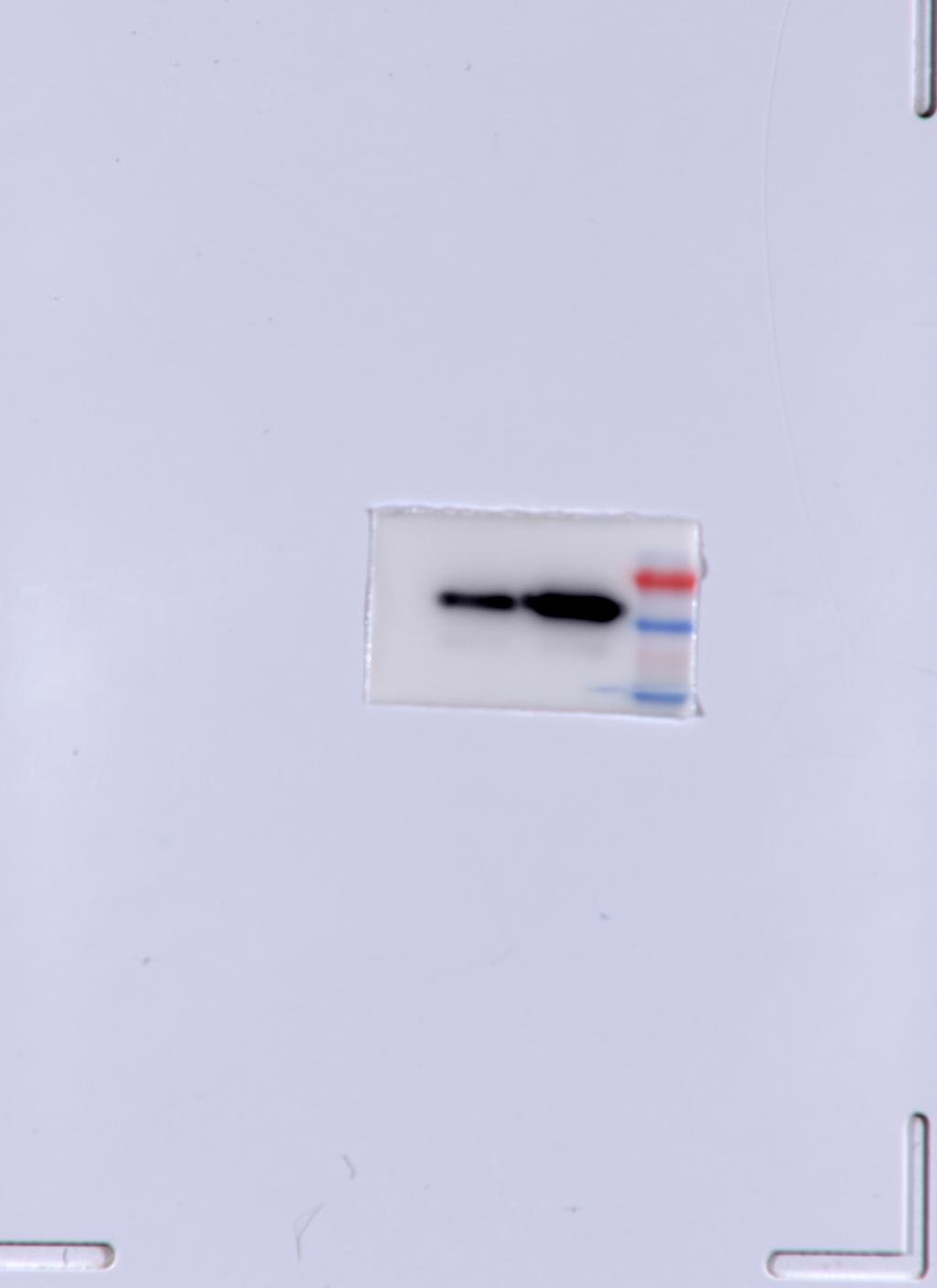

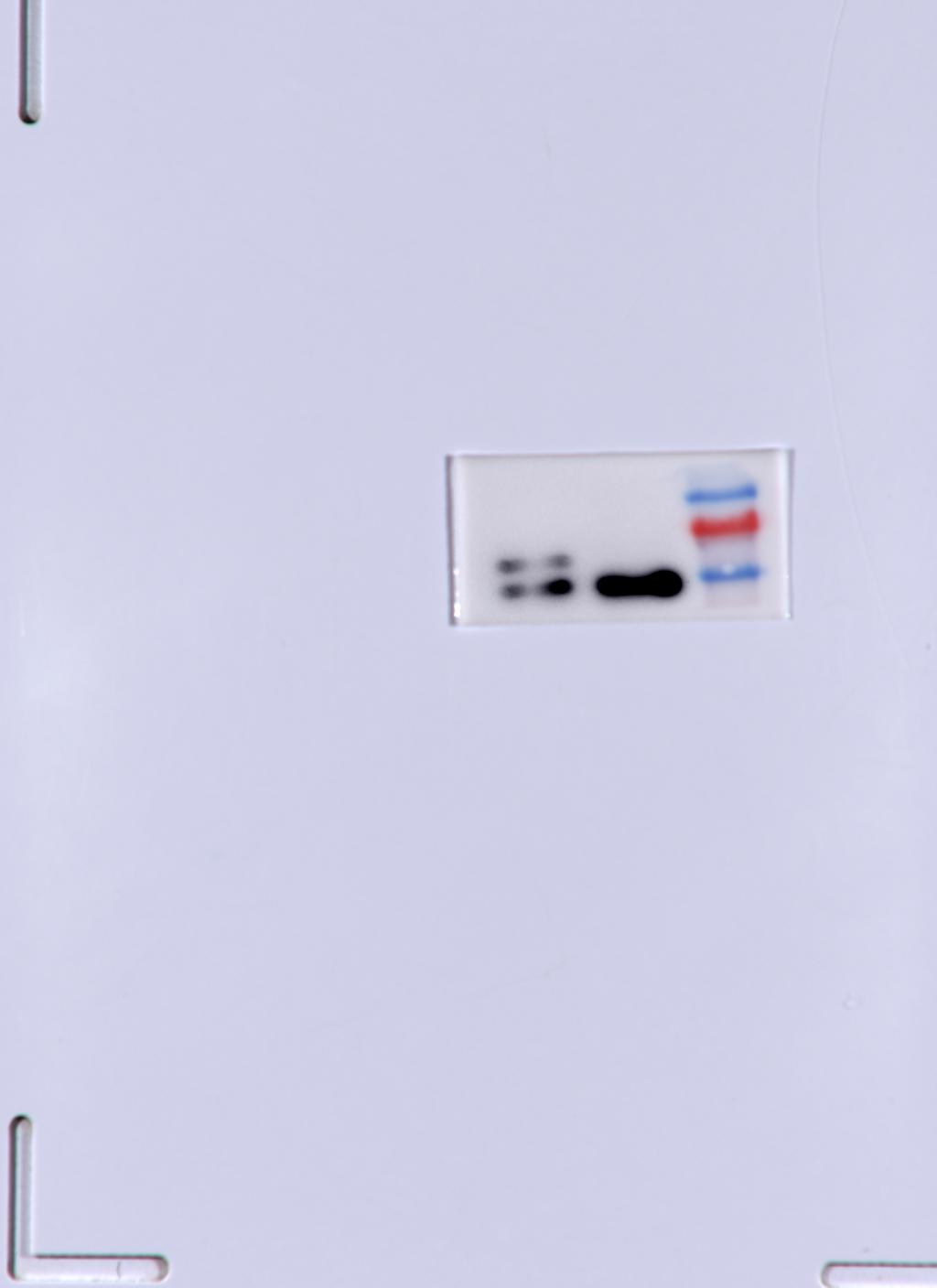
**

**IP-Myc IP-cGAS**

**B**

**
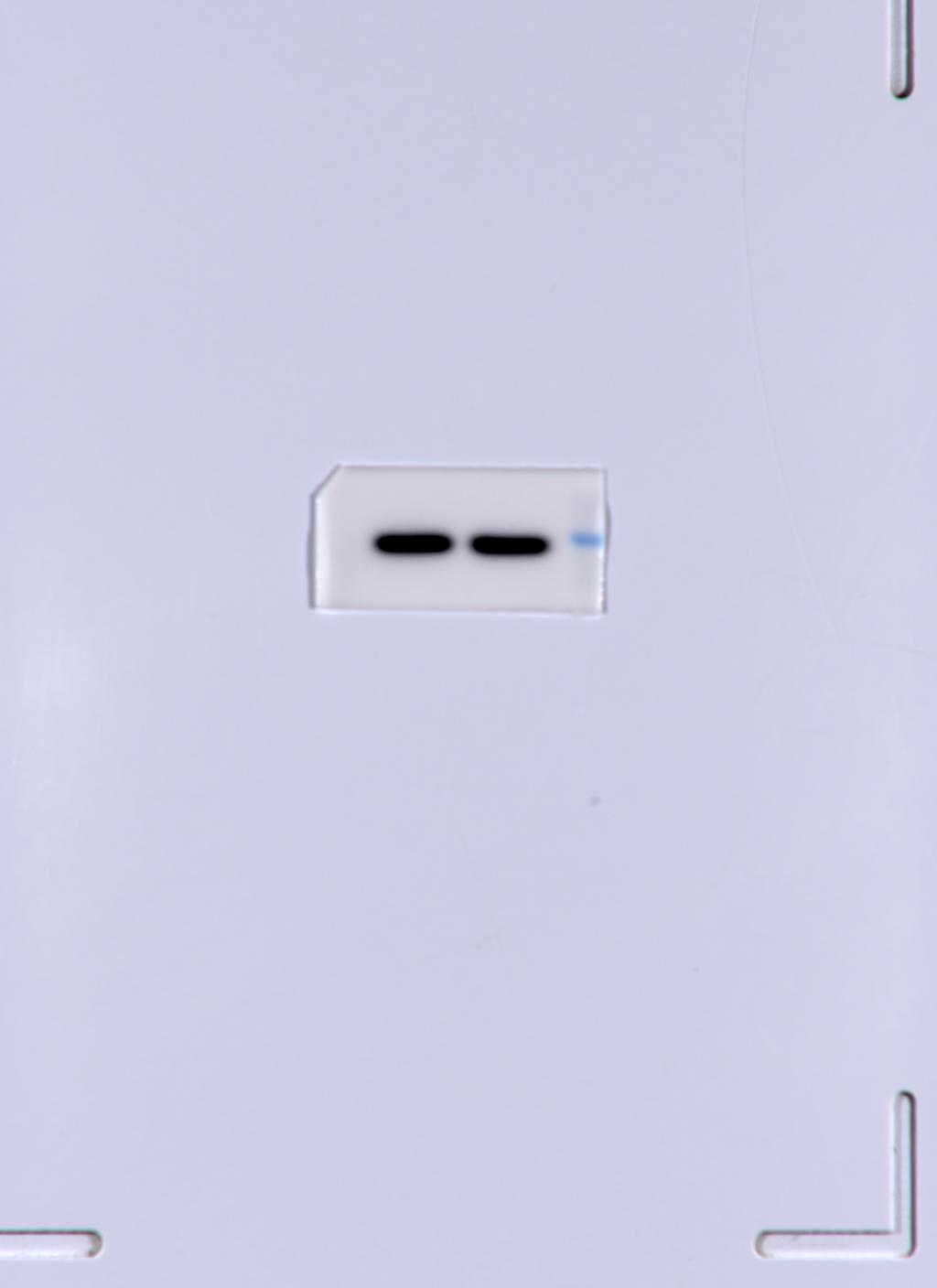

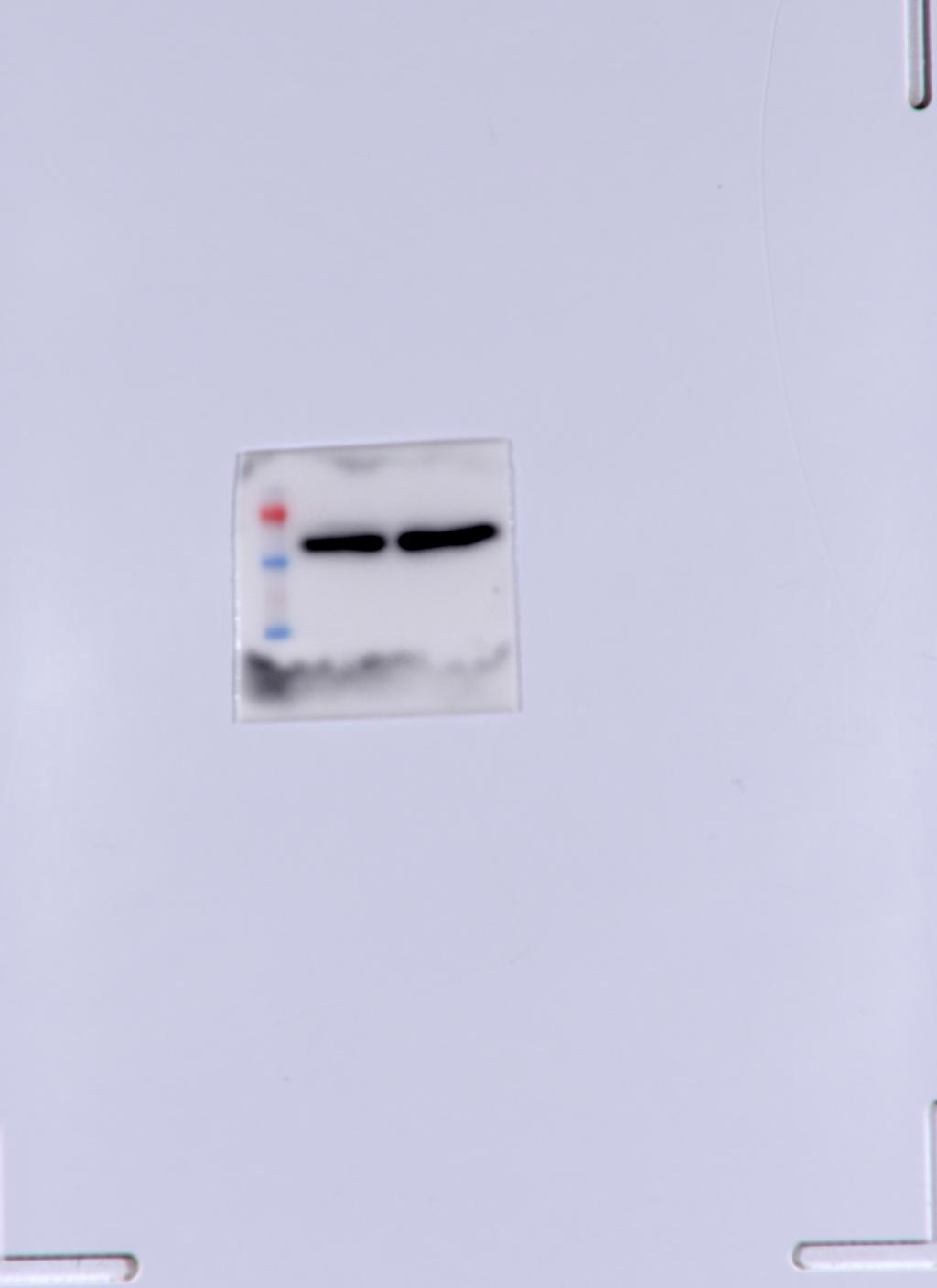

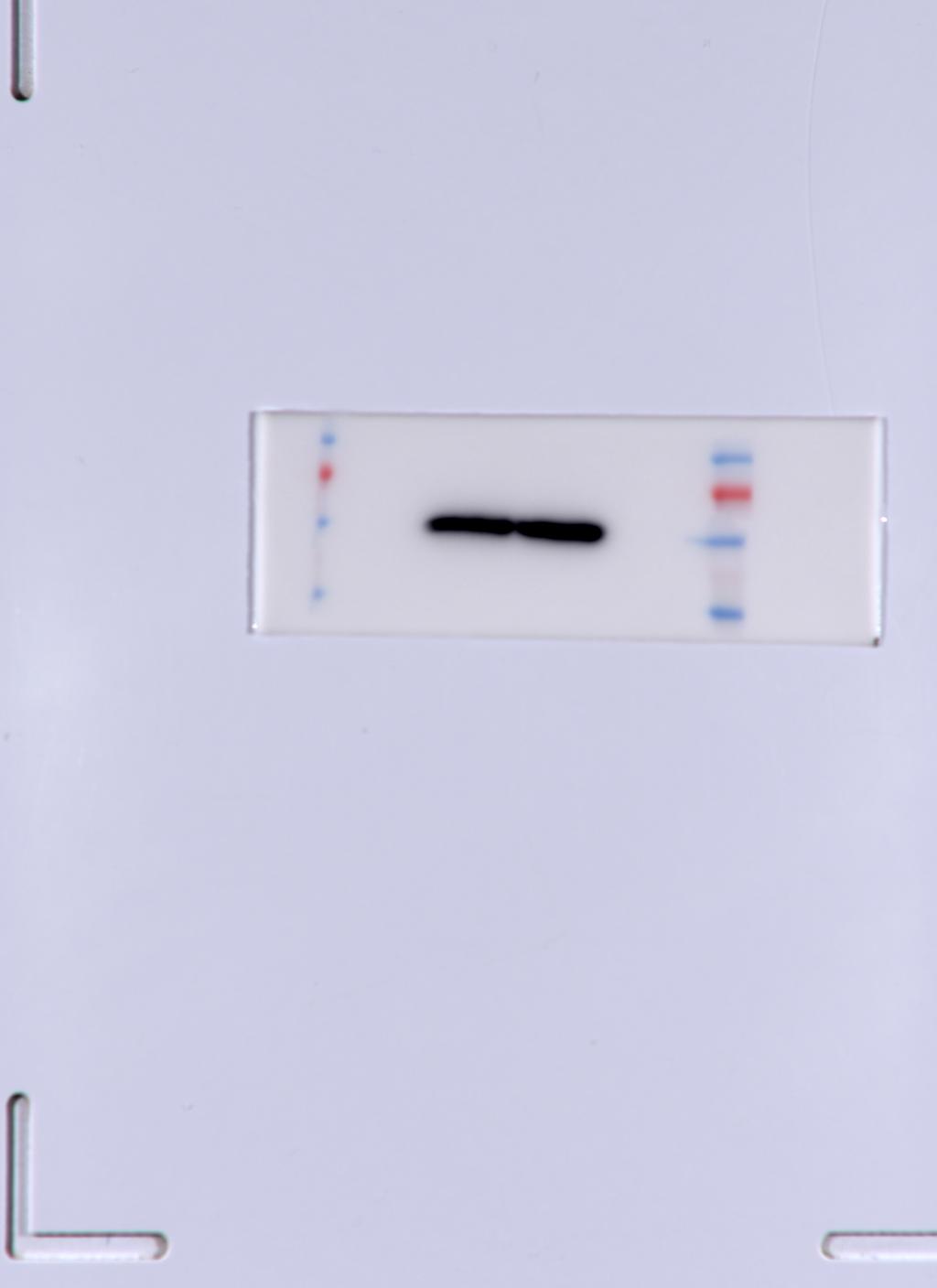
**

**Input-GAPDH Input-cGAS Input-Myc**

**
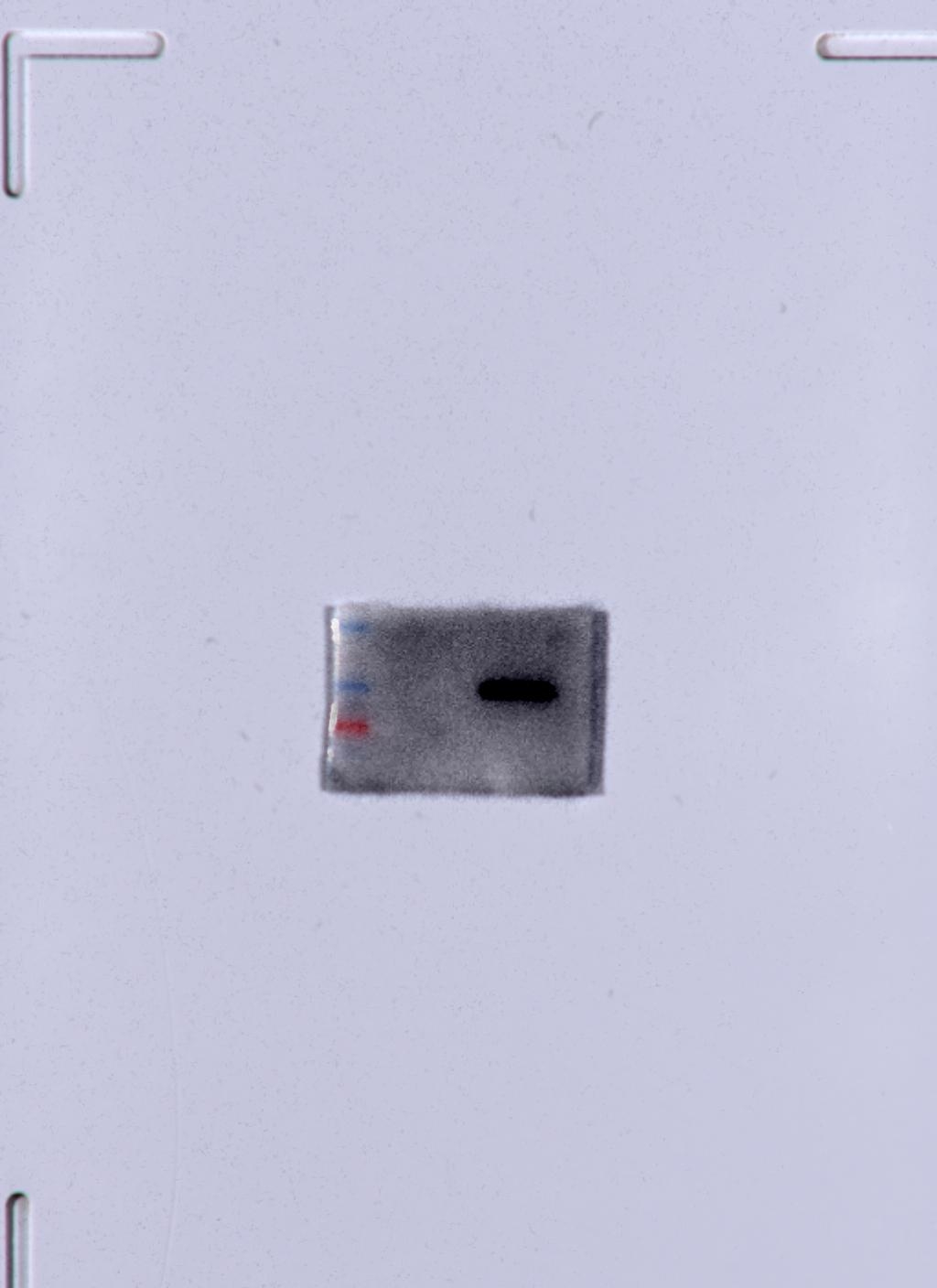

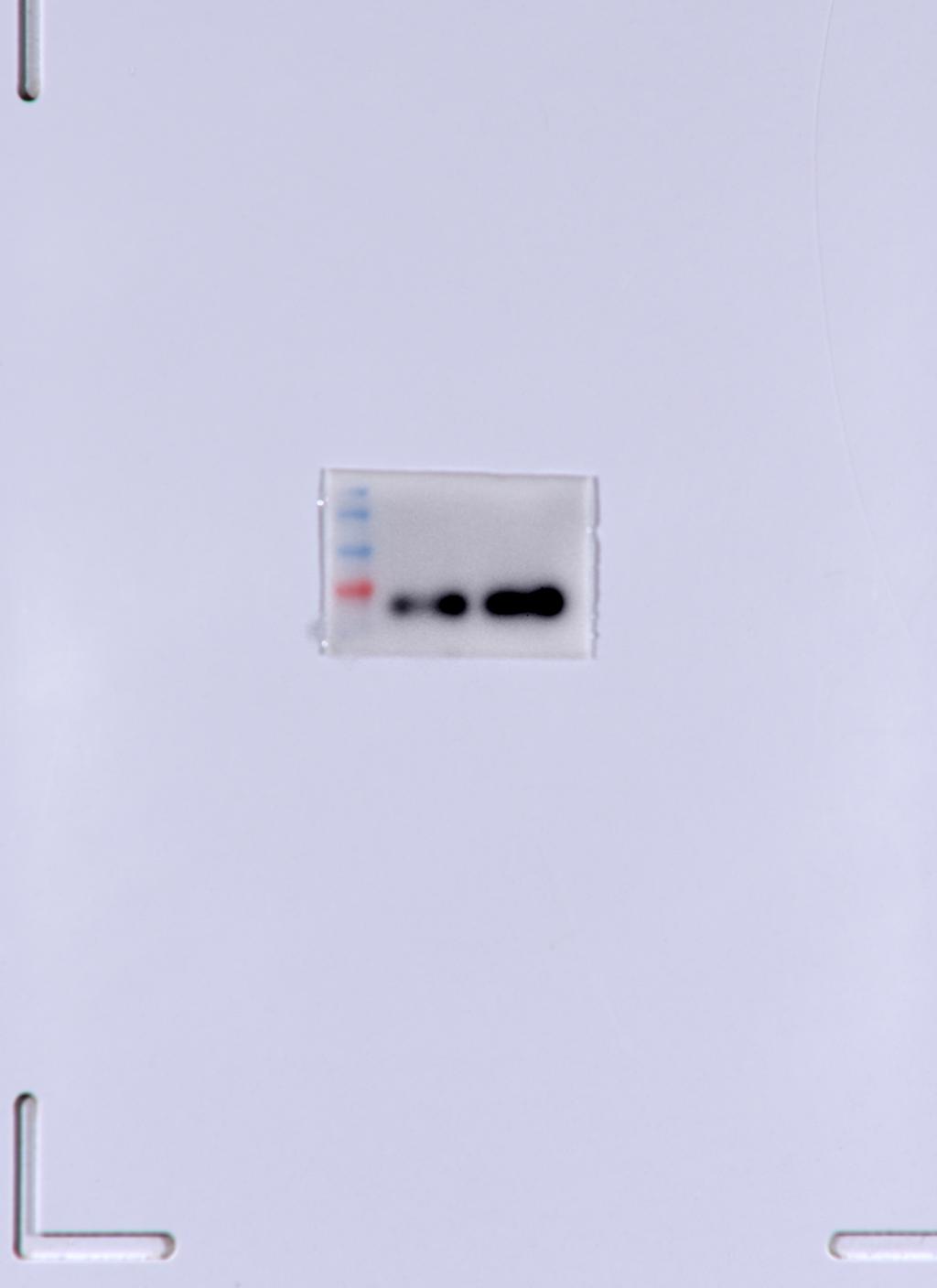
**

**IP-Myc IP-cGAS**

**C**

**
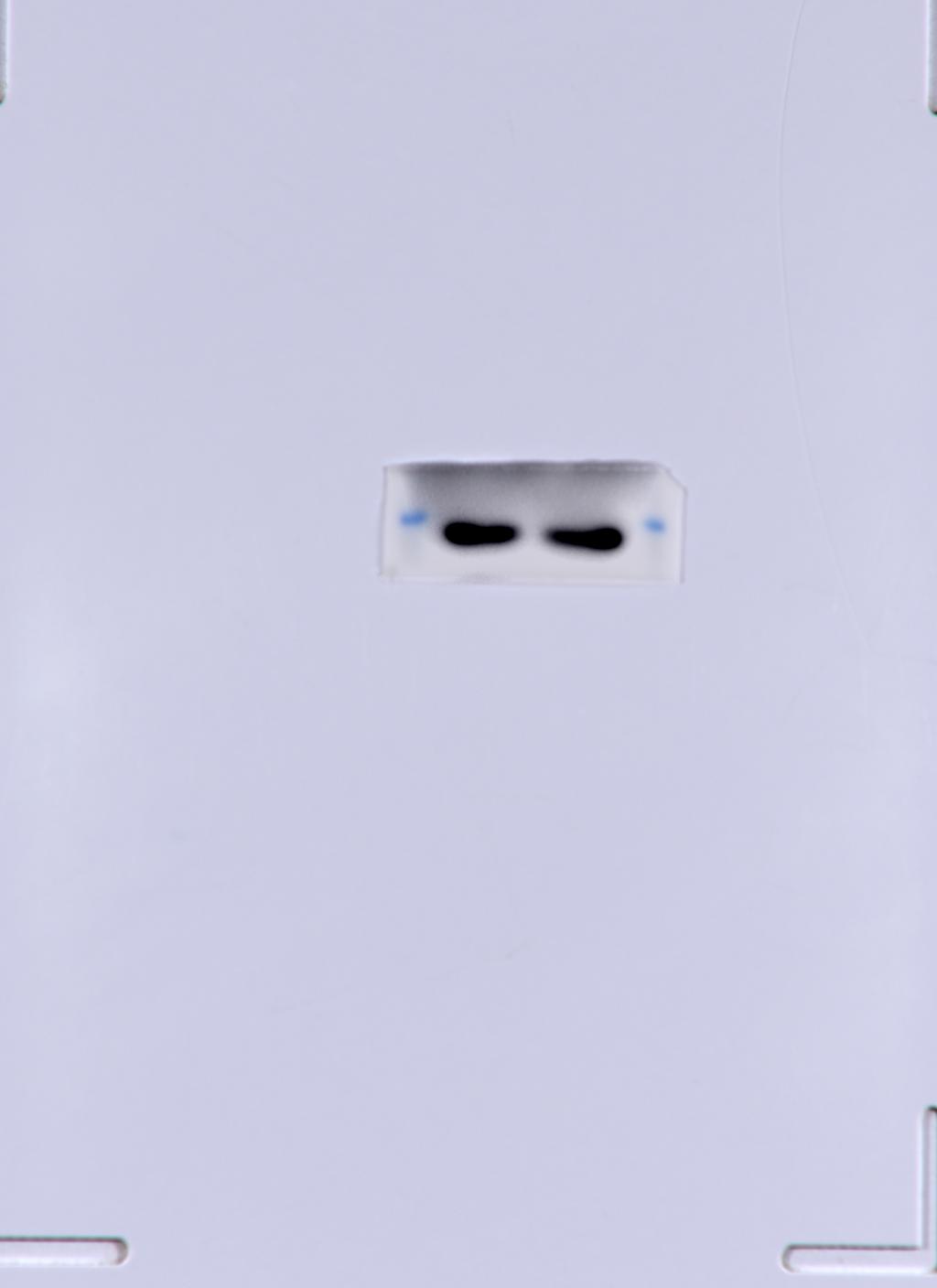

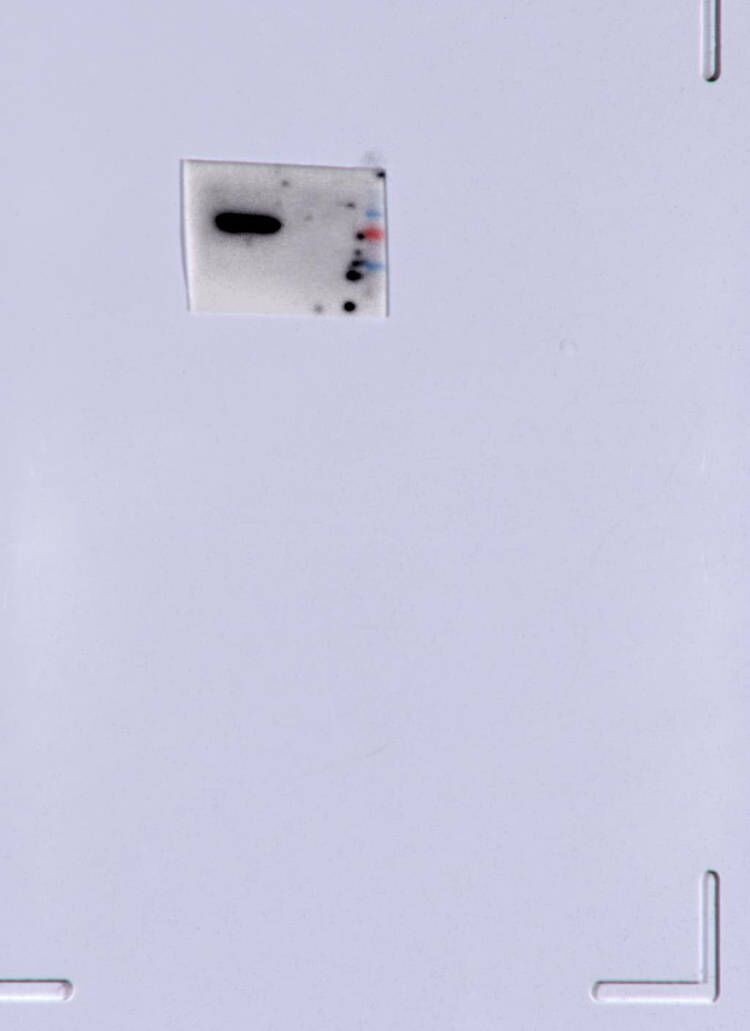

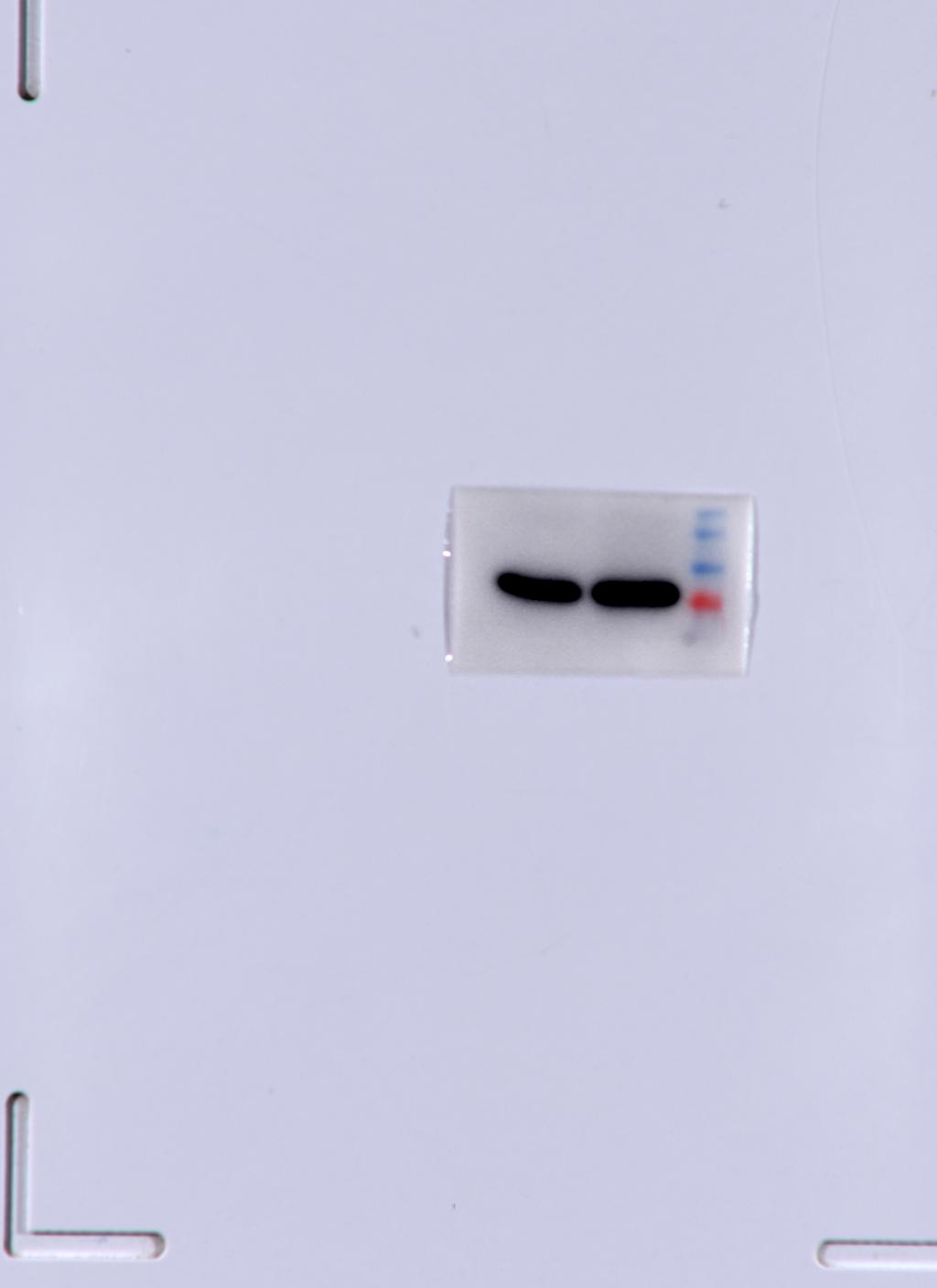
**

**Input-GAPDH Input-FLAG Input-Myc**

**
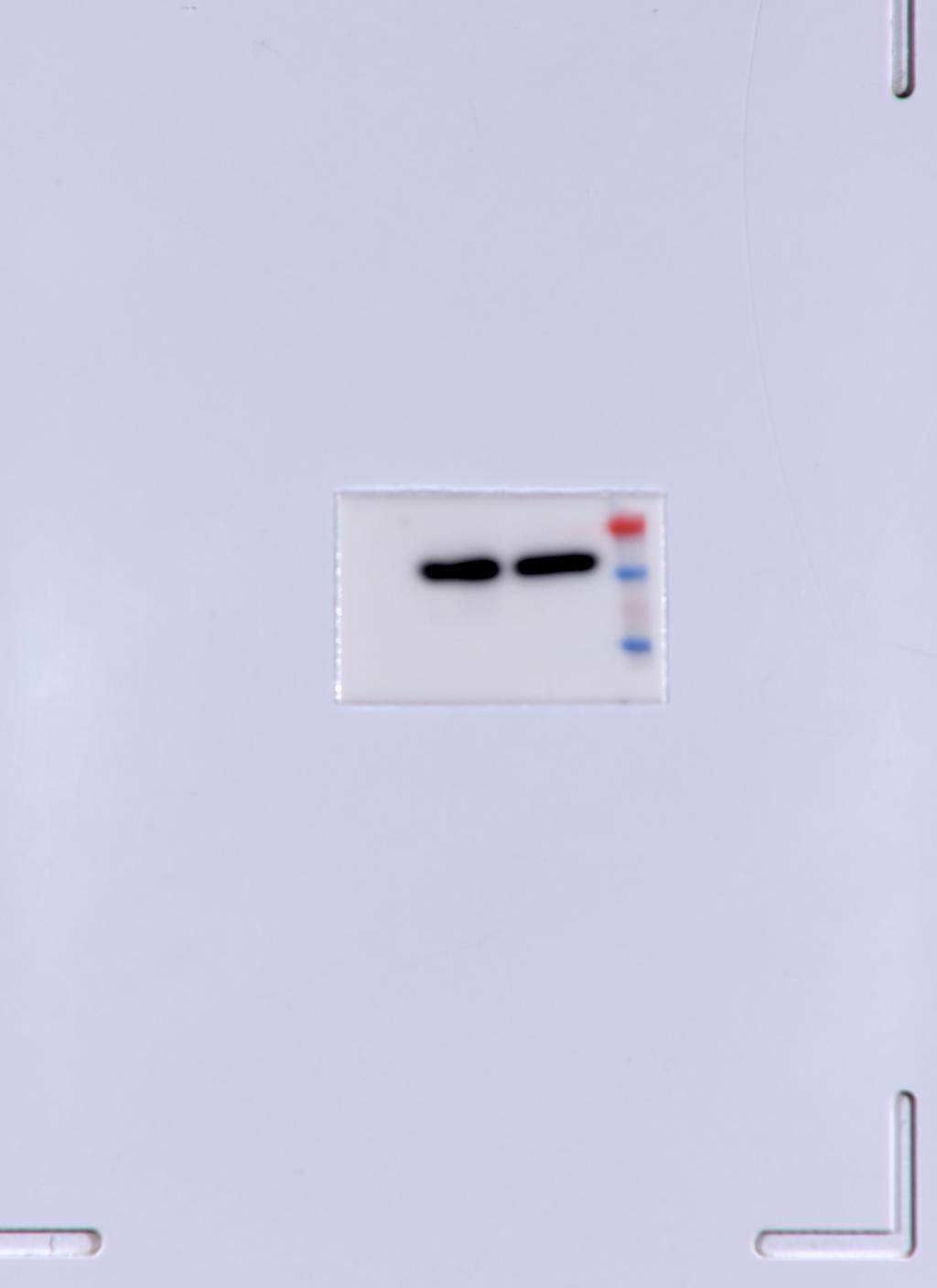

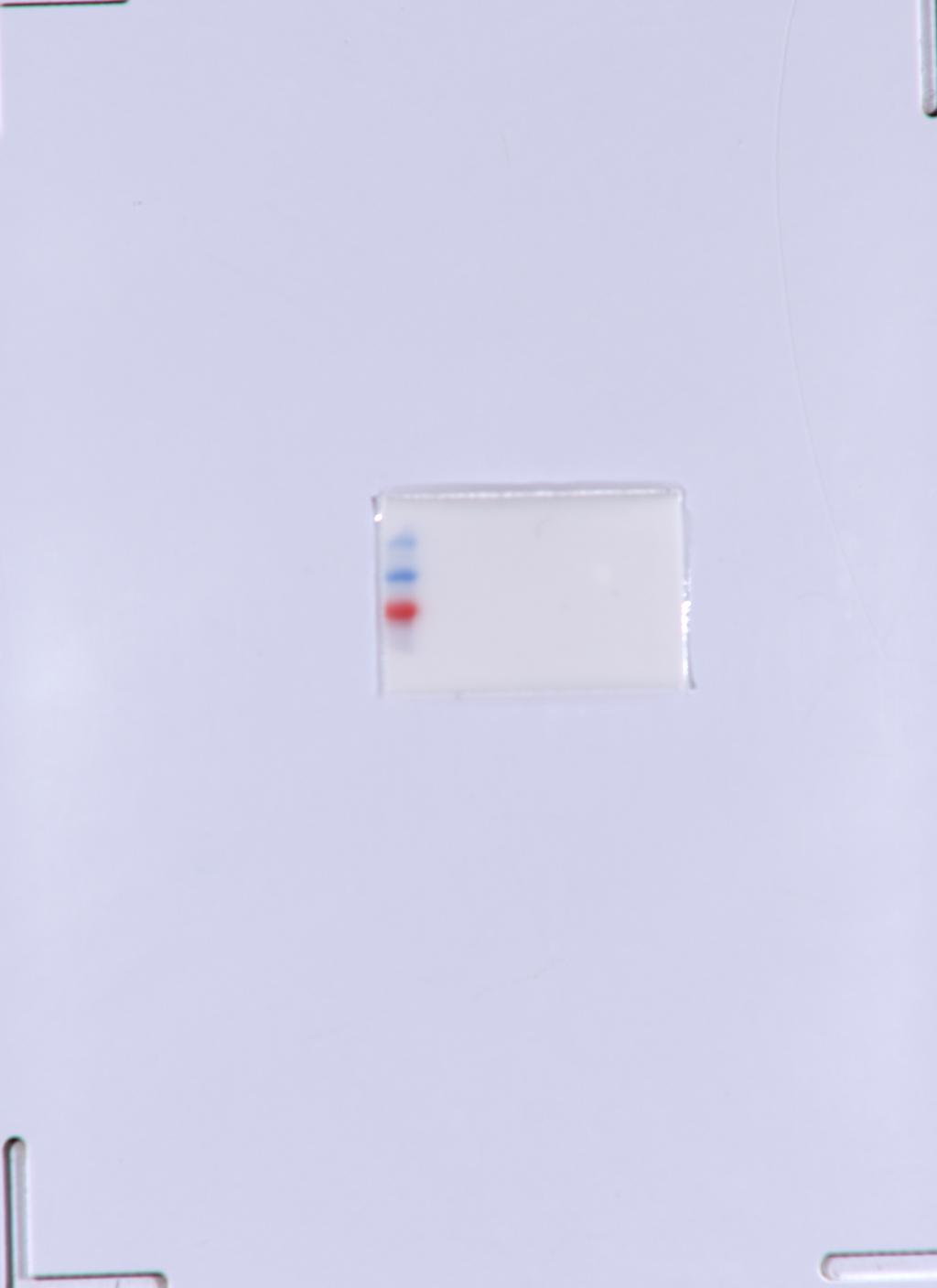
**

**IP-Myc IP-FLAG**

**D**

**
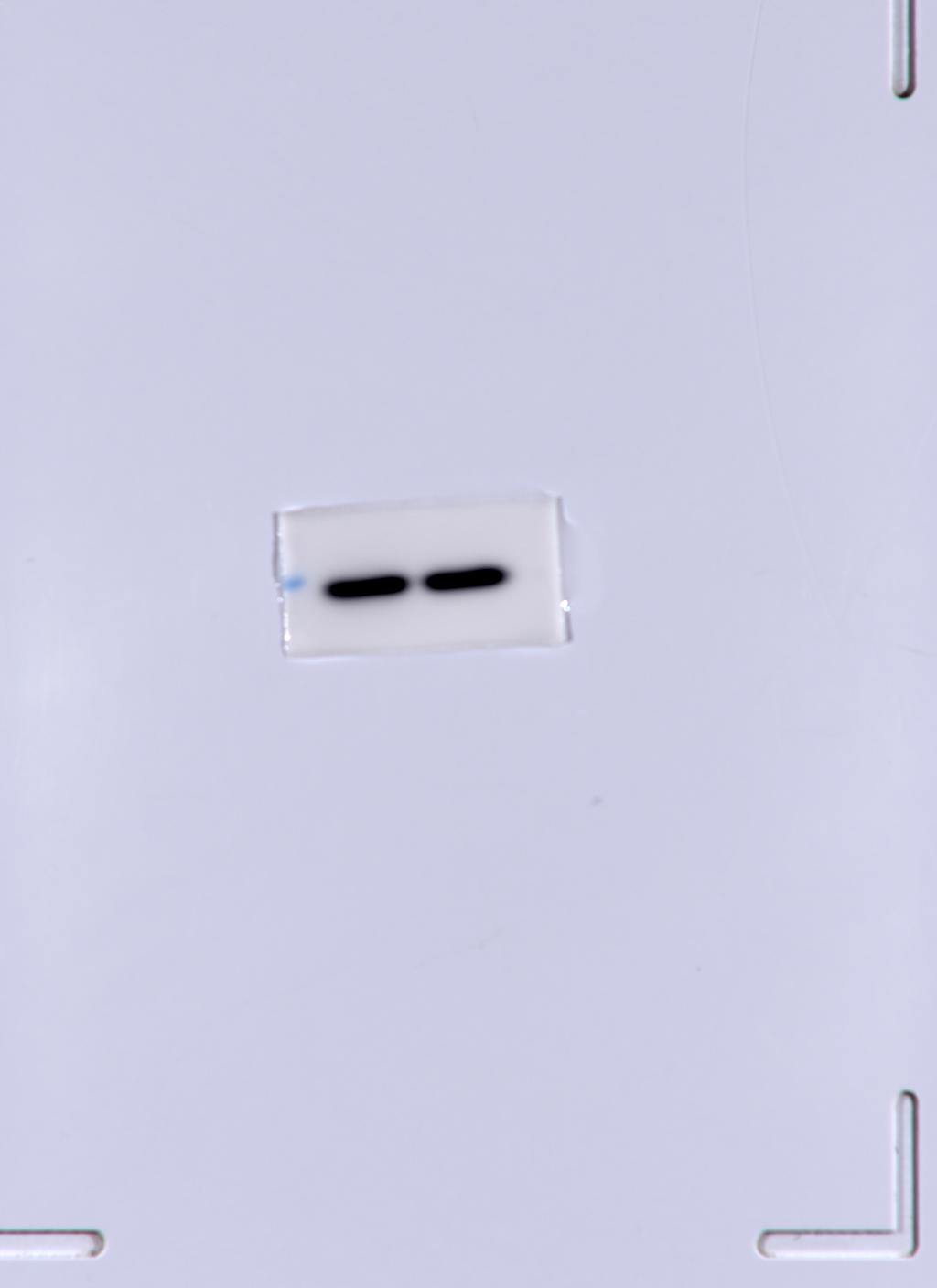

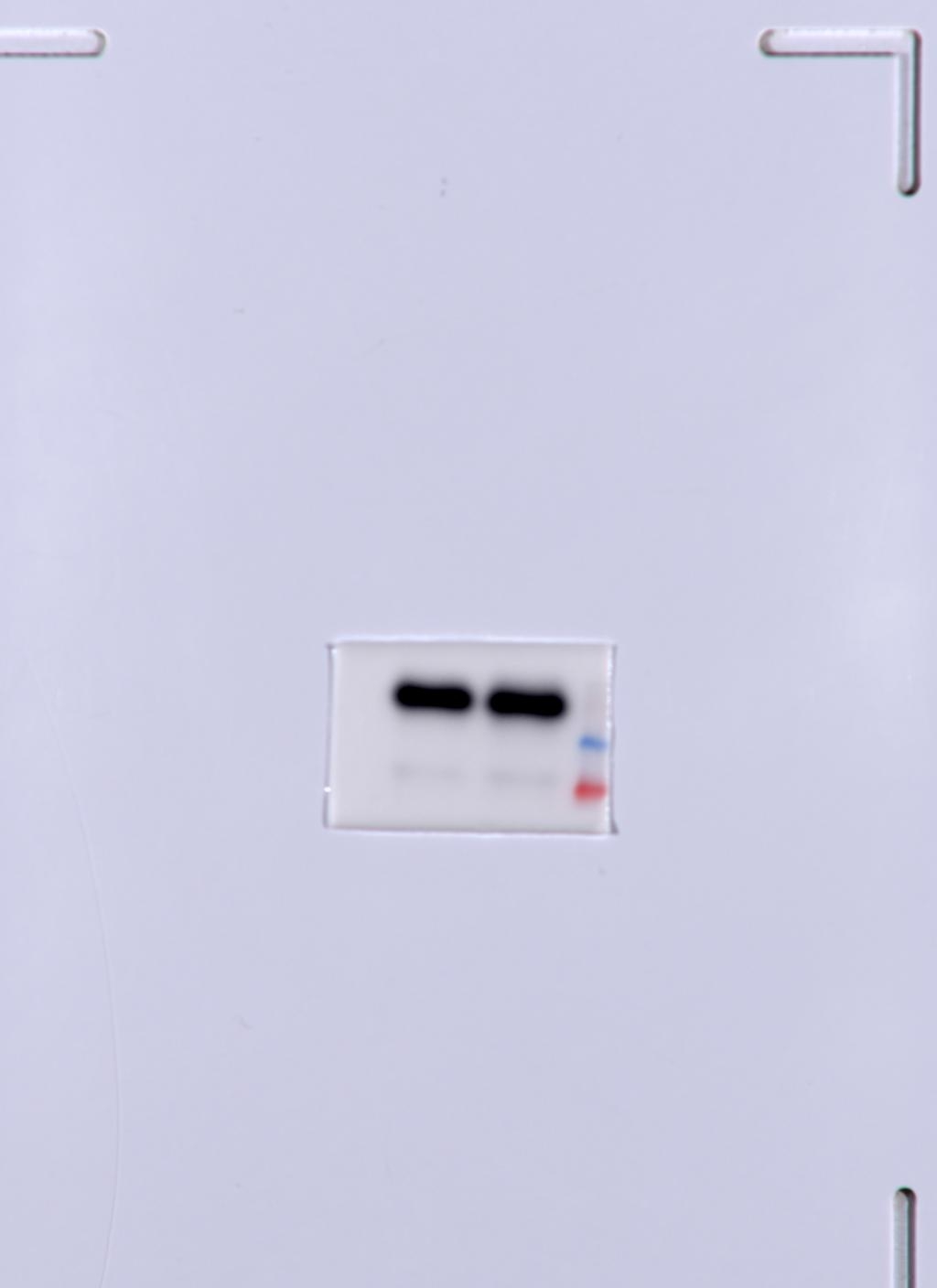

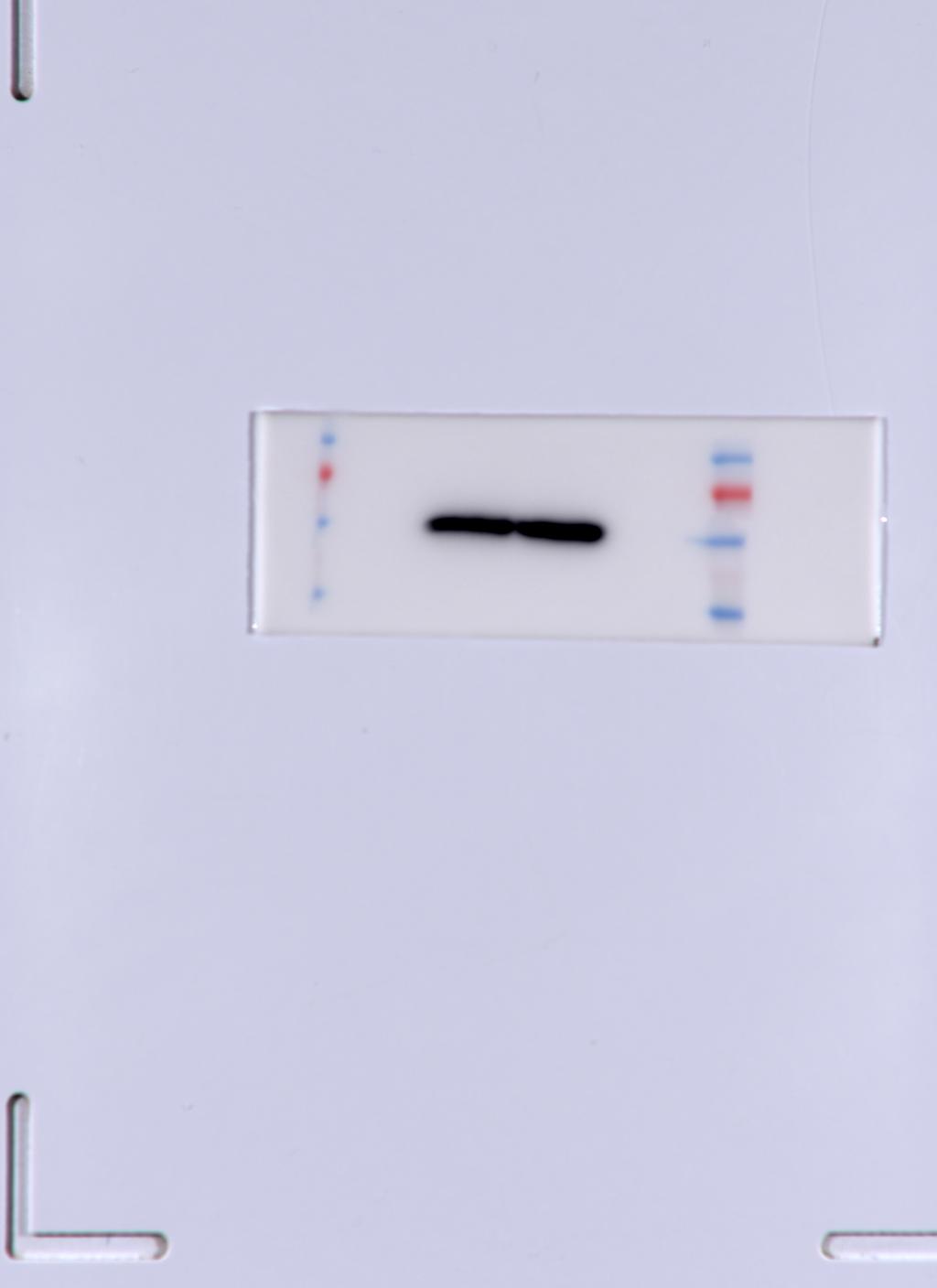
**

**Input-GAPDH Input-FLAG Input-Myc**

**
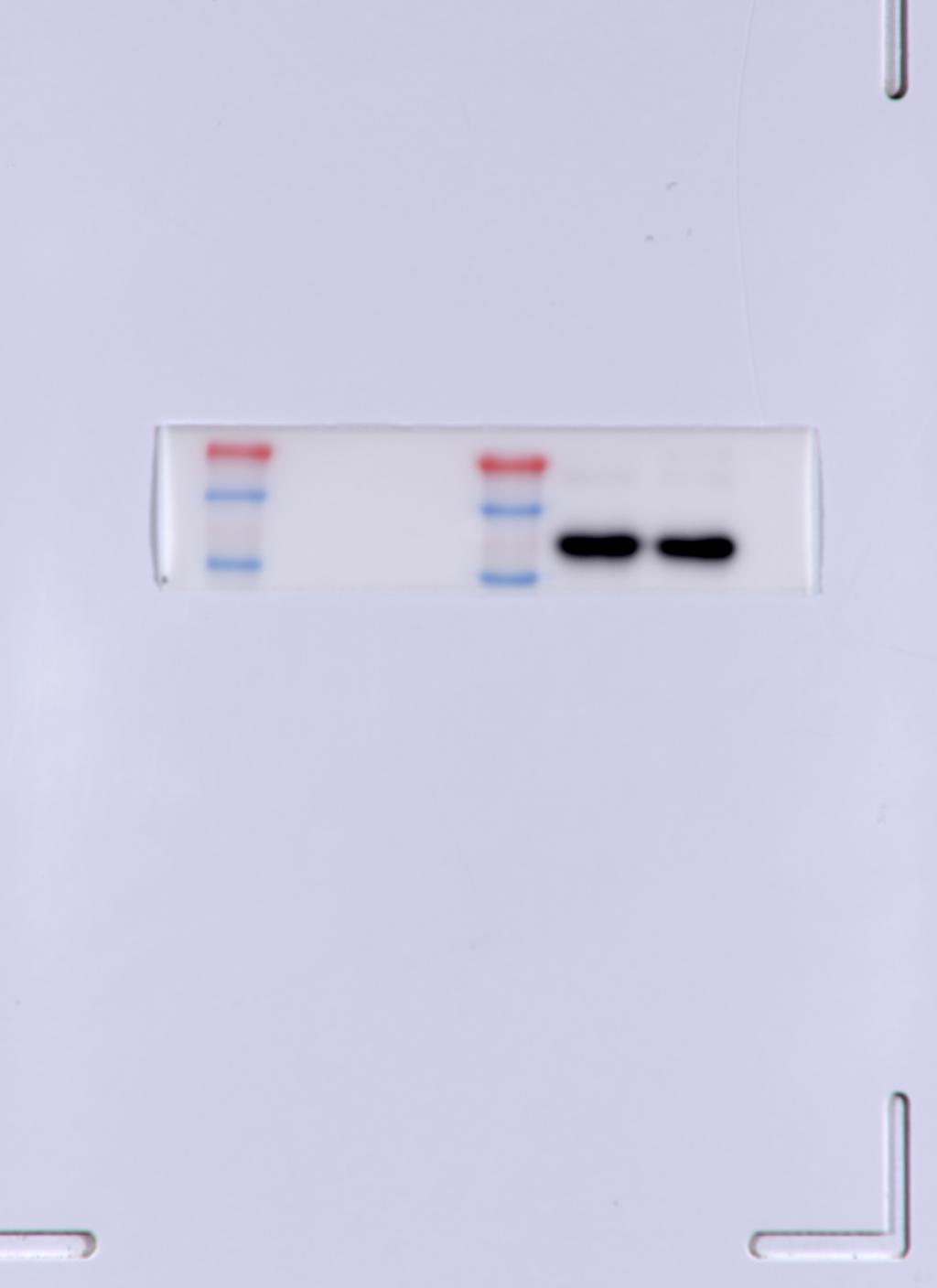

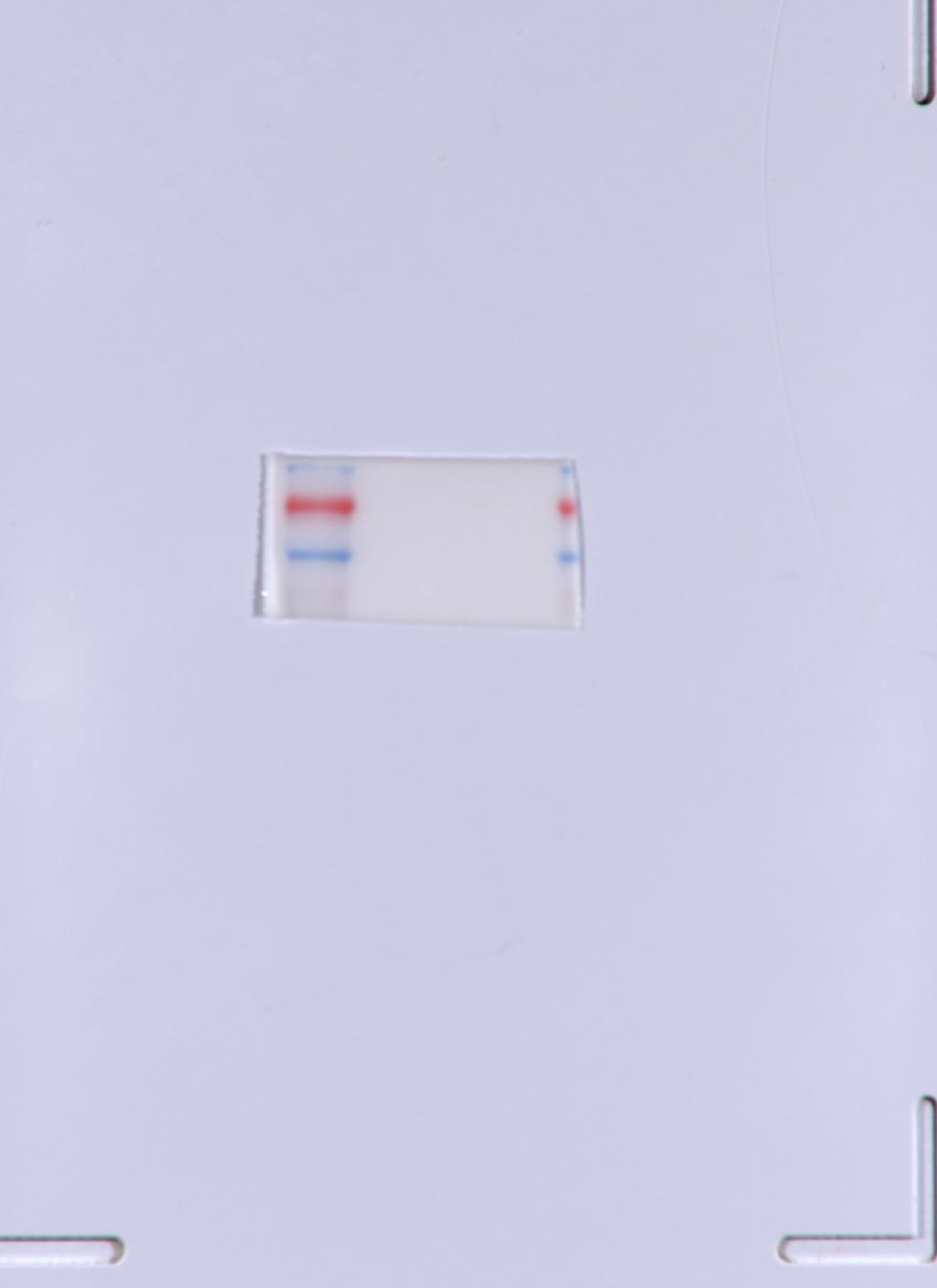
**

**IP-FLAG IP-Myc**

**Figure 7**

**A**

**
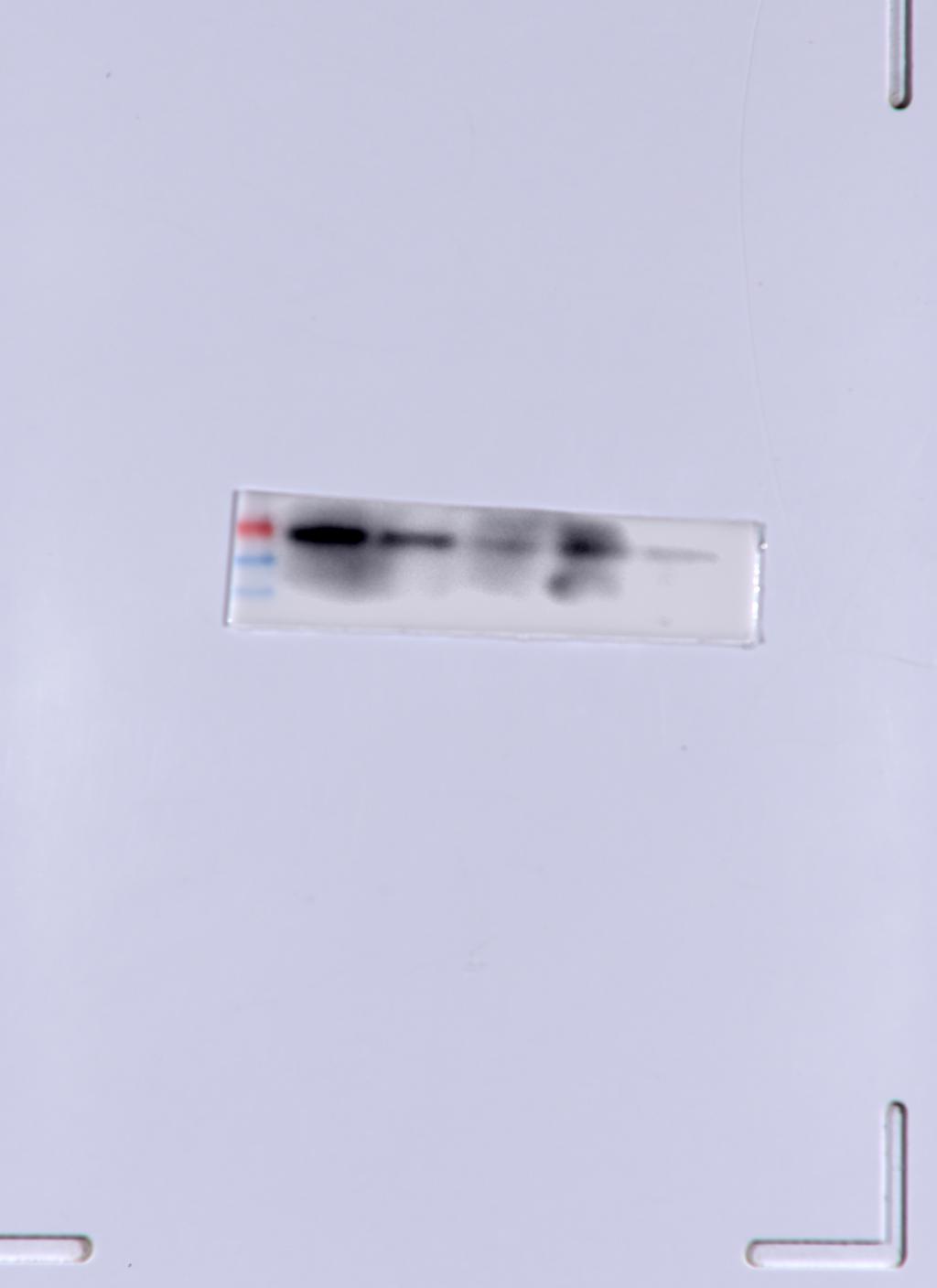

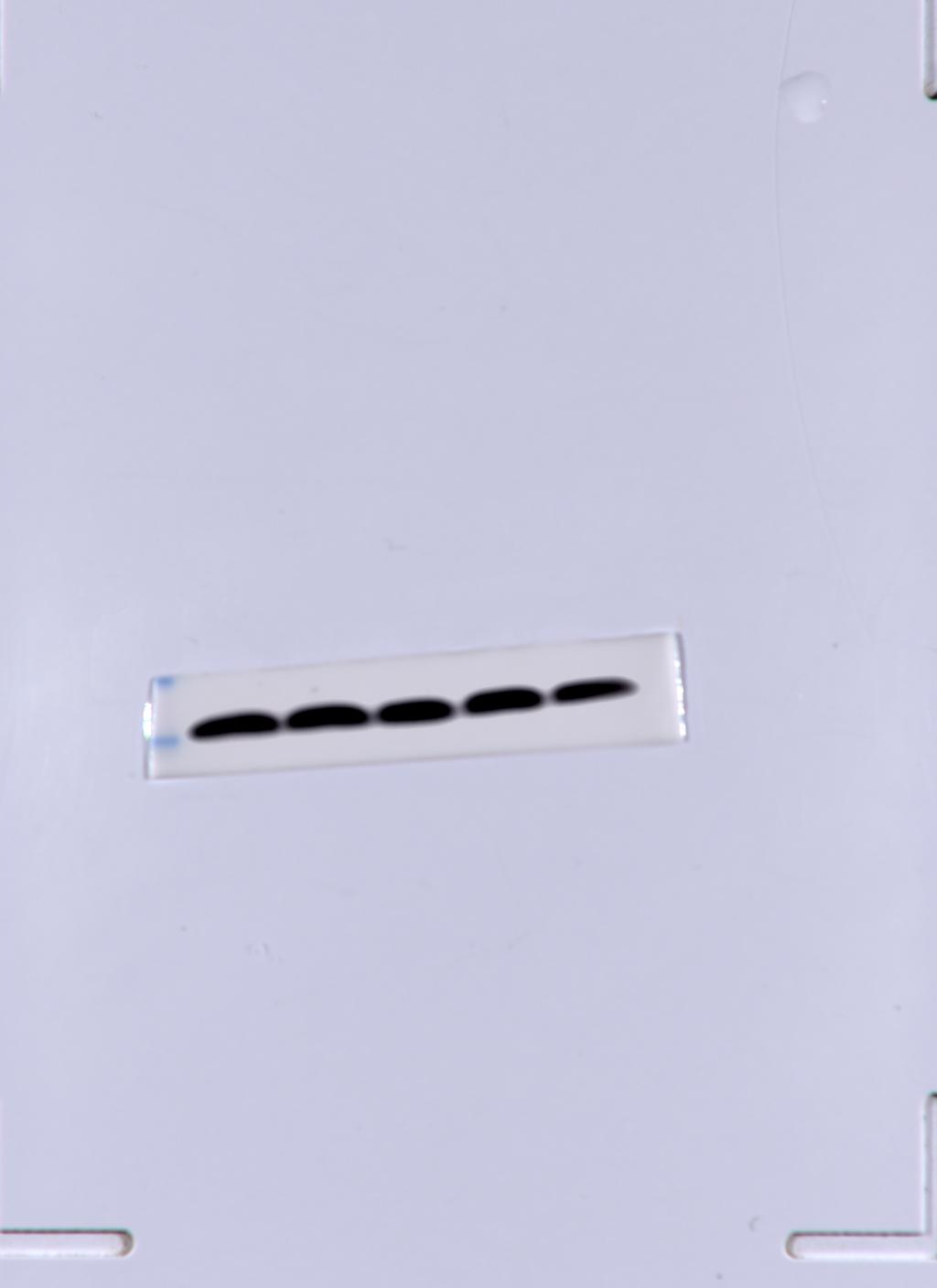
**

**cGAS β-actin**

**B**

**
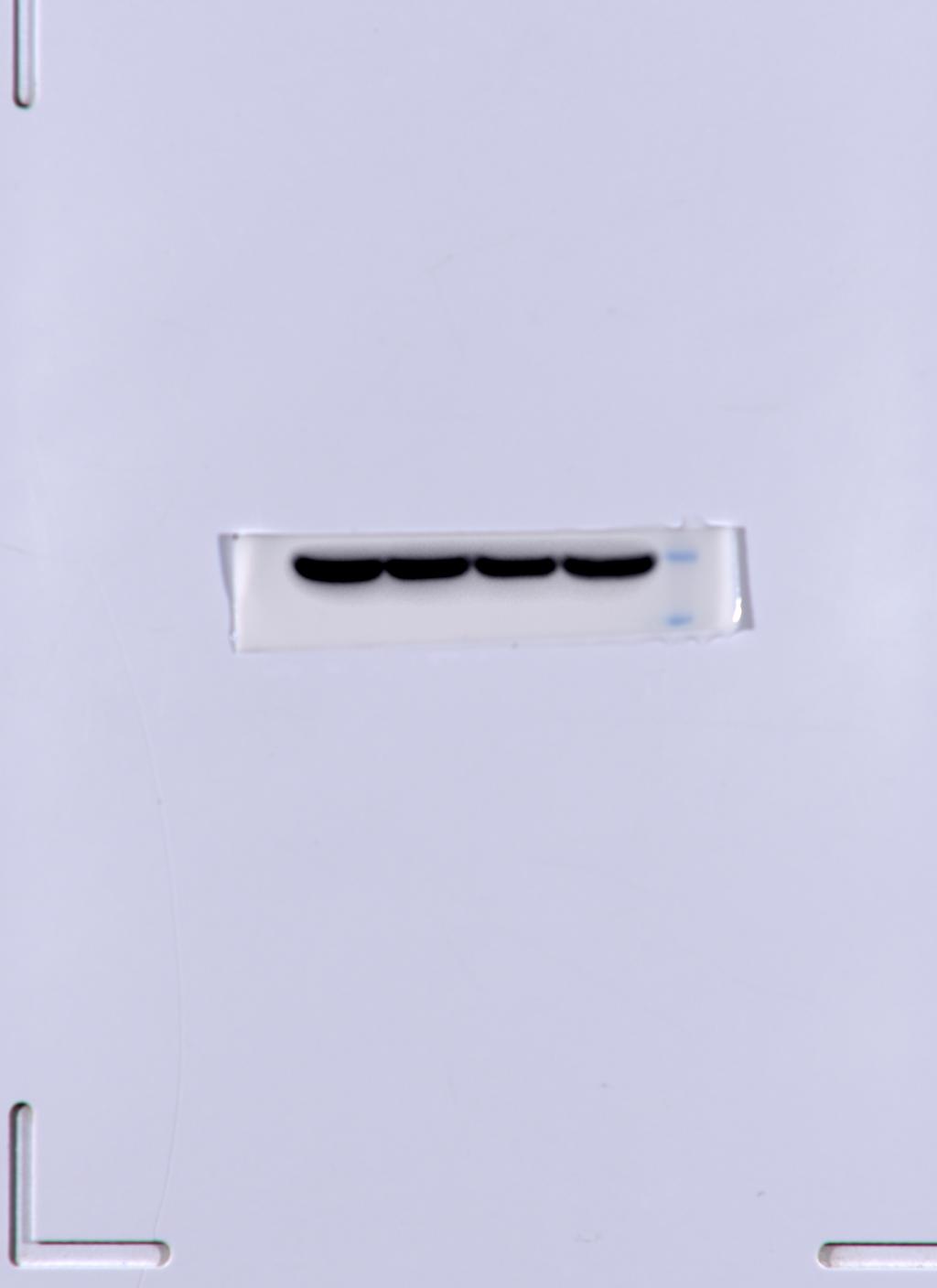

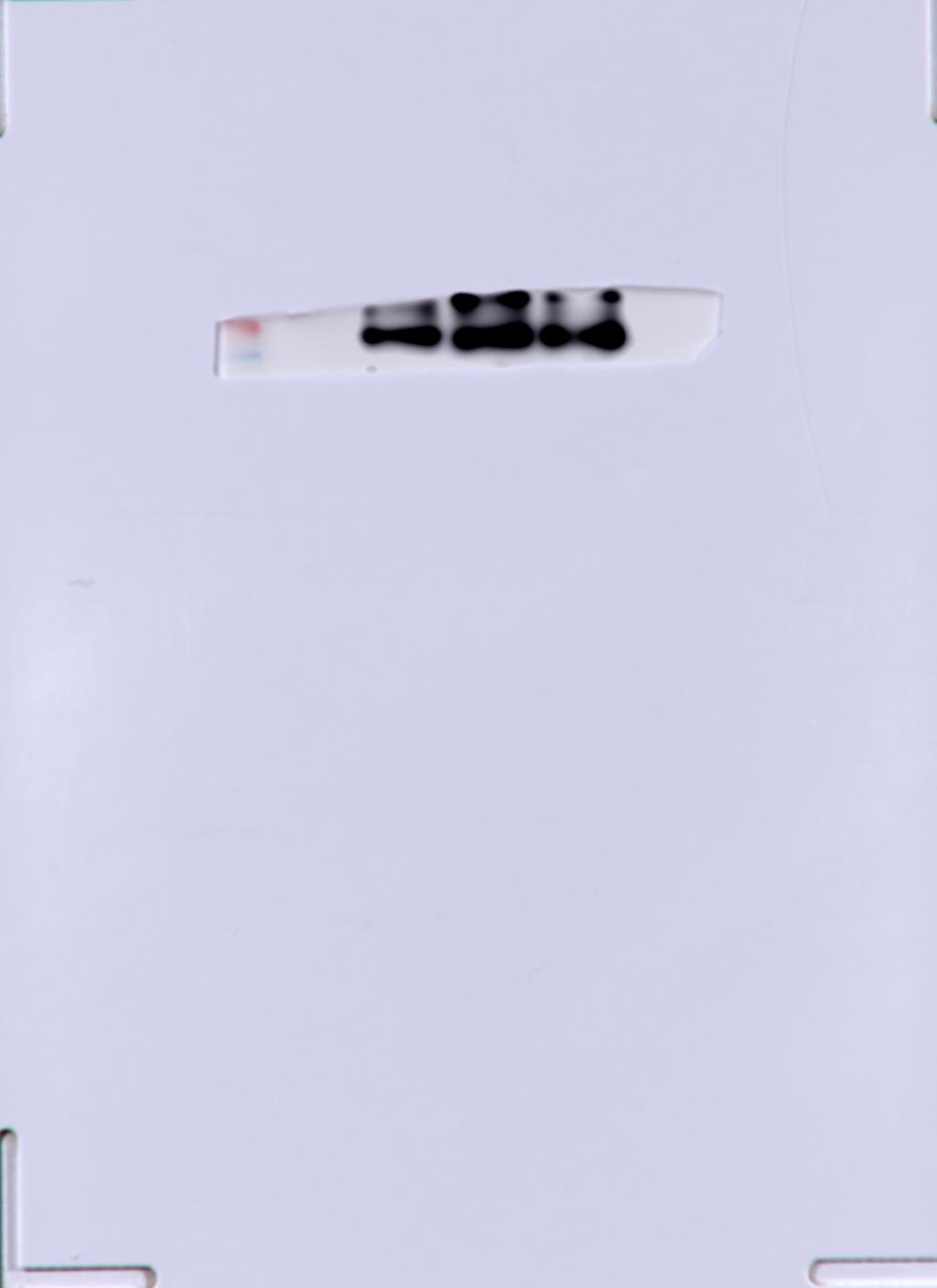

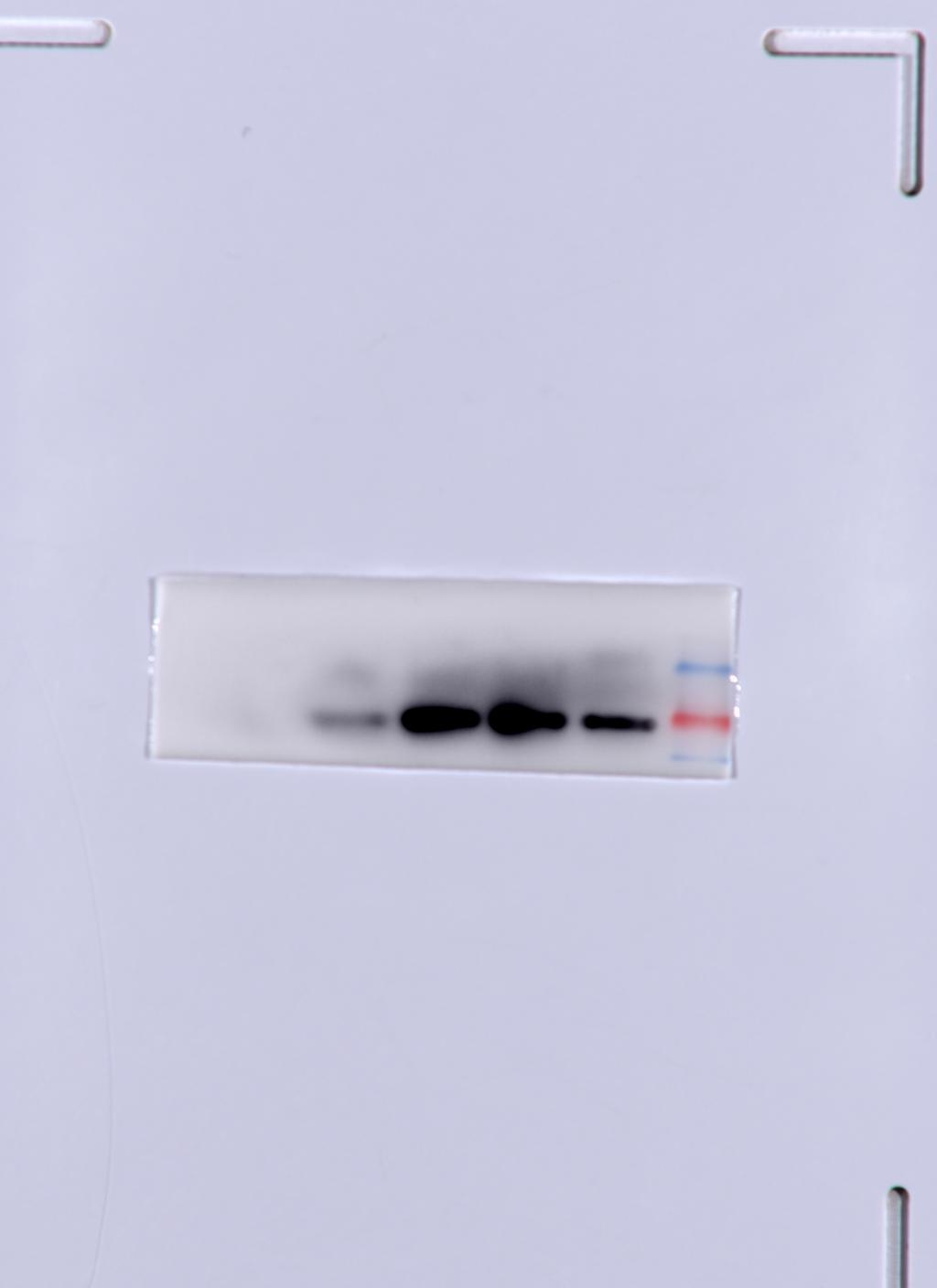
**

**β-actin Myc cGAS**

**C**

| Blank | EV | DDX56 | siNC+DDX56 | sicGAS+DDX56 |
| --- | --- | --- | --- | --- |
| 1 | 41.21031952 | 64.36989307 | 68.456563 | 21.61463547 |
| 0.91541909 | 32.19177771 | 67.16536427 | 61.71957433 | 18.76865482 |
| 1.224908924 | 36.33929443 | 63.49255753 | 57.58788681 | 15.38431644 |

**D**

| EV | DDX56 | siNC+DDX56 | sicGAS+DDX56 |
| --- | --- | --- | --- |
| 8.419765576e+007 | 3.84237723e+007 | 3.983931965e+007 | 1.271744886e+008 |
| 1.14419488e+008 | 4.959067275e+007 | 5.329932917e+007 | 1.565308703e+008 |
| 1.026360666e+008 | 5.376504331e+007 | 5.697935152e+007 | 1.366784358e+008 |
